# Performance of Automatic Speech Analysis in Detecting Depression: Systematic Review and Meta-Analysis

**DOI:** 10.2196/67802

**Published:** 2025-10-22

**Authors:** Patricia Laura Maran, María Dolores Braquehais, Alexandra Vlaic, María Teresa Alonzo-Castillo, Júlia Vendrell-Serres, Josep Antoni Ramos-Quiroga, Amanda Rodríguez-Urrutia

**Affiliations:** 1 Psychiatry, Mental Health and Addictions Group, Vall d'Hebron Research Institute (VHIR), Instituto de Investigación Sanitaria Acreditado Instituto de Investigación - Hospital Universitario Vall d'Hebron (IR-HUVH) Barcelona, Catalonia Spain; 2 Department of Psychiatry and Forensic Medicine Universitat Autònoma de Barcelona Barcelona Spain; 3 Biomedical Network Research Centre on Mental Health (CIBERSAM) Barcelona Spain; 4 Integral Care Programme for Sick Health Professionals Galatea Clinic Barcelona Spain; 5 School of Medicine Universitat Internacional de Catalunya Barcelona Spain; 6 Department of Psychiatry Vall d’Hebron Hospital Universitari Barcelona Spain

**Keywords:** depression, AI, automatic speech analysis, meta-analysis, artificial intelligence, mobile phone

## Abstract

**Background:**

Despite the high prevalence and significant burden of depression, underdiagnosis remains a persistent challenge. Automatic speech analysis (ASA) has emerged as a promising method for depression assessment. However, a comprehensive quantitative synthesis evaluating its diagnostic accuracy is still lacking.

**Objective:**

This systematic review and meta-analysis aimed to assess the diagnostic performance of ASA in detecting depression, considering both machine learning and deep learning approaches.

**Methods:**

We conducted a systematic search across 8 databases, including MEDLINE, PsycInfo, Embase, CINAHL, IEEE Xplore, ACM Digital Library, Scopus, and Google Scholar from January 2013 to April 1, 2025. We included studies published in English that evaluated the accuracy of ASA for detecting depression, and reported performance metrics such as accuracy, sensitivity, specificity, precision, or confusion matrices. Study quality was assessed using a modified version of the Quality Assessment of Studies of Diagnostic Accuracy-Revised. A 3-level meta-analysis was performed to estimate the pooled highest and lowest accuracy, sensitivity, specificity, and precision. Meta-regressions and subgroup analyses were performed to explore heterogeneity across various factors, including type of publication, artificial intelligence algorithms, speech features, speech-eliciting tasks, ground truth assessment, validation approach, dataset, dataset language, participants’ mean age, and sample size.

**Results:**

Of the 1345 records identified, 105 studies met the inclusion criteria. The pooled mean of the highest accuracy, sensitivity, specificity, and precision were 0.81 (95% CI 0.79 to 0.83), 0.84 (95% CI 0.81 to 0.86), 0.83 (95% CI 0.79 to 0.86), and 0.81 (95% CI 0.77 to 0.84), respectively, whereas the pooled mean of the lowest accuracy, sensitivity, specificity, and precision were 0.66 (95% CI 0.63 to 0.69), 0.63 (95% CI 0.58 to 0.68), 0.60 (95% CI 0.55 to 0.66), and 0.64 (95% CI 0.58 to 0.70), respectively.

**Conclusions:**

ASA shows promise as a method for detecting depression, though its readiness for clinical application as a standalone tool remains limited. At present, it should be regarded as a complementary method, with potential applications across diverse contexts. Further high-quality, peer-reviewed studies are needed to support the development of robust, generalizable models and to advance this emerging field.

**Trial Registration:**

PROSPERO CRD42023444431; https://www.crd.york.ac.uk/PROSPERO/view/CRD42023444431

## Introduction

Depression represents a significant global health challenge, ranking among the leading causes of disability and premature mortality. Approximately 280 million people worldwide are estimated to endure major depressive disorder, representing 3.8% of the global population [1]. Depression is associated with substantial societal and economic costs [2], loss in productivity [3], and a reduced quality of life [4,5]. Additionally, depression is a key risk factor for a spectrum of chronic health conditions, including arthritis, asthma, cancer, and cardiovascular diseases [6]. Notably, there is evidence that patients who endure both depression and chronic physical conditions tend to have considerably lower quality of life than those enduring a chronic physical condition solely [7], with patients with depression dying 5 to 10 years earlier due to chronic physical conditions [8]. Perhaps most alarmingly, depression is inextricably linked to suicide [9], with people enduring depression being 20 times more likely to commit suicide than the general population [5]. Consequently, the early detection of depression and its timely intervention assume paramount significance.

Current state-of-the-art depression assessment primarily relies on operational diagnostic criteria from the *DSM-5-TR* (*Diagnostic and Statistical Manual of Mental Disorders* [Fifth Edition, Text Revision]) [10] and the *ICD-11* (*International Classification of Diseases, 11th Revision*) [11]. When considering screening, the Patient Health Questionnaire-9 [12] is the most commonly used tool for depression due to its brevity, ease of use, and validation across various languages and contexts [13]. However, these assessments are susceptible to a range of biases arising from the interviewer’s experience, the quality of the question protocol, and the patient’s willingness to communicate their symptoms [14]. Field trials of the *DSM-5* criteria for major depressive disorder have revealed strikingly low interrater reliability [15]. Hence, the acquisition of diagnostic information through these traditional means is not only time-consuming but also demands considerable clinical training and practice to yield reliable results [16]. Adding to these complexities is the stark shortage of mental health professionals worldwide, which represents one of the biggest obstacles to the early detection of depression [17]. For instance, there are approximately 9 psychiatrists per 100,000 people in developed countries [18] and as few as 0.1 for every 1,000,000 in middle- and lower-income countries [19]. Therefore, there is an urgent need to objectively screen and diagnose individuals with depression, particularly those who face geographical, financial, or practical barriers to accessing traditional psychological or psychiatric services [20].

In response to these pressing needs, ongoing efforts have been made to identify efficient and objective biological, physiological, and behavioral biomarkers for depression. A wide range of biological markers, such as low serotonin levels [21], neurotransmitter dysfunction [22], genetic abnormalities [23], electroencephalogram signals [24,25], eye movements [26], gaits [27], and inflammatory biomarkers [28], have been associated with depression. Nevertheless, a specific biomarker with adequate predictive value for diagnosing depression remains elusive.

With recent advancements in the machine learning field, automatic speech analysis (ASA) has gained popularity as an attractive objective biomarker for depression assessment. ASA presents a variety of benefits that make it particularly attractive for both research and clinical applications. First, it is cost-effective, noninvasive, unobtrusive, and suitable for remote monitoring. In contrast to other biological metrics, speech can be easily collected using modern technologies, such as smartphones, tablets, and computers, which are broadly accessible to the population, thereby eliminating the need for expensive wearables or invasive neuroimaging techniques. Second, ASA may offer greater objectivity compared to self-reported measures. While self-report questionnaires are subject to bias and intentional or unintentional symptom masking, speech features, particularly acoustic features, are more difficult to consciously manipulate. This is because depression can disrupt motor and cognitive processes associated with speech production, resulting in subtle acoustic and linguistic changes that reflect underlying physiological and neurocognitive alterations. Third, speech conveys both what is said and how it is said, providing a dual perspective on the speaker’s cognitive and emotional states. While the linguistic content serves as a direct manifestation of these states, variations in motor and acoustic features can indirectly reveal underlying neural activity [20]. Finally, speech may be generalized across different languages due to the shared aspects of vocal anatomy, which allow for the comparison and application of speech-based metrics across diverse linguistic contexts (for a more in-depth review of the advantages of speech as a biomarker, see the study by Low et al [20]).

An increasing number of studies have investigated the potential of speech analysis for depression detection, which has led to the publication of several reviews [14,20,29,30]. However, to date, only Liu et al [30] has conducted a meta-analysis to evaluate the diagnostic performance of ASA to detect depression, and their review was limited to studies using deep learning algorithms. This underscores the need for a comprehensive quantitative synthesis of the evidence that includes both machine learning and deep learning approaches. Therefore, this systematic review and meta-analysis aimed to synthesize published data on the performance of ASA for depression detection.

## Methods

### Overview

This systematic review and meta-analysis were conducted in accordance with the PRISMA-DTA (Preferred Reporting Items for Systematic Reviews and Meta-Analyses—Extension for Diagnostic Test Accuracy) [31]. The PRISMA-DAT checklist is provided in Multimedia Appendix 1 [32-136]. We registered the review protocol with the PROSPERO (International Prospective Register of Systematic Reviews; ID: CRD42023444431). There was no prior published protocol for the current study.

### Search Strategy

We systematically searched 8 electronic databases on April 1, 2025, including MEDLINE (via Ovid), APA PsycInfo (via Ovid), Embase (via Ovid), CINAHL (via EBSCO), IEEE Xplore, ACM Digital Library, Scopus, and Google Scholar. For Google Scholar, only the first 100 results were considered. Our search used the following terms as index terms or free-text words: “speech analysis” and depression. The full electronic search strategy used for MEDLINE was the following: ((“speech analysis”[tw] OR “speech processing”[tw] OR “speech feature”[tw] OR “speech features”[tw] OR “speech signal”[tw] OR “speech signal processing”[tw] OR “speech classification”[tw] OR “speech recognition”[tw] OR “speech model”[tw] OR “acoustic analysis”[tw] OR “acoustic processing”[tw] OR “acoustic feature”[tw] OR “acoustic features”[tw] OR “acoustic signal”[tw] OR “acoustic signal processing”[tw] OR “acoustic classification”[tw] OR “acoustic recognition”[tw] OR “acoustic model”[tw] OR “vocal analysis”[tw] OR “vocal processing”[tw] OR “vocal feature”[tw] OR “vocal features”[tw] OR “vocal signal”[tw] OR “vocal signal processing”[tw] OR “voice recognition”[tw] OR “vocal recognition”[tw] OR “voice model”[tw] OR “vocal model”[tw]) AND (exp Depression/ OR depress*[tw] OR exp Depressive Disorder/ OR depressive disorder*[tw])). The full search strategies for all databases are detailed in Table S12 in Multimedia Appendix 2. The screening process involved 2 stages. First, titles and abstracts were reviewed, followed by a thorough examination of full-texts. The entire selection process was independently conducted by 2 reviewers (PLM and AV), and discrepancies were resolved through dialogue and consensus.

### Eligibility Criteria and Study Selection

We limited the inclusion to studies that (1) evaluated the accuracy of ASA to detect depression; (2) reported performance metrics such as accuracy, sensitivity, specificity, precision, or confusion matrices; and (3) were published in English since 2013. We excluded studies focusing on the prediction of depression severity or treatment outcomes. Studies that used multimodal data (eg, face features) in addition to speech data were excluded, except those that reported distinct performance values for each separate model. For the publication type, we included journal papers, conference papers, and thesis dissertations, but excluded reviews, preprints, conference abstracts, posters, protocols, editorials, and comments. We did not apply any constraints in terms of setting, reference standard, or country of publication.

### Data Extraction

Data extraction covered study metadata, speech variables, artificial intelligence (AI) algorithms, and performance metrics. From studies providing raw data or confusion matrices, we computed accuracy, sensitivity, specificity, and precision. Many studies have conducted multiple experiments to assess distinct speech features, validation methods, and AI techniques, yielding a range of results. To comprehensively represent this variability, we extracted both the lowest and highest values reported for each performance metric across different algorithms. Data extraction was conducted by PLM, while AV and MTA-C verified the extraction for quality assurance.

### Risk of Bias and Applicability Appraisal

Two reviewers (AV and MTA-C) independently used a modified version of the Quality Assessment of Studies of Diagnostic Accuracy-Revised (QUADAS-2) by Abd-Alrazaq et al [137] to evaluate the risk of bias and applicability of the included studies. Similar to the original QUADAS-2, the modified version covered 4 domains: participants, index test (AI algorithms), reference standard (ground truth), and analysis [137]. Disagreements were resolved through either consensus or adjudication by a third reviewer (PLM).

### Statistical Analysis

Given that many studies conducted more than 1 experiment and reported multiple performance metrics, there was a potential for these studies to disproportionately influence the meta-analysis compared to those reporting a single result. Moreover, experiments from the same study cannot be regarded as independent, which challenges the independence assumption for effect sizes, underlying traditional meta-analytic techniques [138]. Therefore, to calculate the pooled mean of the highest and lowest accuracy, sensitivity, specificity, and precision, we used a 3-level meta-analysis using restricted maximum likelihood [139]. This approach accounts for 3 distinct sources of variance: population differences between study population effects, population differences between effects of experiments from the same study, and, finally, sampling variance [140]. Anticipating significant heterogeneity, we applied a random-effects model.

Meta-regression and subgroup analyses were conducted to assess potential variations in ASA performance across different factors, including type of publication, AI algorithms, speech features, speech-eliciting tasks, ground truth assessment, validation approach, dataset, and dataset language, participants’ mean age, and sample size. To ensure the robustness of our findings, we restricted our analyses to subgroups containing 5 or more estimates [141]. To quantify and assess the study heterogeneity, we used the Cochran *Q*-test and *I*^2^. Cochran *Q* test determines whether the observed variability exceeds the study’s sampling error alone, and a Cochran *P* value ≤.05 indicates statistically significant heterogeneity [142]. *I*^2^ statistics estimate the proportion of total variation due to true heterogeneity rather than sampling error, and values of 25%, 50%, and 75% reflect low, moderate, and high heterogeneity, respectively [143]. All analyses were performed using the *metafor* package in R [144,145].

## Results

### Study Selection

**Figure 1:**
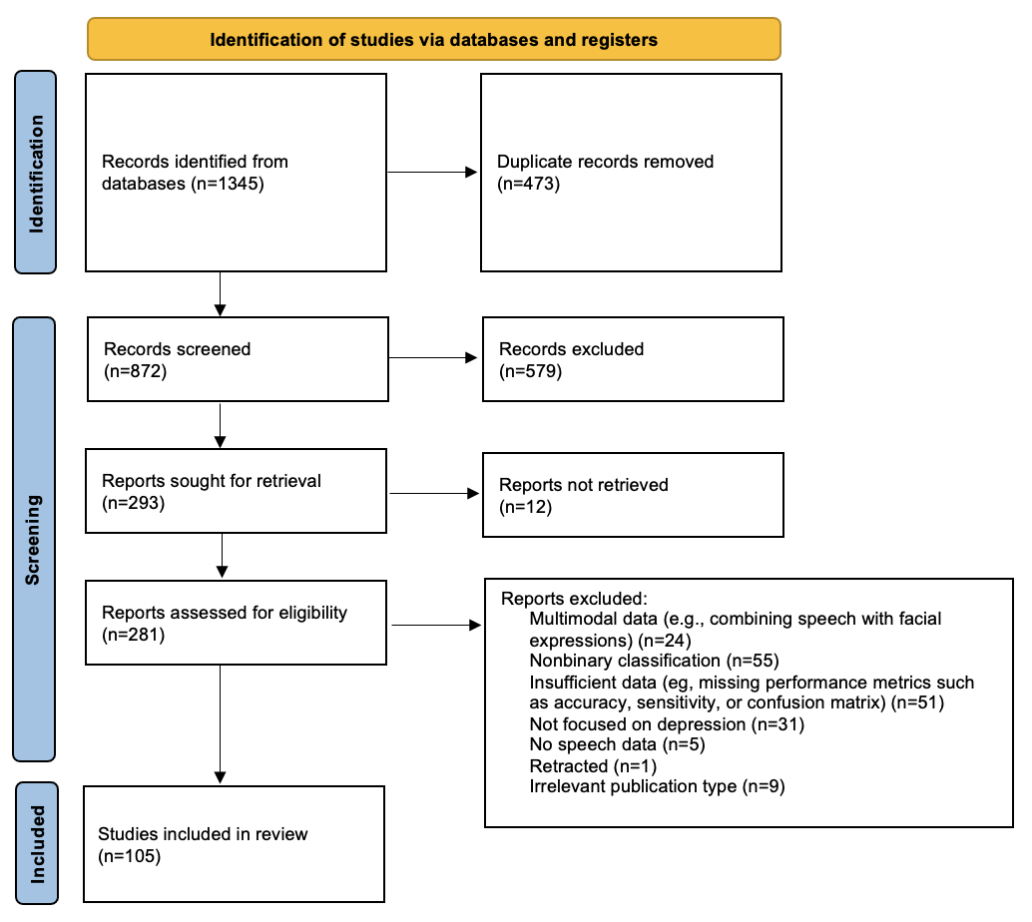
PRISMA flow diagram of the study selection process. This diagram describes the process of identifying, screening, and selecting studies for inclusion. Initially, a total of 1345 records were identified from databases. After the removal of 473 duplicates, 872 records remained for the screening phase. Of these, 579 records were excluded, and 293 reports were sought for retrieval. Further, 12 reports could not be retrieved, resulting in 281 reports assessed for eligibility. Ultimately, 176 reports were excluded based on the predefined inclusion criteria. The final review included 105 studies. PRISMA: Preferred Reporting Items for Systematic Reviews and Meta-Analyses.

The initial search through 8 databases yielded a total of 1345 studies. After removing 473 duplicates, 872 papers remained. Following titles and abstract screening, a further 579 publications were excluded. Twelve publications were not retrieved. After reading the full text of all the remaining 293 records, a total of 105 studies were ultimately included in the current review. Figure 1 shows the process of the study selection process.

### General Study Characteristics

The 105 studies included in this review were published between 2013 and 2025, with 21 (20%) published in 2022 and 2023. A total of 33 (31.4%) studies were carried out in China, 12 (11.4%) in India, and 10 (9.5%) in Hungary. Regarding the publication type, the majority (55/105, 52.4%) were conference papers, followed by peer-reviewed journal papers (48/105, 45.7%), and theses (2/105, 1.9%). The number of participants included was reported in 98 studies, with sample sizes ranging from 14 to 9337. The age of participants, reported in 34 studies, ranged between 19.0 and 71.7 years, with an average age of 37.47 (SD 10.41) years. The percentage of female participants, reported in 53 studies, varied between 30.8% and 100%. The percentage of depressed participants, as reported in 57 studies, ranged between 21% and 69%, with an average of 45.9% (SD 11.9). Table 1 and Table S2 in Multimedia Appendix 2 detail the specific characteristics of the included studies.

**Table 1:** Characteristics of the included studies.

Feature				Values		References
**Year of publication**						
		2025		4 (3.8)^a^		[32-35]
		2024		13 (12.4)^a^		[36-48]
		2023		21 (20)^a^		[49-69]
		2022		21 (20)^a^		[70-90]
		2021		13 (12.4)^a^		[91-103]
		2020		6 (5.7)^a^		[104-109]
		2019		6 (5.7)^a^		[110-115]
		2018		4 (3.8)^a^		[116-119]
		2017		7 (6.7)^a^		[120-126]
		2016		5 (4.8)^a^		[127-131]
		2015		1 (1)^a^		[132]
		2014		2 (1.9)^a^		[133,134]
		2013		2 (1.9)^a^		[135,136]
**Country of publication**						
		China		33 (31.4)^a^		[32,37,44,45,48-51,54,58,60,64,65,69-75,78,79,83,91,96,102,103,118,120-122,130,136]
		India		12 (11.4)^a^		[42,52,55-57,66,81,94,104,105,114,123]
		Hungary		10 (9.5)^a^		[86,88,93,98,99,107,116,124-126]
		Australia		6 (5.7)^a^		[111,112,117,131,132,135]
		United States		6 (5.7)^a^		[38,40,89,119,129,133]
		Malaysia		5 (4.8)^a^		[84,87,101,115,127]
		Republic of Korea		4 (3.8)^a^		[34,47,59,97]
		United Kingdom		4 (3.8)^a^		[35,43,53,109]
		France		3 (2.9)^a^		[80,95,106]
		Germany		3 (2.9)^a^		[41,67,76]
		Turkey		3 (2.9)^a^		[39,63,108]
		Canada		2 (1.9)^a^		[63,128]
		Iran		2 (1.9)^a^		[62,77]
		Japan		2 (1.9)^a^		[36,82]
		Others (Brazil, Denmark, Indonesia, Italy, Philippines, Romania, Saudi Arabia, Singapore, Spain, and Thailand)		1 (each; 0.95)^a^		[33,61,68,85,90,92,100,110,113,134]
**Type of publication**						
		Conference paper		55 (52.4)^a^		[33, 35, 36, 42, 43, 45-47, 50, 52, 55-57, 61, 62, 64-66, 73-75, 83, 87-90, 92-95, 98, 101, 104, 105, 107, 110, 112-116, 119, 121-125, 127-130, 132-136]
		Journal paper		48 (45.7)^a^		[32, 34, 37-41, 44, 48, 49, 51, 53, 54, 58-60, 63, 65, 67-72, 76-82, 84-86, 91, 96, 97, 99, 100, 102, 103, 106, 108, 111, 117, 118, 120, 126]
		Thesis		2 (1.9)^a^		[109,131]
**Number of participants**						
		Mean (SD)		445.14 (1361.12)		[32-51,53-97,99-101,103,104,107-129,131-133,135,136]
		Range		14-9337		[32-51,53-97,99-101,103,104,107-129,131-133,135,136]
**Age of participants**						
	Mean (SD)		37.47 (10.41)		[32,39,41,44,45,48,53,54,58-60,63,67,69-72,74,76,79,80,82-86,96,97,100,107,110,116,122,127,128]	
	Range		19.0-71.7		[49,51,58,60,64,66,68,70-97,99-101,103,104,107-129,131-133,135,136]	
**Gender (Female, %)**						
	Mean (SD)		60.8 (15.6)		[35,36,39,41,42,44,45,48,53,54,56-59,61,63,67,68,76,79,80,82-89,96,97,100,112,118-122,125-129,133]	
	Range		30.8-1.00		[35,36,39,41,42,44,45,48,53,54,56-59,61,63,67,68,76,79,80,82-89,96,97,100,112,118-122,125-129,133]	
**Depressed participants (%)**						
	Mean (SD)		45.9 (11.9)		[33-35, 38-45, 47-51, 53-59, 63-65, 67-76, 78-84, 87-90, 92-94, 96, 99, 100, 103, 104, 107, 110, 112, 116-122, 124, 125, 127-129, 131-133, 135, 136]	
	Range		21.0-69.0		[33-35, 38-45, 47-51, 53-59, 63-65, 67-76, 78-84, 87-90, 92-94, 96, 99, 100, 103, 104, 107, 110, 112, 116-122, 124, 125, 127-129, 131-133, 135, 136]	

^a^Studies, n (%).

### Features of ASA Classifiers

In terms of speech features, spectral features (91/105, 86.7%) were the most frequently analyzed, followed by prosodic features (58/105, 55.2%), source features (53/105, 50.5%), format features (39/105, 37.1%), and lexical features (15/105, 14.3%). The most commonly used speech-eliciting task was free speech (76/105, 72.4%). Reading tasks were used in 36.2% (38/105) of studies, while only a few used counting (3/105, 2.9%) or sustained vowels (2/105, 1.9%). Many studies conducted multiple experiments to evaluate various AI algorithms, with support vector machine (SVM) being the most frequently used (43/105, 41%). The most commonly used assessment instruments to establish ground truth were the Patient Health Questionnaire-8/-9 (51/105, 48.6%). The included studies applied 4 different validation methods, the most common of which were hold-out cross-validation (64/105, 61%) and K-fold cross-validation (38/105, 36.2%). More than half of the studies used hand-crafted datasets (56/105, 53.3%), while the DAIC-WOZ (Distress Analysis Interview Corpus–Wizard of Oz) was the most frequently used public database (35/105, 33.3%). The specific details of the models used in the included studies are detailed in Table 2 and Table S2 in Multimedia Appendix 2.

**Table 2:** Features of automatic speech analysis classifiers.

Feature			Studies, n (%)
**Speech features**			
	Spectral features	91 (86.7)	
	Prosodic features	58 (55.2)	
	Source features	53 (50.5)	
	Format features	39 (37.1)	
	Lexical features	15 (14.3)	
	TEO^a^	8 (7.6)	
	Spectogram	6 (5.7)	
**Speech-eliciting tasks**			
	Free speech	76 (72.4)	
	Reading	38 (36.2)	
	Counting	3 (2.9)	
	Sustained vowels	2 (1.9)	
	Not reported	10 (9.5)	
**AI^b^ algorithms**			
	Support vector machine	43 (41.0)	
	Convolutional neural network	15 (14.3)	
	Logistic regression	14 (13.3)	
	Random forest	11 (10.5)	
	Deep neural network	10 (9.5)	
	Gaussian mixture models	9 (8.6)	
	Ensemble model	7 (6.7)	
	K-nearest neighbors	7 (6.7)	
	Multilayer perceptron	7 (6.7)	
	Naïve bayes	7 (6.7)	
	AdaBoost decision tree	3 (2.9)	
	Artificial neural network	2 (1.9)	
	Linear discriminant analysis	2 (1.9)	
	Long-term and short-term memory	3 (2.9)	
	Recurrent neural network	2 (1.9)	
	Support vector machine + Gaussian mixture model	2 (1.9)	
	Others	28 (26.7)	
**Ground truth assessment**			
	PHQ-8^c^ and PHQ-9^d^	51 (48.6)	
	BDI^e^ and BDI-II^f^	23 (21.9)	
	HAM-D^g^	10 (9.5)	
	*DSM*^h^ and *DSM-IV*^i^	10 (9.5)	
	CIDI^j^	4 (3.8)	
	MINI^k^	3 (2.9)	
	Others	9 (8.6)	
	Not reported	8 (7.6)	
**Validation approach**			
	Hold-out cross-validation	64 (61.0)	
	K-fold cross-validation	38 (36.2)	
	Leave-one-out cross-validation	18 (17.1)	
	Nested cross-validation	2 (1.9)	
	Not reported	4 (3.8)	
**Dataset**			
	Handcrafted	56 (53.3)	
	DAIC-WOZ^l^	35 (33.3)	
	AVEC-2013^m^, AVEC-2014, AVEC-2017, and AVEC-2019	10 (9.5)	
	MODMA^n^	10 (9.5)	
	CONVERGE^o^	3 (2.9)	
	DEPISDA^p^	3 (2.9)	
	ORI-DB^q^	2 (1.9)	
	Others	8 (7.6)	

^a^TEO: Teager Energy Operator.

^b^AI: artificial intelligence.

^c^PHQ-8: Patient Health Questionnaire-8.

^d^PHQ-9: Patient Health Questionnaire-9.

^e^BDI: Beck Depression Inventory.

^f^BDI-II: Beck Depression Inventory-II.

^g^HAM-D: Hamilton Depression Rating Scale.

hDSM: Diagnostic and Statistical Manual of Mental Disorders.

iDSM-IV: Diagnostic and Statistical Manual of Mental Disorders, Fourth Edition.

^j^CIDI: Composite International Diagnostic Interview.

^k^MINI: Mini-International Neuropsychiatric Interview.

^l^DAIC-WOZ: Distress Analysis Interview Corpus Wizard-of-Oz.

^m^AVEC: Audio or Visual Emotion Challenge.

^n^MODMA: Multimodal Open Dataset for Mental Disorder Analysis.

^o^CONVERGE: China, Oxford, and Virginia Commonwealth University Experimental Research on Genetic Epidemiology.

^p^DEPISDA: Hungarian Depressed Speech Database.

^q^ORI-DB: Oregon Research Institute Database.

### Results of Risk of Bias Appraisal

Almost half of the studies (50/105, 47.6%) were classified as high risk in at least 1 domain. Most of the studies (85/105, 81%) used an appropriate consecutive or random sampling method to select eligible participants. A significant portion of the studies (64/105, 61%) avoided inappropriate exclusions, and a balanced representation of depressed versus nondepressed subgroups was maintained in 60% (63/105) of them. Additionally, 66.7% (70/105) included a sufficient sample size. Thus, in the participant domain, almost half (49/105, 46.7%) of the studies had unclear or high risk of bias.

All the studies (105/105, 100%) thoroughly described the AI models used. Additionally, the predictive features (ie, speech features) were clearly outlined in 94.3% (99/105) of the cases, and 93.3% (98/105) of the studies assessed these features consistently for all participants. Nonetheless, in 74.3% (78/105) of the studies, information was insufficient to confirm that feature collection was blinded to outcome data. Risk of bias related to the index test was deemed low in 94.3% (99/105) of the studies.

Most of the studies (96/105, 91.4%) used appropriate reference standards for classifying the outcome (ie, depressed vs nondepressed). Consistent outcome definition was maintained for all participants in 87.6% (92/105) of the studies, and 86.7% (91/105) of the studies determined the outcome without predictor information. However, more than three-quarters (85/105, 81%) of the studies did not provide sufficient data to ensure that an appropriate interval was maintained between the index test and the reference standard. Accordingly, the risk of bias due to the reference standard was low in 81% (85/105) of the studies.

Only 21.9% (23/76) of the studies included all enrolled participants in the data analysis. In 93.3% (102/105) of the studies, there was insufficient information to confirm proper data preprocessing. The breakdown of the training, validation, and test sets was adequate in 91.4% (96/105) of the studies, and model performance was evaluated using appropriate metrics in 81.9% (86/105) of the studies. Consequently, 43.8% (46/105) of the studies were considered to have a low risk of bias in the analysis domain.

Regarding the evaluation of model applicability, most of the studies, 5 (97/105, 92.4%), were identified as having a low concern of applicability in the participants’ domain, and almost all studies (102/105, 97.1%) had a low concern of applicability in the index test domain. In the outcome domain, 92.4% (97/105) of the studies were adjudged to have low concerns regarding outcome definition, timing, or outcome determination. Figure S1 in Multimedia Appendix 2 illustrates the overall risk of bias and applicability concerns assessment. Table S3 in Multimedia Appendix 2 details reviewers’ assessment of each study included.

### Meta-Analysis of Included Models

#### Overview

A 3-level meta-analysis summarized the highest and lowest results for accuracy, sensitivity, specificity, and precision. Meta-regressions and subgroup analyses were conducted to examine potential differences in the ASA performance across various factors, including type of publication, AI algorithms, speech features, speech-eliciting tasks, ground truth assessment, validation approach, dataset, dataset language, participants’ mean age, and sample size. All meta-regression and subgroup analyses results are shown in Tables S4-S11 in Multimedia Appendix 2.

#### Accuracy

**Figure 2:**
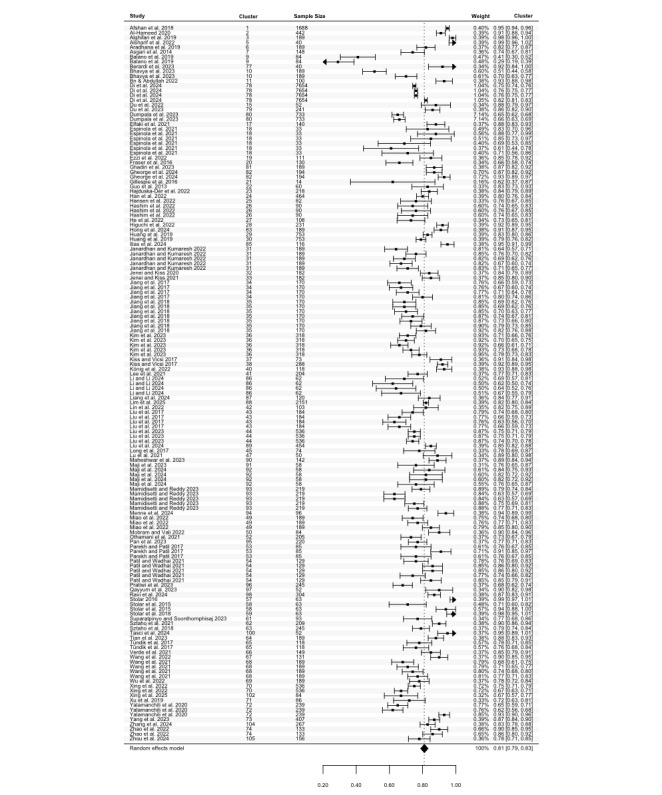
Three-level forest plot of the highest accuracy estimates. This forest plot illustrates the results of this 3-level meta-analysis based on 86 studies, comprising 148 estimates [32-34,36,37,39-49,51,53-68,71-74,77-87,89-97,99-101,104,107,109-126,128,129,131-133,136]. The solid squares represent point estimates of the highest accuracy, with horizontal lines indicating the 95% CIs. The rhombus at the bottom represents the pooled highest accuracy estimates.

**Figure 3:**
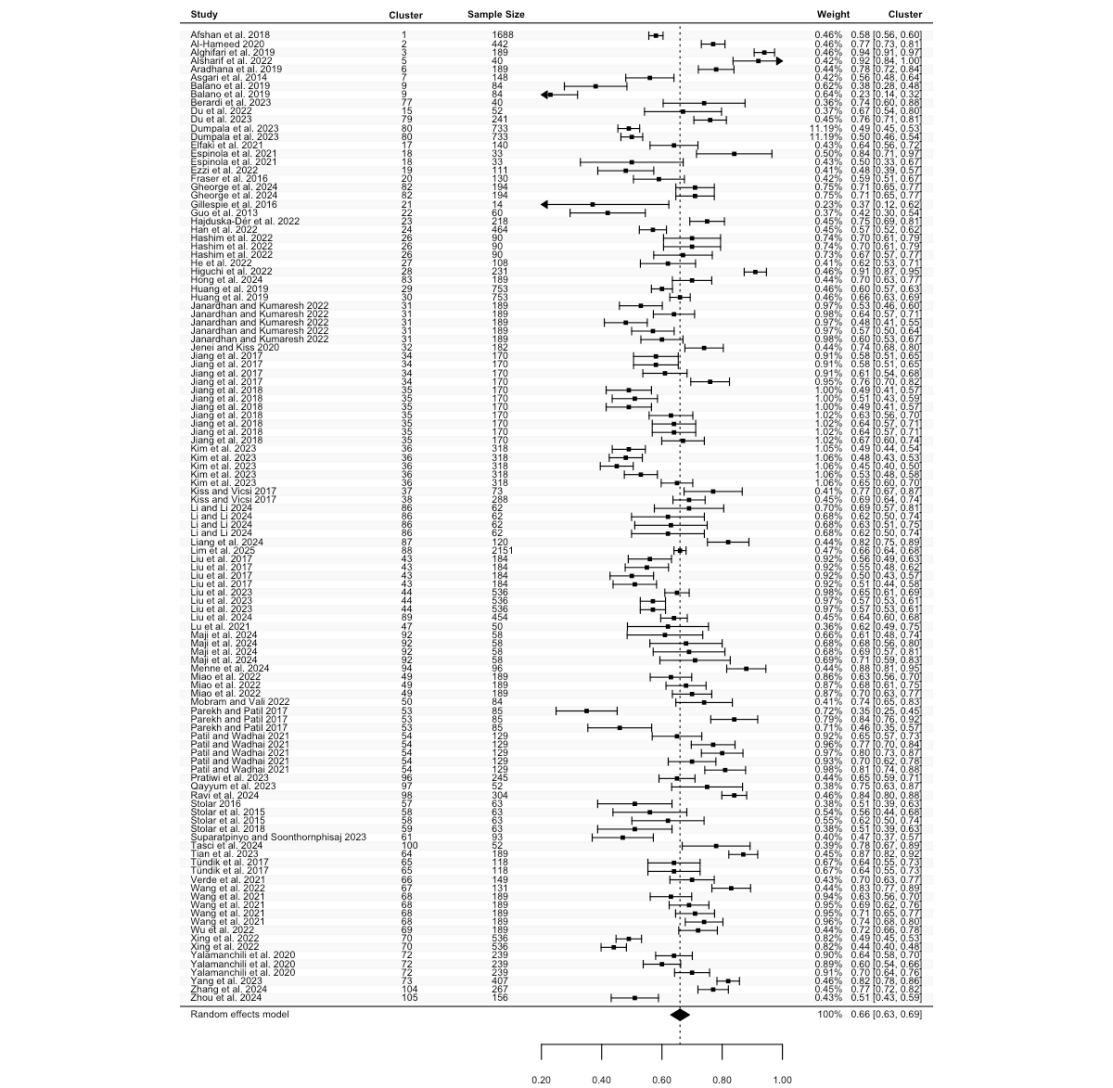
Three-level forest plot of the lowest accuracy estimates. This forest plot illustrates the results of this 3-level meta-analysis based on 114 estimates of the lowest accuracy, reported in 65 studies [33, 34, 36, 37, 39-45, 47, 49, 51, 53, 58-61, 63-65, 67, 68, 72-74, 77, 78, 81-84, 86, 87, 90-92, 94, 96, 100, 101, 104, 107, 109, 111-115, 117-121, 123-126, 128, 129, 131-133, 136]. The solid squares represent point estimates of accuracy, with horizontal lines indicating the 95% CIs. The rhombus at the bottom represents the pooled lowest accuracy estimates.

Accuracy was reported in 86 studies, comprising 148 estimates (N=27,039). The highest accuracy ranged from 0.29 to 0.99, with a pooled mean of 0.81 (95% CI 0.79 to 0.83; Figure 2). There was significant heterogeneity among studies (Cochran *P*<.001; *I*^2^=96.74%). The lowest accuracy estimates, derived from 114 estimates in 65 studies (N=16,394), ranged between 0.23 and 0.94. The pooled mean for lowest accuracy was 0.66 (95% CI 0.63 to 0.69; Figure 3), also showing considerable heterogeneity (Cochran *P*<.001; *I*^2^=94.44%). Meta-regression and subgroup analyses revealed statistically significant differences in the highest accuracy for speech features (Cochran *P*=.04) and algorithms (Cochran *P*=.04) groups (Table S4 in Multimedia Appendix 2). No other statistically significant differences were observed.

#### Sensitivity

**Figure 4:**
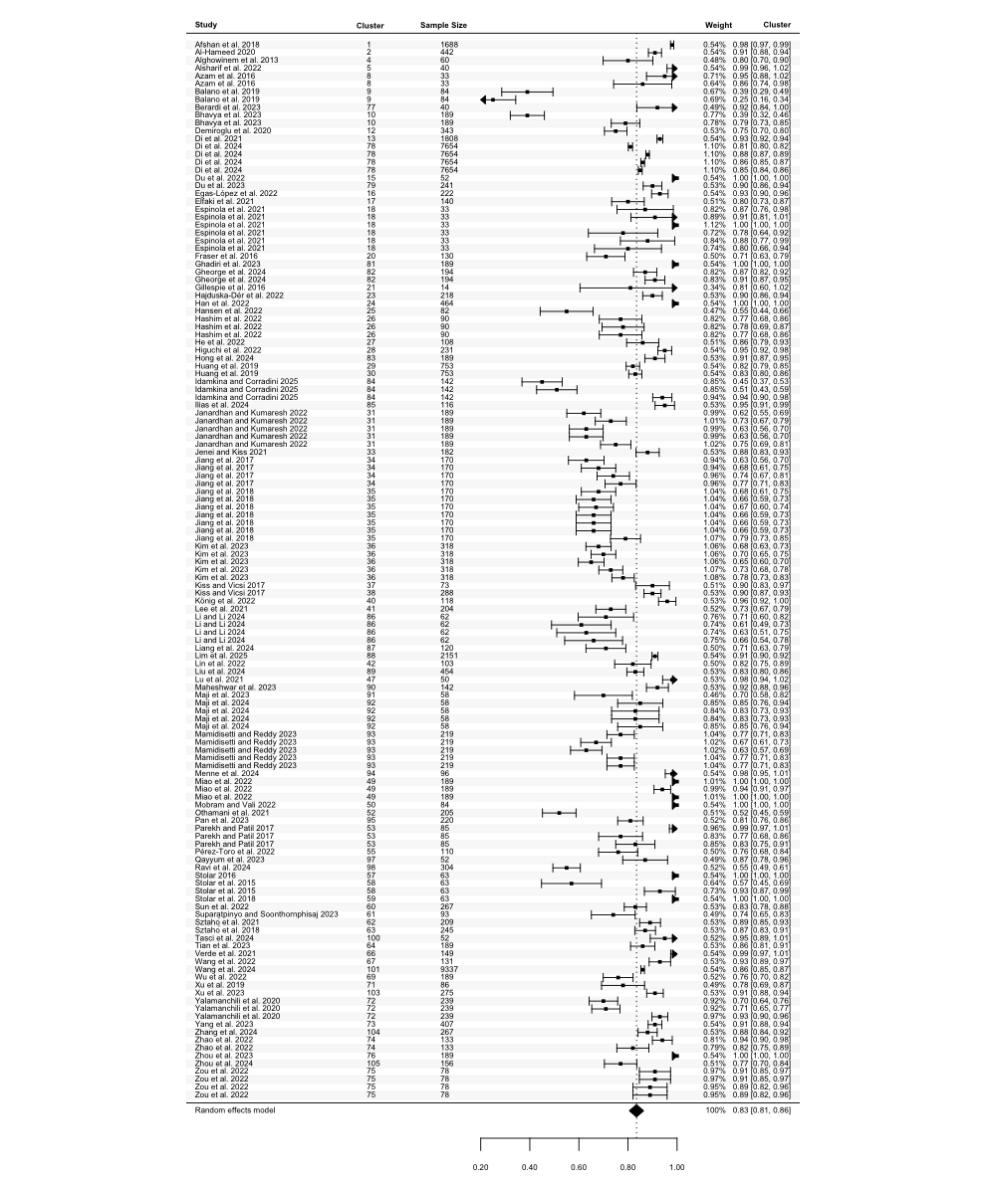
Three-level forest plot of the highest sensitivity estimates. This forest plot illustrates the results of this 3-level meta-analysis based on 135 estimates of the highest sensitivity, reported in 81 studies [34-51, 53-62, 64-71, 73-86, 88, 90, 92, 93, 95-97, 99-101, 103, 104, 108-110, 112, 113, 116-120, 123, 125-129, 131, 132, 135]. The solid squares represent point estimates of sensitivity, with horizontal lines indicating the 95% CIs. The rhombus at the bottom represents the estimated pooled highest sensitivity.

**Figure 5:**
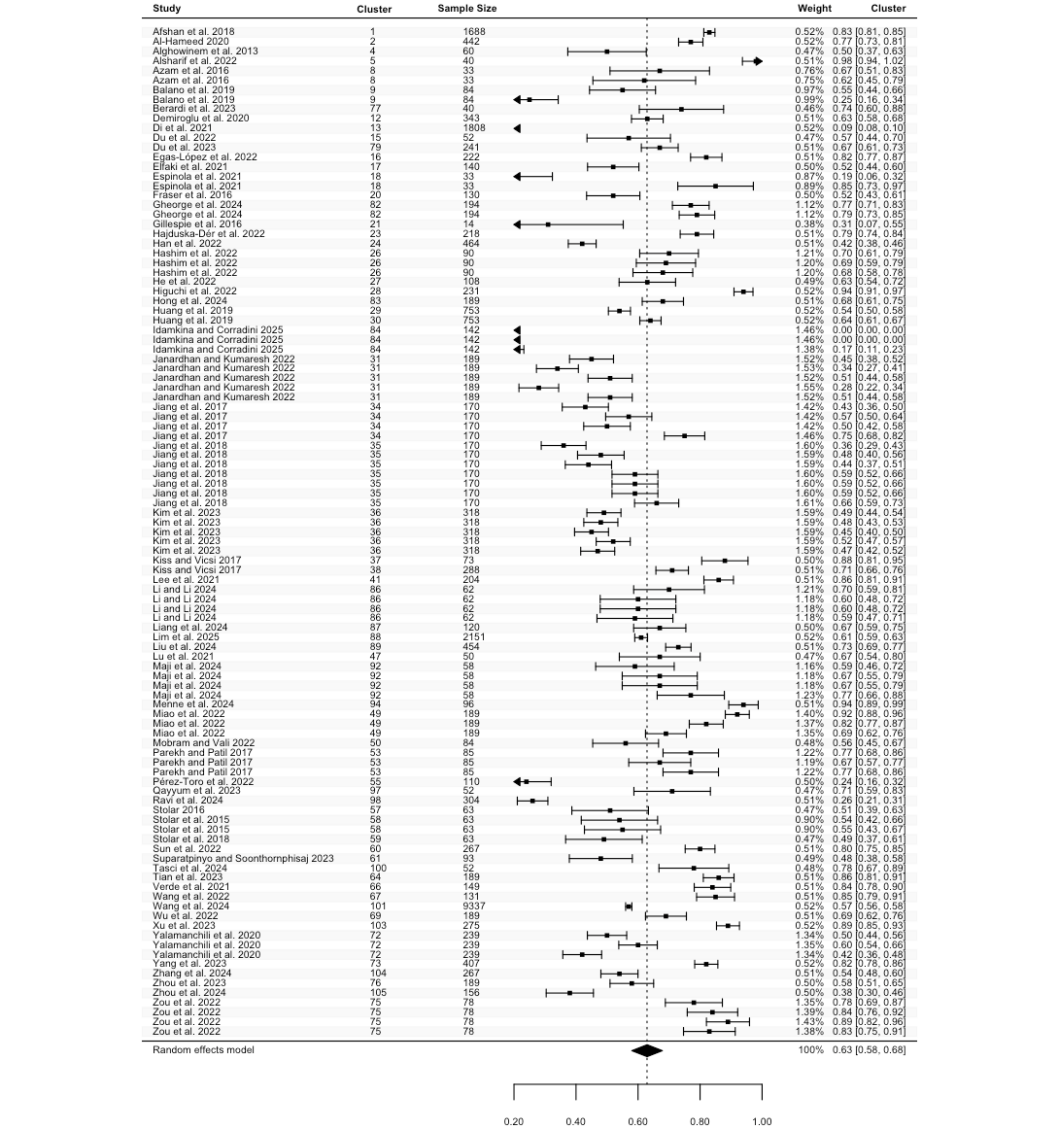
Three-level forest plot of the lowest sensitivity estimates. This forest plot illustrates the results of this 3-level meta-analysis based on 105 estimates of the lowest sensitivity, from 64 studies [34-45, 47, 49-51, 53, 55, 59-61, 64, 65, 67-70, 73-78, 81-84, 86, 88, 90, 92, 96, 97, 100, 101, 103, 104, 108, 109, 112, 113, 117-120, 123, 125-129, 131, 132, 135]. The solid squares represent point estimates of sensitivity, with horizontal lines indicating the 95% CIs. The rhombus at the bottom represents the estimated pooled lowest sensitivity.

Sensitivity data were reported in 81 studies, including 135 estimates (N=36,096). The highest sensitivity ranged from 0.25 to 1.00, with a pooled mean of 0.84 (95% CI 0.81 to 0.86; Figure 4), and considerable heterogeneity (Cochran *P*<.001; *I*^2^=99.93%). For the lowest sensitivity, 105 estimates from 64 studies (N=25,913) ranged between 0.00 and 0.98. The pooled mean was 0.63 (95% CI 0.58 to 0.68; Figure 5), with significant heterogeneity (Cochran *P*<.001; *I*^2^=99.93%). Meta-regression and subgroup analyses revealed no statistically significant differences in sensitivity across groups except for speech features subgroups in the highest sensitivity (*P*=.05; Table S6 in Multimedia Appendix 2).

#### Specificity

**Figure 6:**
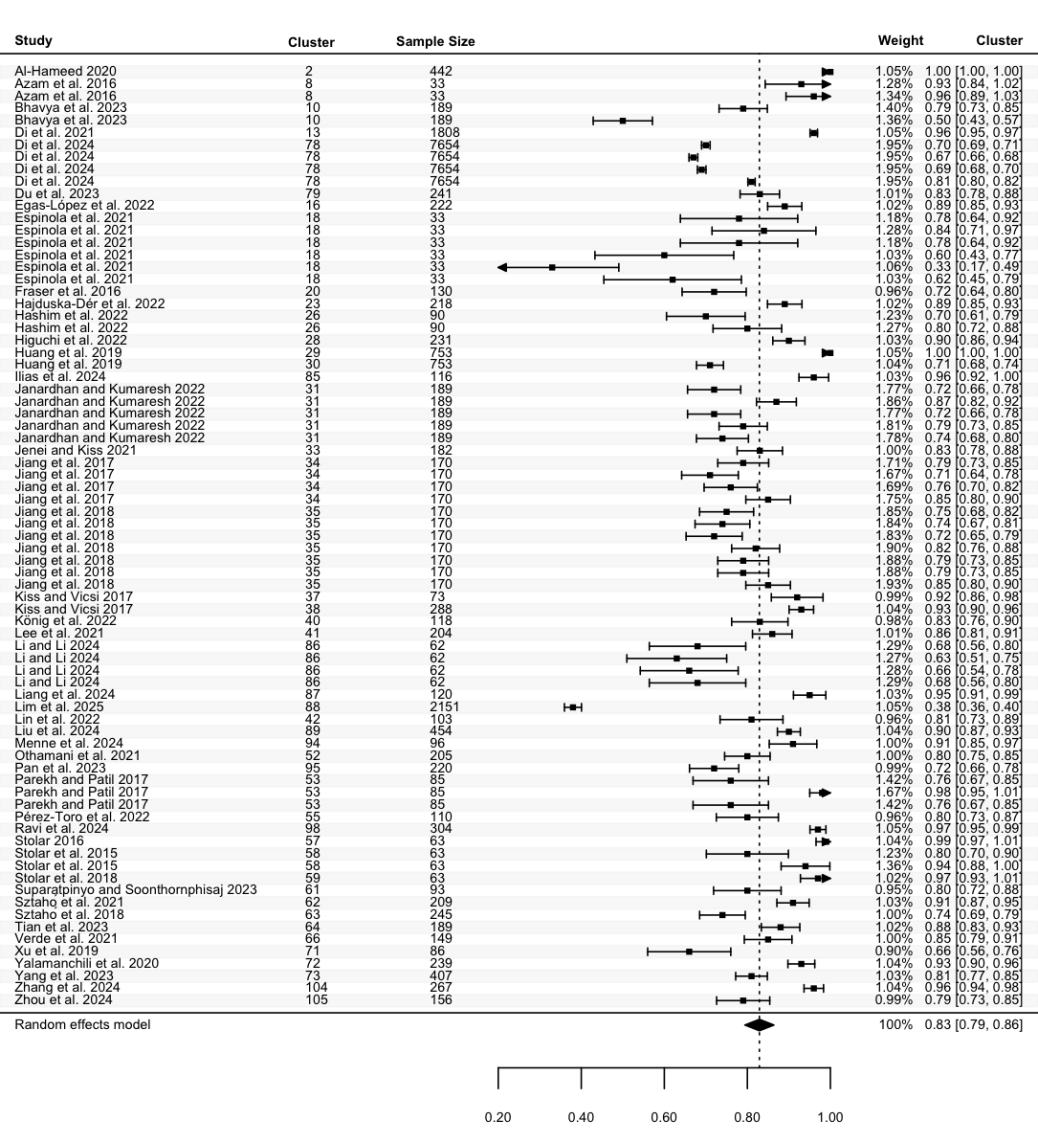
Three-level forest plot of the highest specificity estimates. This forest plot illustrates the results of this 3-level meta-analysis based on 77 estimates of the highest specificity, from 47 studies [34, 36, 37, 40, 41, 43-46, 48, 49, 51, 54, 65, 66, 68, 76, 79-82, 84, 86, 88, 92, 93, 95, 97, 99, 100, 103, 104, 109-112, 116-118, 120, 123, 125-128, 131, 132]. The solid squares represent point estimates of specificity, with horizontal lines indicating the 95% CIs. The rhombus at the bottom represents the estimated pooled highest specificity.

**Figure 7:**
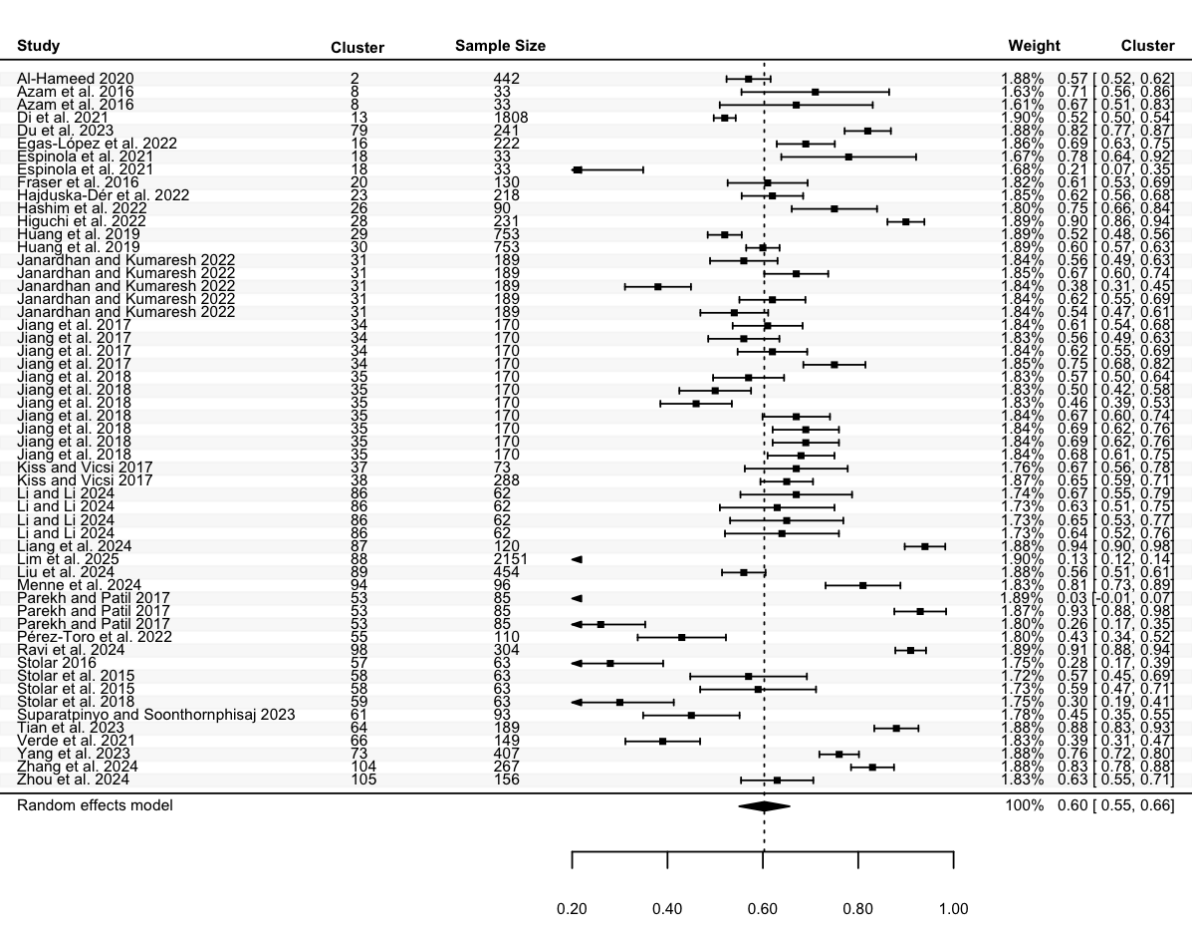
Three-level forest plot of the lowest specificity estimates. This forest plot illustrates the results of this 3-level meta-analysis based on 55 estimates of the lowest specificity, from 34 studies [34, 36, 37, 40, 41, 43-45, 49, 51, 65, 68, 76, 81, 82, 84, 86, 88, 92, 100, 103, 109, 111, 112, 117, 118, 120, 123, 125-128, 131, 132]. The solid squares represent point estimates of specificity, with horizontal lines indicating the 95% CIs. The rhombus at the bottom represents the estimated pooled lowest specificity.

Specificity was reported in 47 studies, with a total of 77 estimates (N=20,207). The highest specificity ranged from 0.33 to 1.00, with a pooled mean of 0.83 (95% CI 0.79 to 0.86; Figure 6), and high heterogeneity (Cochran *P*<.001; *I*^2^=99.81%). The lowest specificity estimates, from 55 estimates in 34 studies (N=10,553), ranged between 0.03 and 0.94. The pooled mean was 0.60 (95% CI 0.55 to 0.66; Figure 7), with significant heterogeneity (Cochran *P*<.001; *I*^2^=97.81%). Meta-regression and subgroup analyses indicated no statistically significant differences in specificity across groups except for speech features subgroups in the highest specificity (*P*=.004; Table S8 in Multimedia Appendix 2).

#### Precision

**Figure 8:**
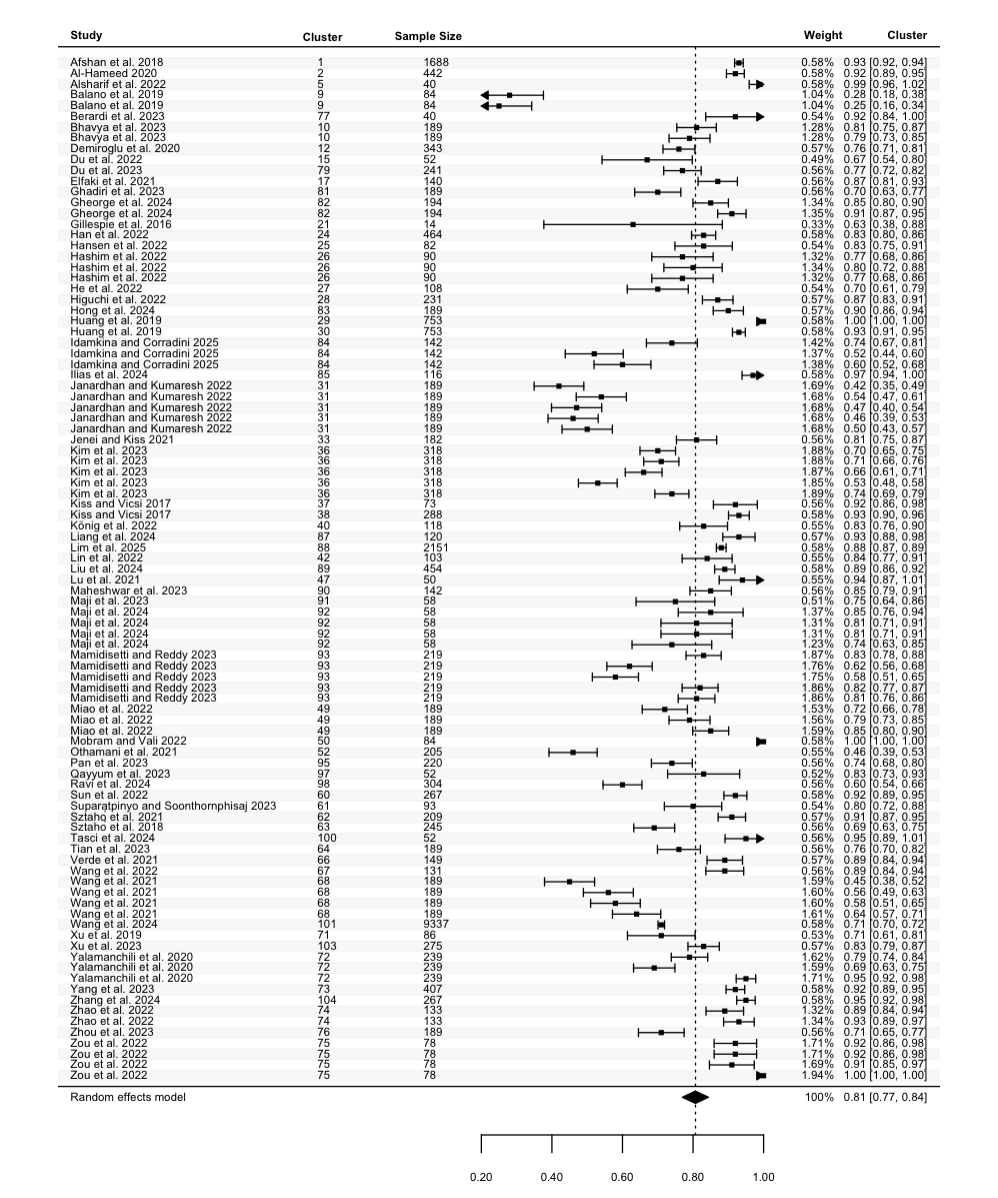
Three-level forest plot of the highest precision estimates. This forest plot illustrates the results of this 3-level meta-analysis based on 95 estimates of the highest precision, reported in 62 studies [34, 35, 37-40, 42-44, 46, 47, 49-51, 53-57, 59-62, 64-71, 74, 75, 77-85, 90-93, 95, 96, 99, 101, 104, 108-113, 116, 119, 125, 126, 129]. The solid squares represent point estimates of precision, with horizontal lines indicating the 95% CIs. The rhombus at the bottom represents the estimated pooled highest precision.

**Figure 9:**
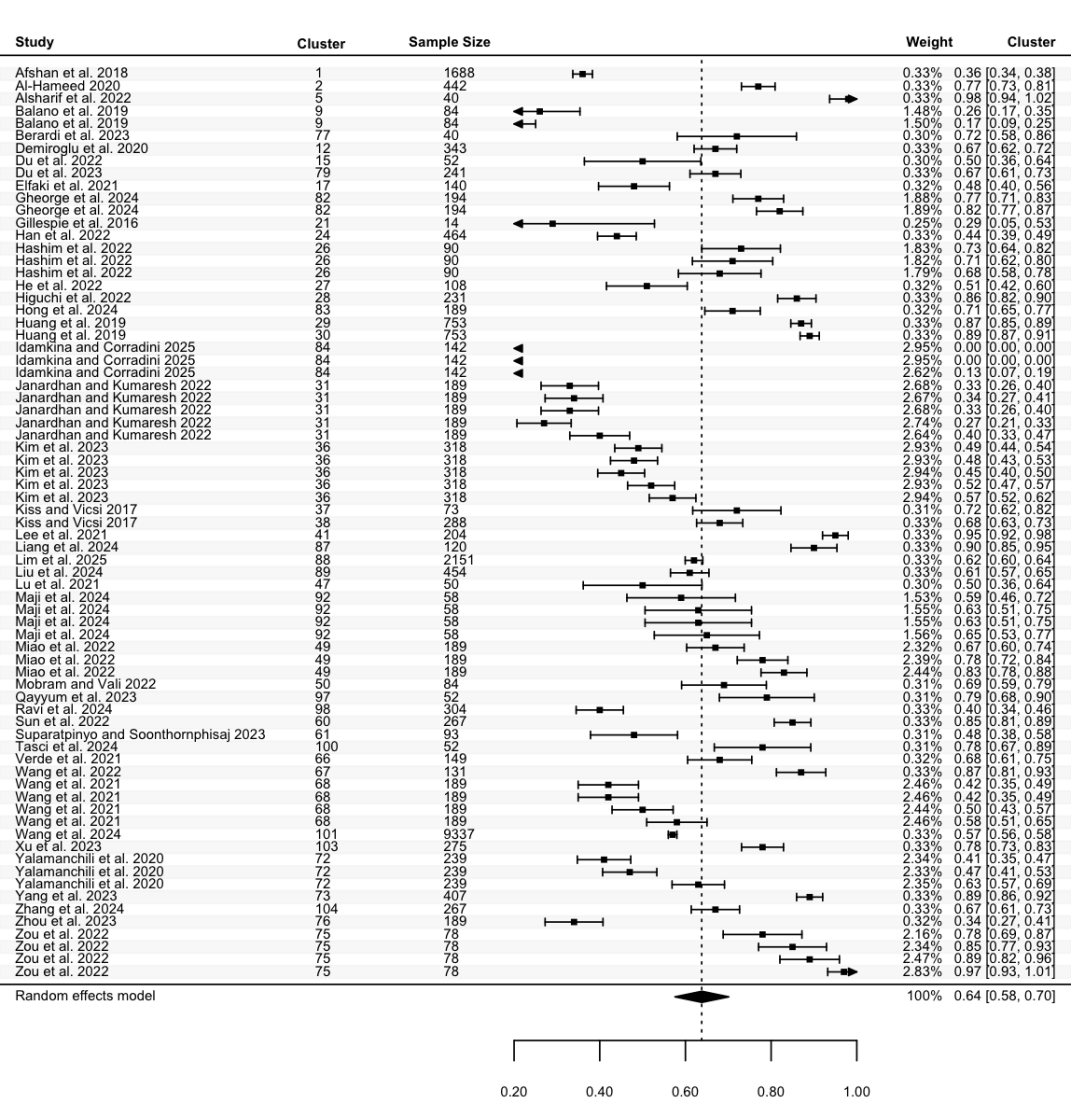
Three-level forest plot of the lowest precision estimates. This forest plot illustrates the results of this 3-level meta-analysis based on 73 estimates of the lowest precision, reported in 46 studies [34, 35, 37-40, 42-44, 47, 49, 50, 53, 59-61, 64, 65, 67-70, 74, 75, 77, 78, 81-84, 90-92, 96, 97, 101, 104, 108, 109, 111-113, 119, 125, 126, 129]. The solid squares represent point estimates of precision, with horizontal lines indicating the 95% CIs. The rhombus at the bottom represents the estimated pooled lowest precision.

Precision was reported in 62 studies, with 95 estimates for the highest precision (N=24,696). The highest precision in these studies ranged from 0.25 to 1.00, with a pooled mean of 0.81 (95% CI 0.77 to 0.84; Figure 8), and considerable heterogeneity (Cochran *P*<.001; *I*^2^=99.81%). For the lowest precision, 73 estimates from 46 studies (N=22,215) ranged between 0.00 and 0.98. The pooled mean was 0.64 (95% CI 0.58 to 0.70; Figure 9), with considerable heterogeneity (Cochran *P*<.001; *I*^2^=99.81%). No statistically significant differences in precision were identified across groups.

## Discussion

### Principal Findings

To the best of our knowledge, this is the first meta-analysis aimed at assessing the performance of ASA in detecting depression, including both machine learning and deep learning algorithms. Pooled data from 105 studies reveal that ASA demonstrates good, although not optimal, performance in classifying depression. Given that multiple studies conducted multiple experiments, we calculated the pooled mean of both the lowest and highest accuracy, sensitivity, specificity, and precision. Our results indicate that across studies, the pooled accuracy for depression detection was between 65% (ie, pooled mean of the lowest accuracy) and 81% (pooled mean of the highest accuracy), with comparable sensitivity (63%-84%) and specificity (60%-83%). This suggests that the ability of ASA to detect individuals with depression (sensitivity) was relatively consistent with its ability to identify those without depression (specificity). Additionally, pooled precision, which reflects the ability of ASA to correctly classify individuals who truly have depression among all those classified as depressed, ranged between 64% and 81%.

Our results revealed significant heterogeneity among studies. Meta-regression and subgroup analyses indicated a statistically significant difference between speech features, with Teager Energy Operator (TEO)–based features outperforming others in the pooled mean of the highest accuracy, sensitivity, and specificity. TEO-based features are based on the studies conducted by Teager and Teager [146], which demonstrated that airflow propagation in the vocal tract has a nonlinear character [146]. Moreover, convolutional neural network and deep neural network (DNN), in general, outperformed other algorithms in highest accuracy, while naïve bayes performed the worst. DNNs are often regarded as “black boxes” because their decision-making processes are not easily interpretable [147], which hinders clinicians’ trust and willingness to incorporate these models into the routine clinical workflow [148]. To foster trust in AI solutions for the detection of psychiatric conditions, it is important to implement a participatory approach, involving clinicians and other specialists in the model design process to create more interpretable algorithms that can better align with clinical needs and standards, thereby facilitating the incorporation of these potential solutions into clinical practice. Nevertheless, these results should be interpreted with caution as most studies in the current meta-analysis had a small sample size, which might have obscured further potential differences between AI algorithms. ASA performance is likely influenced by a complex interplay of factors that were not fully captured by the subgroup analyses. Beyond the measured variables, cultural and linguistic diversity, confounding factors such as comorbid conditions (eg, fatigue and anxiety), lifestyle influences, or medication effects, as well as differences in study protocols and dataset characteristics, may all contribute to the observed heterogeneity. These findings highlight the urgent need for standardized methodologies, diverse and inclusive datasets, and further research to understand and address these sources of heterogeneity.

### Comparison With Prior Work

In the context of AI diagnostic efficacy for depression detection, a recent meta-analysis conducted by Liu et al [30] assessing the diagnostic performance of deep learning algorithms in detecting depression through speech reported a superior accuracy of 0.87 (ie, highest accuracy). Notably, their analysis was restricted to peer-reviewed journal papers that included confusion matrices. In contrast, our review applied broader inclusion criteria, including conference papers, journal papers, and theses, and incorporated studies even if they reported a single performance metric. It is also important to note that their analyses were based on only 8 studies and exclusively focused on deep learning algorithms, whereas our meta-analysis included both machine learning and deep learning approaches. Similarly, Abd-Alrazaq et al [137] performed a meta-analysis focused on AI performance in detecting depression using wearable devices. Based on 38 studies, they reported superior accuracy (70%-93%), superior specificity (73%-93%), and slightly better sensitivity (61%-87%). Notably, their meta-analysis included studies using wearable devices that monitored a range of parameters, including physical activity, sleep patterns, and heart rate. Given the complex and multifaceted clinical profile of depression, integrating ASA into complex statistical models alongside other data sources, such as biological (eg, genetic, inflammatory, and neuroimaging data), psychological (eg, psychometric scales), and clinical variables, could significantly improve the accuracy and reliability of AI tools for depression detection. Another promising avenue is the use of facial expression analysis in combination with speech. However, this approach may raise additional ethical concerns, particularly regarding data privacy, consent, and the risk of algorithmic biases [149].

SVM was the most used algorithm to classify individuals with depression in the present meta-analysis. SVMs are highly regarded in machine learning for their robust ability to handle noisy, interrelated features and process datasets with high-dimensionality efficiently. Convolutional neural network and DNN, in general, were also commonly used. Our findings, in line with those of Liu et al [30], suggest that these architectures hold a significant potential in ASA. Therefore, we recommend that future research efforts go in this direction, particularly in combination with participatory methodologies that involve clinicians throughout the development process. This collaborative approach is essential to ensure the clinical relevance, feasibility, and eventual adoption of such algorithms in mental health care settings. Nonetheless, several challenges remain, such as the large sample sizes typically required to train deep learning models effectively and the considerable computational power needed to support their development and implementation.

DAIC-WOZ [150] was the most commonly used open-source dataset, which includes 189 clinical interviews conducted by Ellie, a virtual interviewer [150]. Although this dataset has facilitated significant advancements in the field, it presents several limitations that warrant attention. First, the participants were volunteers whose depressive symptoms were assessed using the Patient Health Questionnaire-8, rather than through formal clinical diagnosis. Second, the metadata associated with DAIC-WOZ and other datasets is sparse, leaving potential confounding factors unspecified [151]. Third, the dataset endures a significant class imbalance, with nondepressed participants outnumbering depressed ones by a ratio of approximately 4:1 [152]. Furthermore, the dataset exhibits a notable gender bias in depression prevalence, with females having a higher proportion of depressed patients (ratio of approximately 5:8 depressed to nondepressed) compared to males (ratio of about 2:7 depressed to nondepressed) [153]. This imbalance raises concerns about biased model training, as machine learning algorithms may overfit to the majority class [154], thereby reducing their ability to generalize effectively across diverse populations [155]. One promising direction for future research is to integrate principles of fair machine learning [156] as well as prior domain knowledge, such as gender-specific linguistic patterns or balanced sampling strategies, into the design of depression detection models, ensuring that algorithms account for gender and class imbalances while avoiding overfitting to unintended features [154].

In terms of speech features used, most studies included in this review focused exclusively on acoustic features, while only 15 studies incorporated lexical features. Linguistic studies have shown that depression also manifests in language use, specifically in semantic and syntactic patterns that reveal heightened self-focus and pervasive negative affect [157]. Recent advances in deep learning, particularly the development of transformer-based models, such as BERT (Bidirectional Encoder Representations From Transformers) and RoBERTa (Robustly Optimized Bidirectional Encoder Representations From Transformers Pretraining Approach), have shown significant promise in natural language and speech processing tasks, with some studies reporting accuracy rates of up to 98% in detecting depression [158-160]. BERT is a DNN model that generates bidirectional text representations while preserving contextual and semantic nuances, making it particularly suitable for analyzing language associated with mental health. However, most BERT-based studies to date have focused on text from social media platforms. Future research should explore the integration of such models with speech data to examine whether combining what is said (linguistic content) with how it is said (acoustic features) can offer a more comprehensive understanding of an individual’s mental health.

### Research and Practical Implications

This review highlights that ASA is an emerging technology with potential for detecting depression, though its readiness for clinical application is still limited. First, the ASA performance is not yet optimal, indicating room for improvement. Second, most included studies had small or modest sample sizes; for robust and reliable estimates, larger samples are required [161]. This is particularly critical as small training sets may lead to inflated accuracy estimates due to overfitting or random effects [162,163]. Third, more than half of the studies assessed had a high risk of bias in at least 1 domain. Fourth, there is a notable absence of large-scale studies that examine the generalizability of ASA across countries, settings, and depression severity levels. Moreover, the clinical and methodological variables that affect this generalization remain unclear. Fifth, more than half of the studies (52.4%) were conference papers, so more peer-reviewed, high-quality studies are warranted. Sixth, considering the diversity in speaker characteristics and individual speaking styles, it is imperative to conduct longitudinal studies to discern whether variations in speech patterns are indicative of depression symptoms or merely reflect inherent personal differences or related conditions such as increased anxiety or fatigue [14,29]. Such studies are essential for validating the diagnostic precision of ASA and ensuring its reliability across different individuals and contexts. Finally, most of the studies included in this review used self-reported questionnaires for depressive symptoms and applied commonly used cutoff scores to indicate the presence of depression. Only a small percentage used structured clinical interviews, such as the *DSM-IV* (*Diagnostic and Statistical Manual of Mental Disorders* [Fourth Edition]) criteria, the Composite International Diagnostic Interview, or the Mini-International Neuropsychiatric Interview, to establish a formal diagnosis. This methodological discrepancy has important implications for interpreting the findings. Self-report measures primarily capture symptom severity, and while useful for screening purposes, they do not equate to a clinical diagnosis of depression. As such, when relying on these instruments, the objective of the study shifts from predicting a clinical diagnosis to predicting questionnaire scores, which may not fully align with diagnostic criteria [103]. To determine whether ASA could serve as a viable method for diagnosing depression, future studies should incorporate clinical data based on a formal diagnosis of depression.

Early detection is paramount, particularly for conditions such as depression, where delays in diagnosis can significantly worsen outcomes [164,165]. Although ASA offers a potential cost-effective, clinically relevant solution that could make depression screening more feasible and accessible, these methods should still be considered complementary tools rather than a replacement for established diagnostic methods. Moreover, ensuring its equitable, effective, and sustainable deployment requires addressing critical ethical and implementation challenges. The Non-Adoption, Abandonment, Scale-Up, Spread, and Sustainability framework provides valuable guidance in this effort, offering an evidence-based approach for studying the nonadoption and abandonment of technologies by individuals and the challenges to scale-up, spread, and sustainability of such technologies in health care organizations and systems [166]. Specifically, future studies should explore how ASA interacts with organizational capacity, user adoption, decision-making processes, and broader policy contexts. For instance, disruptions to clinicians’ workflows [167] or gaps in organizational readiness [168] can significantly impede adoption. Furthermore, the explainability of AI algorithms and reproducibility of results are critical to building trust among health care professionals and patients [169]. The adoption of explainable artificial intelligence techniques, such as SHAP (Shapley Additive Explanations) [170] and Local Interpretable Model-Agnostic Explanations [171], can help elucidate the influence of individual acoustic features on AI model predictions. In our review, only Verde et al [172] used Local Interpretable Model-Agnostic Explanations to assess the relevance of acoustic features in the best-performing machine learning models, while Lin et al [79] applied SHAP. Notably, no other included studies used such explainable artificial intelligence techniques. Additionally, comprehensive frameworks for accountability and liability are necessary to clarify stakeholder responsibilities during the deployment process [169]. Finally, compliance with data protection laws must remain a priority to safeguard patient privacy [169]. For example, speech features could be extracted in a manner that prevents raw speech signal reconstruction [152,173], or processed and encrypted on local devices before being securely transmitted to servers for further analysis [174].

### Limitations

While this meta-analysis represents the first comprehensive review summarizing the performance of ASA in detecting depression, several limitations warrant mention. First, the analysis was confined to English-language papers, which may have excluded relevant research conducted in other languages. Second, we included only those studies that provide the performance metrics under examination. However, in cases where studies lacked complete information, we did not contact the authors to obtain the missing data. Third, the included studies encompassed diverse patient populations, which adds complexity to the interpretation of our findings due to the variability in demographic and clinical characteristics. For example, patients with chronic depression might exhibit different speech features compared to those experiencing a first episode of depression. This heterogeneity may influence the diagnostic performance of ASA, potentially resulting in greater accuracy in identifying more severe cases of depression while missing milder cases. However, the assessment of ASA performance by severity level was beyond the scope of the present meta-analysis, as we excluded studies specifically focused on the prediction of depression severity. Moreover, most included studies did not provide sufficient information on the severity of depression within their samples. Future research should therefore examine the performance of ASA across varying levels of depression severity, with particular emphasis on mild cases, to better evaluate its potential as a screening tool for early detection and intervention. Fourth, while we used a modified version of QUADAS-2 as proposed by Abd-Alrazaq et al [137], we acknowledge that this tool still has notable limitations when applied to AI-based research. QUADAS-2 was originally developed for conventional diagnostic accuracy studies and does not adequately address AI-specific sources of bias, such as algorithm and input data quality, real-world clinical applicability, and algorithm generalizability, among others [175]. Additionally, the “flow and timing” domain in QUADAS-2 is often weakly applied in this context, as speech data collection and depression assessments are often conducted simultaneously, limiting its relevance for evaluating temporal relationships or diagnostic latency. Recent initiatives have proposed comprehensive frameworks to support the development and evaluation of trustworthy AI in health care based on 6 guiding principles, including fairness, universality, traceability, usability, robustness, and explainability [176]. Building on these principles, a dedicated, standardized checklist tailored to AI-based diagnostic studies is urgently needed. Such a tool would enhance the rigor of bias assessment, improve reporting practices, and support the effective translation of AI solutions into clinical settings. Fifth, while we reported accuracy, sensitivity, specificity, and precision, these metrics alone may not fully capture model performance in the context of imbalanced datasets, which are common in depression detection. Metrics such as the *F*_1_-score, the area under the curve of the receiver operating characteristic curve, and the Matthews correlation coefficient are better suited for evaluating performance under class imbalance and should be considered in future research to provide a more comprehensive assessment of model effectiveness. Finally, our approach to pooling both the highest and lowest performance metrics across studies deviates from conventional meta-analytic practices and may limit direct comparability with prior work. However, we argue that this method offers specific advantages that justify its use in this context. Previous meta-analyses, such as that conducted by Liu et al [30], have typically reported only the highest performance, which may inadvertently overestimate model effectiveness, particularly in studies where multiple experiments are conducted and only the highest performance metrics are selected. By including both the highest and lowest reported outcomes, we sought to capture the inherent variability in AI-based research and mitigate potential reporting bias. This strategy is supported by prior work from Abd-Alrazaq et al [137], and aligns with ongoing calls for greater methodological transparency in AI research. For researchers seeking direct comparisons with more conventional meta-analyses, the highest pooled estimates in our analysis can serve as a benchmark.

### Conclusions

In sum, this study showed that ASA is a promising method for detecting depression, though its readiness for clinical application as a standalone tool remains limited. More peer-reviewed, high-quality studies are warranted to further advance this emerging field. At present, ASA should be considered as a complementary method, with potential application across various settings, including the general population, clinical field, primary care, or environments where stigma still presents a significant barrier to care. Future studies should focus on exploring how ASA can generalize across languages and cultures, and how it might be integrated into complex statistical models, alongside other data sources, to significantly improve depression detection within the stratified psychiatry framework. Additionally, successful clinical implementation of ASA will require addressing critical challenges, including ensuring data reproducibility, improving the explainability of AI algorithms, among other ethical and legal considerations.

## Appendix

Multimedia Appendix 1 PRISMA-DTA checklist.

Multimedia Appendix 2 Study characteristics, risk of bias, meta-regression and subgroup analyses, and search string.

## Abbreviations

AI: artificial intelligence
ASA: automatic speech analysis
BERT: Bidirectional Encoder Representations From Transformers
DAIC-WOZ: Distress Analysis Interview Corpus–Wizard of Oz
DNN: deep neural network
DSM-5-TR: Diagnostic and Statistical Manual of Mental Disorders (Fifth Edition, Text Revision)
DSM-IV: Diagnostic and Statistical Manual of Mental Disorders (Fourth Edition)
ICD-11: International Classification of Diseases, 11th Revision
PRISMA-DTA: Preferred Reporting Items for Systematic Reviews and Meta-Analyses—Extension for Diagnostic Test Accuracy
PROSPERO: International Prospective Register of Systematic Reviews
QUADAS-2: Quality Assessment of Studies of Diagnostic Accuracy-Revised
RoBERTa: Robustly Optimized Bidirectional Encoder Representations From Transformers Pretraining Approach
SHAP: Shapley Additive Explanations
SVM: support vector machine
TEO: Teager Energy Operator

## Data Availability

All data generated or analyzed in this study are available from the corresponding author upon reasonable request.
