# Supplementary material for: Performance of Automatic Speech Analysis in Detecting Depression: Systematic Review and Meta-Analysis
Source: JMIR Ment Health. 2025 Oct 22;12:e67802. doi: 10.2196/67802 (PMC12590051; doi:10.2196/67802)
Supplement: Multimedia Appendix 2 [file mental_v12i1e67802_app2.docx]

| **Table S1.** Characteristics of each included study. | | | | | | | |
| --- | --- | --- | --- | --- | --- | --- | --- |
| Study | Year | Publication type | Country | Sample size | Mean age | Female % | Depressed  participants % |
| Afshan et al. [119] | 2018 | Conference paper | USA | 1688 | NR | 100.0 | 47.0 |
| Al-Hameed [109] | 2020 | Thesis | UK | 442 | NR | NR | NR |
| Alghifari et al. [90] | 2019 | Conference paper | Malaysia | 189 | NR | NR | NR |
| Alghowinem et al. [135] | 2013 | Conference paper | Australia | 60 | NR | NR | 50.0 |
| Alsharif et al. [90] | 2022 | Conference paper | Saudi Arabia | 40 | NR | NR | 50.0 |
| Aradhana et al. [114] | 2019 | Conference paper | India | 189 | NR | NR | NR |
| Asgari et al. [133] | 2014 | Conference paper | USA | 148 | NR | 66.0 | 48.0 |
| Azam et al. [127] | 2016 | Conference paper | Malaysia | 33 | 28.9 | 52.0 | 39.0 |
| Balano et al. [113] | 2019 | Conference paper | Philippines | 84 | NR | NR | NR |
| Berardi et al. [67] | 2023 | Journal Article | Germany | 40 | 40.4 | 42.5 | 50.0 |
| Bhavya et al. [66] | 2023 | Conference paper | India | 189 | NR | NR | NR |
| Bn & Abdullah [89] | 2022 | Conference paper | USA | 100 | NR | 50.0 | 50.0 |
| Demiroglu et al. [108] | 2020 | Journal Article | Turkey | 343 | NR | NR | NR |
| Di et al. [103] | 2021 | Journal Article | China | 1808 | NR | NR | 43.0 |
| Di et al. [48] | 2024 | Journal Article | China | 7654 | 45.9 | 100.0 | 57.0 |
| Dong & Yang [102] | 2021 | Journal Article | China | NR | NR | NR | NR |
| Du et al. [64] | 2023 | Conference paper | China | 52 | NR | NR | 44.0 |
| Du et al. [65] | 2023 | Journal Article | China | 241 | NR | NR | 32.0 |
| Dumpala et al. [63] | 2023 | Journal Article | Canada | 733 | 42.7 | 65.8 | 24.0 |
| Egas-López et al. [88] | 2022 | Conference paper | Hungary | 222 | NR | 34.0 | 52.0 |
| Elfaki et al. [101] | 2021 | Conference paper | Malaysia | 140 | NR | NR | NR |
| Espinola et al. [100] | 2021 | Journal Article | Brazil | 33 | 38.6 | 67.0 | 67.0 |
| Ezzi et al. [87] | 2022 | Conference paper | Malaysia | 111 | NR | 100.0 | 38.0 |
| Fraser et al. [128] | 2016 | Conference paper | Canada | 130 | 71.5 | 69.2 | 50.0 |
| Ghadiri et al. [62] | 2023 | Conference paper | Iran | 189 | NR | NR | NR |
| Gheorge et al. [61] | 2023 | Conference paper | Romania | 194 | NR | 55.0 | NR |
| Gillespie et al. [129] | 2016 | Conference paper | USA | 14 | NR | 42.9 | 50.0 |
| Guo et al. [136] | 2013 | Conference paper | China | 60 | NR | NR | 55.0 |
| Hajduska-Dér et al. [86] | 2022 | Journal Article | Hungary | 218 | 42.3 | 66.0 | NR |
| Han et al. [60] | 2023 | Journal Article | China | 464 | NR | NR | NR |
| Hansen et al. [85] | 2022 | Journal Article | Denmark | 82 | 32.8 | 78.0 | NR |
| Hashim et al. [84] | 2022 | Journal Article | Malaysia | 90 | 26.6 | 73.0 | 48.0 |
| He et al. [83] | 2022 | Conference paper | China | 108 | 19.7 | 50.0 | 50.0 |
| Higuchi et al. [82] | 2022 | Journal Article | Japan | 231 | 36.6 | 72.0 | 44.0 |
| Hong et al. [47] | 2024 | Conference paper | Republic of Korea | 189 | NR | NR | 45.0 |
| Huang et al. [111] | 2019 | Journal Article | Australia | 753 | NR | NR | 21.0 |
| Huang et al. [112] | 2019 | Conference paper | Australia | 753 | NR | 58.0 | NR |
| Idamkina and Corradini [35] | 2025 | Conference paper | UK | 142 | NR | 44.4 | 30.0 |
| Ilias and Askounis [46] | 2024 | Conference paper | Greece | 116 | NR | NR | NR |
| Janardhan and Kumaresh [81] | 2022 | Journal Article | India | 189 | NR | NR | 30.0 |
| Jenei and Kiss [107] | 2020 | Conference paper | Hungary | 182 | 45.6 | NR | 50.0 |
| Jenei and Kiss [99] | 2021 | Journal Article | Hungary | 182 | NR | NR | 50.0 |
| Jiang et al. [120] | 2017 | Journal Article | China | 170 | NR | 61.0 | NR |
| Jiang et al. [118] | 2018 | Journal Article | China | 170 | NR | 61.0 | NR |
| Kim et al. [59] | 2023 | Journal Article | Republic of Korea | 318 | 37.9 | 71.0 | 48.0 |
| Kiss and Vicsi [125] | 2017 | Conference paper | Hungary | 73 | NR | 58.0 | NR |
| Kiss and Vicsi [126] | 2017 | Journal Article | Hungary | 288 | NR | 65.0 | 64.0 |
| Kiss et al. [98] | 2021 | Conference paper | Hungary | NR | NR | NR | NR |
| König et al. [80] | 2022 | Journal Article | France | 118 | 23.5 | 77.0 | 21.0 |
| Lee et al. [97] | 2021 | Journal Article | Republic of Korea | 204 | 71.7 | 70.0 | NR |
| Li and Li [45] | 2024 | Conference paper | China | 62 | 38.1 | 48.4 | 50.0 |
| Liang et al. [44] | 2024 | Journal Article | China | 120 | 19.0 | 79.2 | 53.0 |
| Lim et al. [34] | 2025 | Journal Article | Republic of Korea | 2151 | NR | NR | 39.0 |
| Lin et al. [79] | 2022 | Journal Article | China | 103 | 57.7 | 63.0 | 54.0 |
| Liu et al. [121] | 2017 | Conference paper | China | 184 | NR | 59.0 | 50.0 |
| Liu et al. [58] | 2023 | Journal Article | China | 536 | 36.4 | 51.0 | 58.0 |
| Liu et al. [43] | 2024 | Conference paper | UK | 454 | NR | NR | 49.0 |
| Long et al. [122] | 2017 | Conference paper | China | 74 | 36.3 | 50.0 | 50.0 |
| Lopez-Otero et al. [134] | 2014 | Conference paper | Spain | NR | NR | NR | NR |
| Lu et al. [96] | 2021 | Journal Article | China | 50 | 20.4 | 56.0 | 50.0 |
| Ma et al. [130] | 2016 | Conference paper | China | NR | NR | NR | NR |
| Maheshwar et al. [57] | 2023 | Conference paper | India | 142 | NR | 44.4 | 44.0 |
| Maji et al. [56] | 2023 | Conference paper | India | 58 | NR | 36.2 | 40.0 |
| Maji et al. [42] | 2024 | Conference paper | India | 58 | NR | 36.2 | 40.0 |
| Mamidisetti and Reddy [55] | 2023 | Conference paper | India | 219 | NR | NR | 69.0 |
| Menne et al. [41] | 2024 | Journal Article | Germany | 96 | 26.2 | 39.6 | 48.0 |
| Miao et al. [78] | 2022 | Journal Article | China | 189 | NR | NR | NR |
| Mobram and Vali [77] | 2022 | Journal Article | Iran | 84 | NR | NR | NR |
| Muzammel et al. [106] | 2020 | Journal Article | France | NR | NR | NR | NR |
| Othamani et al. [95] | 2021 | Conference paper | France | 205 | NR | NR | NR |
| Pan et al. [54] | 2023 | Journal Article | China | 220 | 34.1 | 50.0 | 48.0 |
| Parekh and Patil [123] | 2017 | Conference paper | India | 85 | NR | NR | NR |
| Patil and Wadhai [94] | 2021 | Conference paper | India | 129 | NR | NR | NR |
| Pérez-Toro et al. [76] | 2022 | Journal Article | Germany | 110 | 63.1 | 48.0 | 22.0 |
| Pratiwi et al. [33] | 2023 | Conference paper | Indonesia | 245 | NR | NR | 33.0 |
| Qayyum et al. [53] | 2023 | Journal Article | UK | 52 | 31.3 | 30.8 | 44.0 |
| Ravi et al. [40] | 2024 | Journal Article | USA | 304 | NR | NR | 24.0 |
| Shukla et al. [105] | 2020 | Conference paper | India | NR | NR | NR | NR |
| Srinivasan et al. [52] | 2023 | Conference paper | India | NR | NR | NR | NR |
| Stolar [131] | 2016 | Thesis | Australia | 63 | NR | NR | 46.0 |
| Stolar et al. [132] | 2015 | Conference paper | Australia | 63 | NR | 76.2 | 54.0 |
| Stolar et al. [117] | 2018 | Journal Article | Australia | 63 | NR | 76.2 | NR |
| Sun et al. [75] | 2022 | Conference paper | China | 267 | NR | NR | 31.0 |
| Suparatpinyo and Soonthornphisaj [68] | 2023 | Journal Article | Thailand | 93 | NR | 52.7 | 68.0 |
| Sztahó et al. [93] | 2021 | Conference paper | Hungary | 209 | NR | NR | 22.0 |
| Sztahó et al. [116] | 2018 | Conference paper | Hungary | 245 | 45.8 | 57.1 | 49.0 |
| Tasci [39] | 2024 | Journal Article | Turkey | 52 | 31.3 | 30.8 | 44.0 |
| Tian et al. [51] | 2023 | Journal Article | China | 189 | NR | NR | 30.0 |
| Tündik et al. [124] | 2017 | Conference paper | Hungary | 118 | NR | NR | 50.0 |
| Verde et al. [92] | 2021 | Conference paper | Italy | 149 | NR | 76.5 | 58.0 |
| Wang et al. [74] | 2022 | Conference paper | China | 131 | 30.4 | 72.5 | NR |
| Wang et al. [91] | 2021 | Journal Article | China | 189 | NR | NR | 50.0 |
| Wang et al. [38] | 2024 | Journal Article | USA | 9337 | NR | NR | 46.0 |
| Wu et al. [73] | 2022 | Conference paper | China | 189 | NR | NR | 35.0 |
| Xing et al. [72] | 2022 | Journal Article | China | 536 | 37.1 | 51.3 | 58.0 |
| Xing et al. [32] | 2025 | Journal Article | China | 84 | 31.5 | NR | NR |
| Xu et al. [110] | 2019 | Conference paper | Singapore | 86 | 37.8 | 46.5 | 52.0 |
| Xu et al. [50] | 2023 | Conference paper | China | 275 | NR | NR | 29.0 |
| Yalamanchili et al. [104] | 2020 | Conference paper | India | 239 | NR | NR | 27.0 |
| Yang et al. [49] | 2023 | Journal Article | China | 407 | NR | NR | 48.0 |
| Zhang et al. [37] | 2024 | Journal Article | China | 267 | NR | NR | NR |
| Zhao et al. [71] | 2022 | Journal Article | China | 133 | 35.6 | 67.7 | 53.0 |
| Zhou et al. [69] | 2023 | Journal Article | China | 189 | NR | NR | NR |
| Zhou et al. [36] | 2024 | Conference paper | Japan | 156 | 50.7 | 35.4 | 51.0 |
| Zou et al. [70] | 2022 | Journal Article | China | 78 | 28.4 | 67.9 | 33.0 |
| NR Not reported. | | | | | | | |

| **Table S2.** Characteristics of automatic speech analysis classifiers. | | | | | | | | | | | | |
| --- | --- | --- | --- | --- | --- | --- | --- | --- | --- | --- | --- | --- |
| Study | Speech features | | | | | | Dataset | Dataset language | Speech-eliciting tasks | AI algorithms | Ground truth assessment | Validation approach |
|  | Prosodic | Source | Format | Spectral | Lexical | TEO |  |  |  |  |  |  |
| Afshan et al. [119] | Yes | Yes | Yes | Yes | No | No | CONVERGE | Mandarin | Free speech: Dialogue: Interview recording | Logistic regression | CIDI (Chinese version) | Hold-out |
| Al-Hameed [109] | Yes | Yes | Yes | Yes | No | No | AVEC-2013, AVEC-2014, DAIC-WOZ | German and English | AVEC-2013: Contrained (counting and reading) and free speech (e.g., talking loud during task solving) AVEC-2014: Contrained (reading) and free speech (e.g., what was your best gift, and why?) DAIC-WOZ: Free speech: semi-structured interview | GB | BDI and PHQ | Hold-out |
| Alghifari et al. [90] | No | No | No | Yes | No | No | DAIC-WOZ | English | Free speech: semi-structured interview | DNN | PHQ-8 | Hold-out |
| Alghowinem et al. [135] | Yes | Yes | No | No | No | Yes | Hand-crafted dataset | English | Free speech: Dialogue (e.g., “Can you recall some recent good news you had and how did that make you feel?”) | SVM |  | Leave-5-utterances- per-subject-out |
| Alsharif et al. [90] | No | No | No | Yes | No | No | Hand-crafted dataset | Arabic | Free speech: Dialogue: Interview recording | CNN |  | Hold-out |
| Aradhana et al. [114] | No | No | No | No | No | No | DAIC-WOZ | English | Free speech: semi-structured interview | CNN | PHQ-8 | Hold-out |
| Asgari et al. [133] | Yes | Yes | No | Yes | Yes | No | Hand-crafted dataset | English | Free speech: Interactions with parents (e.g., event-planning interaction) | SVM | Living-in-family-environment (LIFE) coding system | 30-fold cross-validation |
| Azam et al. [127] | No | No | No | No | No | No | Hand-crafted dataset | Malay | Free speech: Dialogue: Interview recording | LDA, QDA | BDI-MALAY and BHS | LOOCV, Hold-out |
| Balano et al. [113] | Yes | No | No | Yes | No | No | Hand-crafted dataset |  | Free speech: Dialogue: Interview recording | SVM, DT | BDI-II | 10-fold cross-validation |
| Berardi et al. [67] | Yes | Yes | No | Yes | No | No | Hand-crafted dataset | German | Free speech: Picture description | SVM | SKID-I | 5-fold cross-validation |
| Bhavya et al. [66] | No | No | No | Yes | No | No | DAIC-WOZ | English | Free speech: semi-structured interview | LSTM, SVM | PHQ-8 | Hold-out |
| Bn & Abdullah [89] | No | No | No | Yes | No | No | DAIC-WOZ | English | Free speech: semi-structured interview | FL | PHQ-8 | 5-fold cross-validation |
| Demiroglu et al. [108] | Yes | Yes | No | Yes | Yes | No | Hand-crafted dataset, AVEC-2014, DAIC-WOZ | Turkish, German and English | Hand-craft: Free speech: Dialogue (e.g., Can you tell us a happy moment lately?) AVEC-2014: Contrained (reading) and free speech (e.g., what was your best gift, and why?) DAIC-WOZ: Free speech: semi-structured interview | SVM | BDI-II and PHQ-8 | Hold-out, leave-one-out (used only for the hand-crafted dataset, and AVEC-2014) |
| Di et al. [103] | No | No | No | Yes | No | No | Hand-crafted dataset | Chinese | Free speech: Dialogue: Interview recording | Logistic regression | Composite International Diagnostic Interview (Chinese version) | Hold-out, 5-fold cross-validation |
| Di et al. [48] | Yes | No | No | No | No | No | CONVERGE | Chinese | Free speech: Dialogue: Interview recording | Logistic regression, SVM, MLP, XGBoost | Composite International Diagnostic Interview (Chinese version) | Hold-out |
| Dong & Yang [102] | No | No | No | No | No | No | AVEC-2013, AVEC-2014 | German | AVEC-2013: Contrained (counting and reading) and free speech (e.g., talking loud during task solving) AVEC-2014: Contrained (reading) and free speech (e.g., what was your best gift, and why?) | MPL | BDI-II | Hold-out, 20-fold cross-validation |
| Du et al. [64] | No | No | No | Yes | No | No | DAIC-WOZ, MODMA | English, Chinese | DAIC-WOZ: Free speech: semi-structured interview; MODMA: Interview, repetition of words, and picture description | CNN-LSTM | DSM and PHQ-8 | Hold-out |
| Du et al. [65] | Yes | Yes | No | Yes | No | No | Hand-crafted dataset | Chinese | Contrained (reading) and free speech (interview and picture description) | MPL | PHQ-9 | Hold-out |
| Dumpala et al. [63] | Yes | Yes | No | Yes | No | No | DAIC-WOZ, Vocal Mind | English | Free speech: Interview | CNN, LSTM | PHQ-8, MADRS | 5-fold cross-validation |
| Egas-López et al. [88] | No | No | No | Yes | No | No | Hand-crafted dataset | Hungarian | Constrained: Reading | DNN | BDI-II | Nested |
| Elfaki et al. [101] | No | No | No | Yes | No | No | AVEC-2017 | English | NR | CNN |  | Hold-out |
| Espinola et al. [100] | No | No | No | Yes | No | No | Hand-crafted dataset | Portuguese | Free speech: Dialogue: Interview recording | Bayes Net, MPL, RF, SVM, Naive Bayes, Logistic regression | HAM-D | 10-fold cross-validation |
| Ezzi et al. [87] | No | No | No | Yes | No | No | Hand-crafted dataset | Malay | Contrained (reading) and free speech (answering to three questions) | RNN | BDI-II (Malay version), PHQ-9 | NR |
| Fraser et al. [128] | Yes | Yes | Yes | Yes | Yes | No | Pitt corpus in the DementiaBank database | English | Free speech: Picture description | Logistic regression | HAM-D | NR |
| Ghadiri et al. [62] | Yes | Yes | No | Yes | Yes | No | DAIC-WOZ | English | Free speech: Interview | Ensemble model | PHQ-8 | Hold-out |
| Gheorge et al. [61] | No | No | No | Yes | No | No | DAIC-WOZ, MODMA | English, Chinese | DAIC-WOZ: Free speech: semi-structured interview; MODMA: Interview, repetition of words, and picture description | MLP, 1D-CNN | PHQ-8, PHQ-9 | Hold-out, 10-fold cross-validation |
| Gillespie et al. [129] | Yes | Yes | Yes | Yes | No | No | Hand-crafted dataset | English | Constrained (Repetition) and free speech (picture description) | SVO-SVM | SADQ-10 | LOOCV |
| Guo et al. [136] | No | No | No | Yes | No | No | Hand-crafted dataset | Chinese | NR | GMM | DSM-IV | LOOCV |
| Hajduska-Dér et al. [86] | Yes | Yes | Yes | Yes | No | No | Hungarian Depressed Speech Database (DEPISDA) | Hungarian | Constrained: Reading | SVR | BDI, HAMD | Nested leave-one-out |
| Han et al. [60] | No | No | No | No | No | No | AVEC-2017, AVEC-2019 | English | NR | Speech-based spatial-temporal feature network (STFN) | PHQ-8 | Hold-out |
| Hansen et al. [85] | No | No | No | Yes | No | No | Training dataset: CREMA-D,RAVDESS, EMO-DB Testing dataset: Hand-crafted dataset | English, German, Danish | Free speech: Dialogue: Interview recording | GMM | HAM-D | Hold-out |
| Hashim et al. [84] | No | No | Yes | Yes | No | No | Hand-crafted dataset | Malay | Free speech - General question Constrained: Reading | RF, SVM, XGBoost | BDI-II (Malay version), PHQ-9 | Hold-out, 5-fold cross-validation |
| He et al. [83] | Yes | No | No | Yes | Yes | No | Hand-crafted dataset | Chinese | Free speech: Picture description, dialogue (e.g., How much sleep have you had recently?) | RNN | BDI-II | Hold-out |
| Higuchi et al. [82] | Yes | Yes | No | Yes | No | No | Hand-crafted dataset | Japanese | Constrained: Reading | Logistic regression | HDRS | Hold-out |
| Hong et al. [47] | No | No | No | Yes | No | No | DAIC-WOZ | English | Free speech: Interview | XGBoost | PHQ-8 | Hold-out |
| Huang et al. [111] | No | No | No | Yes | Yes | No | DAIC-WOZ, SH2 | English | DAIC-WOZ: Free speech: semi-structured interview SH2: Free speech | SVM | PHQ-8, PHQ-9 | Hold-out, 3-fold cross-validation |
| Huang et al. [112] | No | No | No | No | No | No | DAIC-WOZ, SH2 | English | DAIC-WOZ: Free speech: semi-structured interview SH2: Constrained - Sustained vowel, Diadochokinetic repetition, Reading, Free speech | SVM | PHQ-9 | Hold-out, 3-fold cross-validation |
| Idamkina and Corradini [35] | Yes | Yes | Yes | Yes | No | No | DAIC-WOZ | English | Free speech: Interview | MLP, SVM, XGBoost | PHQ-8 | Hold-out |
| Ilias and Askounis [46] | No | No | No | No | Yes | No | Androids corpus | Italian | Free speech - General question Constrained: Reading | DNN |  | 5-fold cross-validation |
| Janardhan and Kumaresh [81] | Yes | Yes | Yes | Yes | No | No | DAIC-WOZ | English | DAIC-WOZ: Free speech: semi-structured interview | GNB, SVC, kNN, Logistic regression, RF | PHQ-8 | Hold-out, 10-fold cross-validation |
| Jenei and Kiss [107] | No | No | Yes | Yes | No | No | Hungarian Depressed Speech Database (DEPISDA) | Hungarian | Constrained: Reading | CNN | BDI-II | LOOCV |
| Jenei and Kiss [99] | No | No | Yes | Yes | No | No | Hungarian Depressed Speech Database (DEPISDA) | Hungarian | Constrained: Reading | CNN | BDI-II | LOOCV |
| Jiang et al. [120] | Yes | Yes | No | Yes | No | No | Hand-crafted dataset | Chinese | Contrained (reading) and free speech (interview and picture description) | AdaBoost Decision Tree, Bagging decision tree, SVM, GMM, Logistic regression, RF, Ensemble model | PHQ-9 | 10-fold cross-validation |
| Jiang et al. [118] | Yes | Yes | No | Yes | No | No | Hand-crafted dataset | Chinese | Contrained (reading) and free speech (interview and picture description) | kNN, GMM, SVM, Ensemble model | PHQ-9 | 10-fold cross-validation |
| Kim et al. [59] | Yes | Yes | Yes | Yes | No | No | Hand-crafted dataset | Korean | Constrained - Sustained vowel, Counting, Reading | CNN, SVM, LDA, kNN, RF | Diagnostic and Statistical Manual of Mental Disorder | Hold-out, 10-fold cross-validation |
| Kiss and Vicsi [125] | Yes | Yes | Yes | Yes | No | No | AVEC-2013, Hand-crafted dataset | German, Hungaria, Italian | Constrained: Reading | SVM | BDI | Hold-out, leave-one-out |
| Kiss and Vicsi [126] | Yes | Yes | Yes | Yes | No | No | Hand-crafted dataset | Hungarian | Contrained (reading) and free speech (dialogue) | SVM |  | LOOCV |
| Kiss et al. [98] | Yes | Yes | Yes | Yes | No | No | Hand-crafted dataset | Hungarian | Constrained: Reading | SVM | BDI | 10-fold cross-validation |
| König et al. [80] | Yes | Yes | No | Yes | Yes | No | Hand-crafted dataset | German | Free speech: (e.g., Can you tell me in one minute about a positive/negative event in your life? | Logistic regression | Allgemeine Depressionsskala | LOOCV |
| Lee et al. [97] | Yes | Yes | Yes | Yes | No | No | Hand-crafted dataset | Korean | Free: Talk about your emotional and physical feelings during the past week Constrained: Reading | AdaBoost Decision Tree | Mini International Neuropsychiatric Interview (MINI-K) | Hold-out, 4-fold cross-validation |
| Li and Li [45] | Yes | No | No | Yes | No | No | Hand-crafted dataset | Chinese | Free speech: Interview, Picture description Constrained: Reading | Naive Bayes, RF, SVM, AdaBoost Decision Tree | DSM-IV | LOOCV |
| Liang et al. [44] | Yes | Yes | Yes | Yes | No | No | Hand-crafted dataset | Chinese | Constrained: Reading | DNN | DSM-IV | 5-fold cross-validation |
| Lim et al. [34] | Yes | No | No | Yes | Yes | No | DAIC-WOZ, EATD-Corpus, AIHUB | English, Chinese, Korean | Free speech: Interview, Picture description | DNN | PHQ-8, Self-rating Depression Scale, HAMD | Hold-out |
| Lin et al. [79] | No | No | No | Yes | No | No | Hand-crafted dataset | Chinese | Constrained: Reading | DNN | Mini International Neuropsychiatric Interview | Hold-out |
| Liu et al. [121] | No | No | No | No | No | No | DAIC-WOZ, Hand-crafted dataset | English, Chinese | DAIC-WOZ: Free speech: semi-structured interview Hand-crafted dataset: NR | CNN | PHQ-8, NR | Hold-out |
| Liu et al. [58] | Yes | Yes | Yes | Yes | No | No | Hand-crafted dataset | Chinese | Contrained (reading) and free speech (interview and picture description) | Ensemble model, SVM, kNN, Naive Bayes | Mini International Neuropsychiatric Interview | LOOCV |
| Liu et al. [43] | No | No | No | Yes | No | No | Hand-crafted dataset | Chinese | Free speech: Picture description, dialogue (e.g., How do you rate yourself?) | Ensemble model, SVM, RF | PHQ-9 | 10-fold cross-validation |
| Long et al. [122] | Yes | Yes | Yes | Yes | No | No | Hand-crafted dataset | Chinese | Contrained (reading) and free speech (interview and picture description) | SVM | BDI | 10-fold cross-validation |
| Lopez-Otero et al. [134] | Yes | Yes | Yes | Yes | No | No | AVEC-2013 DSC | English | NR | GMM | BDI | Hold-out, leave-one-out |
| Lu et al. [96] | No | No | No | Yes | No | No | Hand-crafted dataset | Chinese | Contrained (reading) and free speech (interview and picture description) | Attentional residual network | BDI-II | Hold-out |
| Ma et al. [130] | No | No | No | Yes | No | No | DAIC-WOZ | English | Free speech: semi-structured interview | DNN | PHQ-8 | Hold-out |
| Maheshwar et al. [57] | No | No | No | Yes | No | No | DAIC-WOZ | English | Free speech: Interview | SVM | PHQ-8 | Hold-out |
| Maji et al. [56] | Yes | Yes | No | Yes | No | No | Hand-crafted dataset | Bengali | Free speech: Picture description | SVM | DSM-V | 5-fold cross-validation |
| Maji et al. [42] | No | No | No | Yes | No | No | Hand-crafted dataset | Indic-Bengali | Free speech: Picture description | Logistic regression, DT, SVM, Naive Bayes | DSM-V | Hold-out |
| Mamidisetti and Reddy [55] | Yes | Yes | Yes | Yes | No | No | Hand-crafted dataset | English | Free speech - General question Constrained: Reading | Ensemble model, kNN, Naive Bayes, SVM, DT | HAM-D | Hold-out |
| Menne et al. [41] | Yes | Yes | Yes | Yes | Yes | No | Hand-crafted dataset | German | Free speech - General question | SVM | SCID-V | 10-fold cross-validation |
| Miao et al. [78] | Yes | Yes | Yes | Yes | No | No | DAIC-WOZ | English | Free speech: semi-structured interview | SVM, kNN, CNN | PHQ-8 | Hold-out |
| Mobram and Vali [77] | Yes | Yes | Yes | Yes | No | Yes | AVEC-2014 | German | Constrained: Reading | SVDA (Others) | BDI-II | Hold-out, 10-fold cross-validation |
| Muzammel et al. [106] | Yes | Yes | Yes | Yes | No | Yes | DAIC-WOZ | English | Free speech: semi-structured interview | CNN | PHQ-8 | Hold-out |
| Othamani et al. [95] | Yes | Yes | Yes | Yes | No | No | RECOLA + DAIC-WOZ | English | NR | CNN | PHQ-8 | Hold-out, leave-one-out |
| Pan et al. [54] | No | No | No | Yes | No | No | Hand-crafted dataset | Chinese | Free speech - General question Constrained: Reading | Logistic regression | DSM-IV | Hold-out, 5-fold cross-validation |
| Parekh and Patil [123] | Yes | Yes | Yes | Yes | No | Yes | DAIC-WOZ | English | Free speech: semi-structured interview | SVM, GMM, SVM+GMM | PHQ-8 | NR |
| Patil and Wadhai [94] | Yes | Yes | No | Yes | No | No | Hand-crafted dataset |  | Free speech: Interview | GMM, RF, SVM, Naive Bayes, SVM+GMM | PHQ 9, BDI | Hold-out |
| Pérez-Toro et al. [76] | Yes | Yes | No | Yes | Yes | No | Hand-crafted dataset | Spanish | Free speech: Interview | GMM | MDS-UPDRS | Leave-one-out |
| Pratiwi et al. [33] | No | No | No | No | No | No | DAIC-WOZ, MODMA | English, Chinese | DAIC-WOZ: Free speech: semi-structured interview; MODMA: Interview, repetition of words, and picture description | DNN | PHQ-8, PHQ-9 | Hold-out |
| Qayyum et al. [53] | No | No | No | No | No | No | MODMA | Chinese | MODMA: Interview, repetition of words, and picture description | CNN | PHQ-9 | NR |
| Ravi et al. [40] | Yes | Yes | Yes | Yes | No | No | DAIC-WOZ, EATD-Corpus | English, Chinese | Free speech: Interview, Picture description | CNN-LSTM | PHQ-8, Self-rating Depression Scale | Hold-out |
| Shukla et al. [105] | No | No | No | Yes | No | No | RAVDESS | English | NR | MPL |  | NR |
| Srinivasan et al. [52] | Yes | Yes | Yes | Yes | No | Yes | AVEC-2017 | English | NR | CNN | PHQ-8 | Hold-out |
| Stolar [131] | Yes | Yes | Yes | Yes | No | Yes | Oregan Research Intitute Database (ORI-DB) | English | Free speech: Interactions with parents (e.g., event-planning interaction) | SVM | DSM-IV | Hold-out |
| Stolar et al. [132] | No | No | No | Yes | No | Yes | Hand-crafted dataset | English | Free speech: Interactions with parents (e.g., event-planning interaction) | GMM, SVM | DSM-IV | Hold-out, leave-one-out |
| Stolar et al. [117] | No | No | Yes | Yes | No | Yes | Oregan Research Intitute Database (ORI-DB) | English | Free speech: Interactions with parents (e.g., event-planning interaction) | SVM | DSM-IV | Hold-out, 3-fold cross-validation |
| Sun et al. [75] | No | No | No | Yes | No | No | DAIC-WOZ, Hand-crafted dataset | English, Chinese | Free speech: semi-structured interview | ANN | PHQ-8 | 5-fold cross-validation |
| Suparatpinyo and Soonthornphisaj [68] | No | No | No | Yes | No | No | Hand-crafted dataset | Thai | Free speech: (e.g., questions about happiness in life) | ResNet (other) | PHQ-9 | Hold-out |
| Sztahó et al. [93] | No | No | No | Yes | No | No | Hand-crafted dataset | Hungarian | Constrained: Reading | LSTM | BDI-II | 10-fold cross-validation |
| Sztahó et al. [116] | Yes | No | Yes | Yes | No | No | Hand-crafted dataset | Hungarian | Constrained: Reading | SVM | BDI-II | 10-fold cross-validation |
| Tasci [39] | No | No | No | No | No | No | MODMA | Chinese | MODMA: Interview, repetition of words, and picture description | kNN | PHQ-9 | 10-fold cross-validation |
| Tian et al. [51] | Yes | Yes | Yes | Yes | No | Yes | DAIC-WOZ | English | Free speech: semi-structured interview | CNN | PHQ-8 | Hold-out |
| Tündik et al. [124] | Yes | Yes | No | Yes | No | No | Hand-crafted dataset | Hungarian | Constrained: Reading | DNN, SVM |  | Leave-one-out |
| Verde et al. [92] | No | Yes | No | Yes | No | No | Hand-crafted dataset | Italian | Free speech: General question (e.g., Talk about your daily activities accomplished during the last week) | SVM | BDI-II | 10-fold cross-validation |
| Wang et al. [74] | No | No | No | No | No | No | DAIC-WOZ, Converge 1, Converge 2 | English, Mandarin | Semi-structured interview | DNN | PHQ-8, Composite International Diagnostic Interview (Chinese version) | Hold-out |
| Wang et al. [91] | No | No | No | Yes | No | No | DAIC-WOZ | English | Free speech: semi-structured interview | 3D-CBHGA, SVM, RF, 1D-CBBG | PHQ-8 | Hold-out |
| Wang et al. [38] | No | No | No | Yes | Yes | No | Hand-crafted dataset | Mandarin | Free speech: depression-related questions (e..g., do you have insomnia recently?) | ANN | HAMD | 5-fold cross-validation |
| Wu et al. [73] | Yes | Yes | Yes | Yes | No | No | DAIC-WOZ | English | Free speech: semi-structured interview | SVM | PHQ-8 | Hold-out, 10-fold cross-validation |
| Xing et al. [72] | Yes | Yes | Yes | Yes | No | No | Hand-crafted dataset | Mandarin | Free speech: Picture description, dialogue (e.g., How do you evaluate yourself?) | RF, SVM | PHQ-9 | 10-fold cross-validation |
| Xing et al. [32] | Yes | Yes | Yes | Yes | No | No | AVEC-2014 | English | Free speech - General question Constrained: Reading | HMTL-IMHAFF | BDI-II | Hold-out |
| Xu et al. [110] | Yes | Yes | Yes | Yes | Yes | No | E-DAIC | English | Free speech: Interview | CNN–BiLSTM | PHQ-8 | Hold-out |
| Xu et al. [50] | Yes | Yes | Yes | Yes | Yes | No | Hand-crafted dataset | Singapore English | Free speech: Semi-structured interview | Ensemble model |  | LOOCV |
| Yalamanchili et al. [104] | Yes | Yes | No | Yes | No | No | DAIC-WOZ, Hand-crafted dataset | English | DAIC-WOZ: Free speech: semi-structured interview Hand-crafted dataset: Constrained: Reading | Logistic regression, RF, SVM | PHQ-8 | Hold-out |
| Yang et al. [49] | No | No | No | Yes | No | No | DAIC-WOZ, Hand-crafted dataset | English, Mandarin | DAIC-WOZ: Free speech: semi-structured interview Hand-crafted dataset: Constrained: Reading | DALF | PHQ-8, HAMD-17 | Hold-out, 5-fold cross-validation |
| Zhang et al. [37] | Yes | Yes | No | Yes | No | No | DAIC, CMDC | English, Chinese | Free speech: Interview | LSTM + Self-attention | PHQ-8 | Hold-out |
| Zhao et al. [71] | Yes | Yes | No | Yes | No | No | Hand-crafted dataset | Mandarin | Constrained: Reading | Logistic regression, SVM | HAMD-17, PHQ-9 | Hold-out, 10-fold cross-validation |
| Zhou et al. [69] | No | No | No | No | No | No | Hand-crafted dataset | Japanese | Constrained: Reading | SVM | PHQ-9 | Hold-out |
| Zhou et al. [36] | Yes | Yes | Yes | Yes | No | No | DAIC-WOZ | English | Free speech: semi-structured interview | CNN | PHQ-8 | Hold-out |
| Zou et al. [70] | Yes | Yes | Yes | Yes | Yes | No | Hand-crafted dataset | Chinese | Free speech: Interview | Bi-LSTM, SVM, Logistic regression, Naive Bayes | HAMD-17, PHQ-9 | Hold-out, 5-fold cross-validation |
| 1D-CBBG One-Dimensional Convolutional filter Bank and Bidirectional Gated Recurrent Unit, 3D-CBHGA Three-Dimensional Convolutional filter bank with Highway Networks and Bidirectional Gated Recurrent Unit with an Attention mechanism, AVEC Audio/Visual Emotion Challenge, BDI Beck Depression Inventory, CIDI Composite International Diagnostic Interview, Bi-LSTM Bidirectional Long Short-Term Memory, BHS Beck Hopelessness Scale, CMDC Chinese Multimodal Depression Corpus, CREMA-D Crowd Sourced Emotional Multimodal Actors Dataset, CNN Convolutional Neural Network, CONVERGE China, Oxford and Virginia Commonwealth University Experimental Research on Genetic Epidemiology, DAIC-WOZ Distress Analysis Interview Corpus Wizard-of-Oz, DEPISDA Hungarian Depressed Speech Database, DNN Deep Neural Network, DSM Diagnostic and Statistical Manual of Mental Disorders, DSM-IV Diagnostic and Statistical Manual of Mental Disorders, Fourth Edition, DT Decision Tree, E-DAIC Extended Distress Analysis Interview Corpus**,** EMO-DB Emotional Speech database, FL Federated Learning, GB Gradient Boosting, GMM Gaussian Mixture Model, GNB Guassian Naïve Bayes, HAM-D Hamilton Depression Rating Scale, HMTL-IMHAFF Hierarchical Multi-task Learning based on Interactive Multi-head Attention Feature Fusion, kNN k-Nearest Neighbors, LDA Linear Discriminant Analysis, LIFE Living-in-family-environment, LOOCV Leave-one-out cross-validation, MDS-UPDRS Movement Disorder Society - Unified Parkinson's Disease Rating Scale, MINI Mini-International Neuropsychiatric Interview, MLP Multi-layer Perceptron, MODMA Multi-modal Open Dataset for Mental-disorder Analysis NR Not Reported, ORI-DB Oregon Research Institute Database, PHQ Patient Health Questionnaire, QDA Quadratic Discriminant Analysis, RAVDESS Ryerson Audio-Visual Database of Emotional Speech and Song, RF Random Forest, RNN Recurrent Neural Network, SADQ-10 Stroke Aphasia Depression Questionnaire-10, STFN Speech-based spatial-temporal feature network, SVM Support Vector Machine, SVR Support Vector Regression, TEO Teager Energy Operator, XGBoost Extreme gradient boosting. | | | | | | | | | | | | |

**Table S3.** Reviewers’ judgments about each domain in “risk of bias” and "applicability concerns" for each included study.

| Study | Risk of bias | | | |  | Applicability concerns | | |
| --- | --- | --- | --- | --- | --- | --- | --- | --- |
|  | D1 | D2 | D3 | D4 |  | D1 | D2 | D3 |
| Afshan et al. [119] | ✗ | ✓ | ✓ | ? |  | ✗ | ✓ | ✓ |
| Al-Hameed [109] | ✓ | ✓ | ✓ | ✓ |  | ✓ | ✓ | ✓ |
| Alghifari et al. [90] | ? | ✓ | ✓ | ? |  | ✓ | ✓ | ? |
| Alghowinem et al. [135] | ✗ | ✓ | ✗ | ✗ |  | ✓ | ✓ | ✓ |
| Alsharif et al. [90] | ✓ | ✓ | ? | ? |  | ✓ | ✓ | ✓ |
| Aradhana et al. [114] | ✓ | ✓ | ✓ | ✗ |  | ✓ | ✓ | ? |
| Asgari et al. [133] | ✓ | ✓ | ? | ? |  | ✓ | ✓ | ✓ |
| Azam et al. [127] | ✗ | ✓ | ✓ | ? |  | ✓ | ✓ | ✓ |
| Balano et al. [113] | ✗ | ✓ | ✓ | ? |  | ✓ | ✓ | ✓ |
| Berardi et al. [67] | ✗ | ✓ | ✓ | ✓ |  | ✓ | ✓ | ✓ |
| Bhavya et al. [66] | ✓ | ✓ | ✓ | ✓ |  | ✓ | ✓ | ✓ |
| Bn & Abdullah [89] | ✓ | ✓ | ✓ | ? |  | ✓ | ✓ | ✓ |
| Demiroglu et al. [108] | ✓ | ✓ | ✓ | ? |  | ✓ | ✓ | ✓ |
| Di et al. [103] | ✓ | ✓ | ✓ | ✓ |  | ✓ | ✓ | ✓ |
| Di et al. [48] | ✓ | ✓ | ✓ | ✓ |  | ✓ | ✓ | ✓ |
| Dong & Yang [102] | ? | ✓ | ✓ | ✓ |  | ✓ | ✓ | ✓ |
| Du et al. [64] | ✗ | ✓ | ✓ | ✓ |  | ✓ | ✓ | ✓ |
| Du et al. [65] | ✓ | ✓ | ✓ | ✓ |  | ✓ | ✓ | ✓ |
| Dumpala et al. [63] | ✓ | ✓ | ✓ | ✓ |  | ✓ | ✓ | ✓ |
| Egas-López et al. [88] | ✓ | ✓ | ✓ | ✓ |  | ✓ | ✓ | ✓ |
| Elfaki et al. [101] | ✓ | ✓ | ? | ? |  | ✓ | ✓ | ? |
| Espinola et al. [100] | ✗ | ✓ | ? | ? |  | ✓ | ✓ | ✓ |
| Ezzi et al. [87] | ? | ✓ | ? | ✗ |  | ✓ | ✓ | ✓ |
| Fraser et al. [128] | ✓ | ✓ | ✓ | ? |  | ✓ | ✓ | ✓ |
| Ghadiri et al. [62] | ✓ | ✓ | ✓ | ✓ |  | ✓ | ✓ | ✓ |
| Gheorge et al. [61] | ✓ | ✓ | ✓ | ✓ |  | ✓ | ✓ | ✓ |
| Gillespie et al. [129] | ✗ | ✓ | ✓ | ? |  | ✓ | ✓ | ✓ |
| Guo et al. [136] | ✗ | ✓ | ✓ | ✗ |  | ✓ | ✓ | ✓ |
| Hajduska-Dér et al. [86] | ✓ | ✓ | ? | ✓ |  | ✓ | ✓ | ✓ |
| Han et al. [60] | ✓ | ✓ | ✓ | ✓ |  | ✓ | ✓ | ✓ |
| Hansen et al. [85] | ✗ | ✓ | ✓ | ✓ |  | ✓ | ✓ | ✓ |
| Hashim et al. [84] | ✗ | ✓ | ✓ | ? |  | ✓ | ✓ | ✓ |
| He et al. [83] | ✓ | ✓ | ? | ? |  | ✓ | ✓ | ✓ |
| Higuchi et al. [82] | ✓ | ✓ | ? | ✓ |  | ✓ | ✓ | ✓ |
| Hong et al. [47] | ✓ | ✓ | ✓ | ✓ |  | ✓ | ✓ | ✓ |
| Huang et al. [111] | ? | ✓ | ✓ | ? |  | ✓ | ✓ | ✓ |
| Huang et al. [112] | ✓ | ✓ | ✓ | ? |  | ✓ | ✓ | ✓ |
| Idamkina and Corradini [35] | ✓ | ✓ | ✓ | ✓ |  | ✓ | ✓ | ✓ |
| Ilias and Askounis [46] | ? | ✓ | ✓ | ✓ |  | ? | ? | ✓ |
| Janardhan and Kumaresh [81] | ✓ | ✓ | ✓ | ? |  | ✓ | ✓ | ✓ |
| Jenei and Kiss [107] | ✓ | ✓ | ✓ | ✗ |  | ✓ | ✓ | ✓ |
| Jenei and Kiss [99] | ✓ | ✓ | ✓ | ? |  | ✓ | ✓ | ✓ |
| Jiang et al. [120] | ✓ | ✓ | ✓ | ✓ |  | ✓ | ✓ | ✓ |
| Jiang et al. [118] | ✓ | ✓ | ✓ | ? |  | ✓ | ✓ | ✓ |
| Kim et al. [59] | ✓ | ✓ | ✓ | ✓ |  | ✓ | ✓ | ✓ |
| Kiss and Vicsi [125] | ✗ | ✓ | ? | ? |  | ✓ | ✓ | ? |
| Kiss and Vicsi [126] | ? | ✓ | ? | ? |  | ✓ | ✓ | ✓ |
| Kiss et al. [98] | ✗ | ✗ | ? | ? |  | ? | ✓ | ✓ |
| König et al. [80] | ✓ | ✓ | ✓ | ✓ |  | ✓ | ✓ | ✓ |
| Lee et al. [97] | ✓ | ✓ | ✓ | ✗ |  | ✓ | ✓ | ✓ |
| Li and Li [45] | ✗ | ✓ | ✓ | ✓ |  | ✓ | ✓ | ✓ |
| Liang et al. [44] | ✓ | ✓ | ✓ | ✓ |  | ✓ | ✓ | ✓ |
| Lim et al. [34] | ✓ | ✓ | ✗ | ✓ |  | ✓ | ✓ | ✓ |
| Lin et al. [79] | ✓ | ✓ | ✓ | ✓ |  | ✓ | ✓ | ✓ |
| Liu et al. [121] | ✓ | ✓ | ✓ | ✗ |  | ✓ | ✓ | ✓ |
| Liu et al. [58] | ✓ | ✓ | ✓ | ✗ |  | ✓ | ✓ | ✓ |
| Liu et al. [43] | ✓ | ✓ | ✓ | ✓ |  | ✓ | ✓ | ✓ |
| Long et al. [122] | ✗ | ✓ | ✓ | ✗ |  | ✓ | ✓ | ✓ |
| Lopez-Otero et al. [134] | ? | ✓ | ✓ | ✗ |  | ✓ | ✓ | ✓ |
| Lu et al. [96] | ✗ | ✓ | ✓ | ? |  | ✓ | ✓ | ✓ |
| Ma et al. [130] | ? | ✓ | ✓ | ? |  | ✓ | ✓ | ✓ |
| Maheshwar et al. [57] | ✓ | ✓ | ✓ | ✓ |  | ✓ | ✓ | ✓ |
| Maji et al. [56] | ✗ | ✓ | ✓ | ✓ |  | ✓ | ✓ | ✓ |
| Maji et al. [42] | ✗ | ✓ | ✓ | ✓ |  | ✓ | ✓ | ✓ |
| Mamidisetti and Reddy [55] | ✗ | ✓ | ✓ | ✓ |  | ✓ | ✓ | ✓ |
| Menne et al. [41] | ✗ | ✓ | ✓ | ✓ |  | ✓ | ✓ | ✓ |
| Miao et al. [78] | ✓ | ✓ | ✓ | ? |  | ✓ | ✓ | ✓ |
| Mobram and Vali [77] | ✗ | ✓ | ✓ | ? |  | ✓ | ✓ | ✓ |
| Muzammel et al. [106] | ✓ | ✓ | ✓ | ✓ |  | ✓ | ✓ | ✓ |
| Othamani et al. [95] | ✓ | ✓ | ✓ | ✓ |  | ✓ | ✓ | ✓ |
| Pan et al. [54] | ✓ | ✓ | ✓ | ✓ |  | ✓ | ✓ | ✓ |
| Parekh and Patil [123] | ✗ | ✓ | ✓ | ? |  | ✓ | ✓ | ✓ |
| Patil and Wadhai [94] | ? | ✓ | ✓ | ✗ |  | ✓ | ✓ | ✓ |
| Pérez-Toro et al. [76] | ✗ | ✓ | ✓ | ? |  | ✗ | ✓ | ✓ |
| Pratiwi et al. [33] | ✓ | ✗ | ✓ | ✗ |  | ✓ | ✗ | ✓ |
| Qayyum et al. [53] | ✗ | ✗ | ✓ | ✗ |  | ✓ | ? | ✓ |
| Ravi et al. [40] | ✓ | ✓ | ✗ | ✓ |  | ✓ | ✓ | ✓ |
| Shukla et al. [105] | ? | ✓ | ? | ✗ |  | ✓ | ✓ | ? |
| Srinivasan et al. [52] | ? | ✓ | ✓ | ✓ |  | ? | ✓ | ✓ |
| Stolar [131] | ✗ | ✓ | ✓ | ? |  | ✓ | ✓ | ✓ |
| Stolar et al. [132] | ✗ | ✓ | ✓ | ? |  | ✓ | ✓ | ✓ |
| Stolar et al. [117] | ✗ | ✓ | ✓ | ? |  | ✓ | ✓ | ✓ |
| Sun et al. [75] | ✗ | ✓ | ✓ | ✓ |  | ✓ | ✓ | ✓ |
| Suparatpinyo and Soonthornphisaj [68] | ✗ | ✓ | ✓ | ? |  | ✓ | ✓ | ✓ |
| Sztahó et al. [93] | ✓ | ? | ✓ | ? |  | ✓ | ✓ | ✓ |
| Sztahó et al. [116] | ✗ | ? | ✓ | ? |  | ? | ✓ | ✓ |
| Tasci [39] | ✗ | ✓ | ✓ | ✓ |  | ✓ | ✓ | ✓ |
| Tian et al. [51] | ✗ | ✓ | ✓ | ? |  | ✓ | ✓ | ✓ |
| Tündik et al. [124] | ? | ✓ | ? | ✗ |  | ✓ | ✓ | ? |
| Verde et al. [92] | ✓ | ✓ | ✓ | ? |  | ✓ | ✓ | ✓ |
| Wang et al. [74] | ✓ | ✓ | ✓ | ? |  | ✓ | ✓ | ✓ |
| Wang et al. [91] | ✓ | ✓ | ✓ | ? |  | ✓ | ✓ | ✓ |
| Wang et al. [38] | ✓ | ✗ | ✓ | ✓ |  | ✓ | ? | ✓ |
| Wu et al. [73] | ✓ | ✓ | ✓ | ? |  | ✓ | ✓ | ✓ |
| Xing et al. [72] | ✓ | ✓ | ✓ | ✗ |  | ✓ | ✓ | ✓ |
| Xing et al. [32] | ✗ | ✓ | ✓ | ✗ |  | ? | ✓ | ✓ |
| Xu et al. [110] | ✗ | ✓ | ? | ✓ |  | ? | ✓ | ? |
| Xu et al. [50] | ✓ | ✓ | ✓ | ✓ |  | ✓ | ✓ | ✓ |
| Yalamanchili et al. [104] | ✗ | ✓ | ✓ | ? |  | ✓ | ✓ | ✓ |
| Yang et al. [49] | ? | ✓ | ✓ | ? |  | ✓ | ✓ | ✓ |
| Zhang et al. [37] | ✓ | ✓ | ✓ | ✓ |  | ✓ | ✓ | ✓ |
| Zhao et al. [71] | ✓ | ✓ | ✓ | ✓ |  | ✓ | ✓ | ✓ |
| Zhou et al. [69] | ? | ✓ | ✓ | ? |  | ✓ | ✓ | ✓ |
| Zhou et al. [36] | ✓ | ✓ | ✓ | ✓ |  | ✓ | ✓ | ✓ |
| Zou et al. [70] | ✗ | ✓ | ✓ | ✓ |  | ✓ | ✓ | ✓ |

D1 = Participant; D2 = Index test; D3 = Reference standard; D4 = Analysis.
✓ indicates low risk; ✗ indicates high risk; ? indicates unclear risk.

**Figure S1.** Results of the overall risk of bias and applicability concerns assessment. The upper panel shows the risk of bias across four domains, including Participant, Index Test, Reference Standard, and Analysis, categorized as low (green), unclear (yellow), and high (red). The lower panel presents the overall applicability concerns across three domains, including Participant, Index Test, and Reference Standard. Green indicates low concerns, yellow represents unclear concerns, and red represents high concerns.

**Table S4.** Estimated pooled mean of highest accuracy by several factors.

| Groups | Number of studies | | Sample size | Accuracy (%) | Pooled mean accuracy | | | Heterogeneity measures | | | | | Test for subgroups differences |
| --- | --- | --- | --- | --- | --- | --- | --- | --- | --- | --- | --- | --- | --- |
|  | Total N | | Total N | Range | Mean (%) (95% CI) | | | Tau^2^ | Q (p-value) | | I^2^ (%) | | F (p-value) |
| Type of publication | | | | | | | | | | | | | |
| Journal Article | 76 | 48380 | | 0.61-0.98 | | 0.82 (0.79-0.84) | | 0.003 | | 1051.65 (<.001) | | 95.92 | 1.97 (.14) |
| Conference paper | 70 | 12038 | | 0.29-0.99 | | 0.80 (0.76-0.84) | |  |  | 1495.97 (<.001) | | 95.95 |  |
| Speech features |  | | | | | |  | | | | | | |
| Spectral features | 131 | | 57332 | 0.29-0.99 | 0.81 (0.78-0.83) | | | 0.003 | 3204.76 (<.001) | | 96.98 | | 2.24 (.04) |
| Prosodic features | 90 | | 50204 | 0.29-0.99 | 0.79 (0.76-0.82) | | |  | 2463.47 (<.001) | | 97.78 | |  |
| Source features | 80 | | 47233 | 0.62-0.99 | 0.80 (0.77-0.83) | | |  | 2249.44 (<.001) | | 96.89 | |  |
| Format features | 55 | | 42126 | 0.62-0.99 | 0.81 (0.77-0.84) | | |  | 1866.05 (<.001) | | 97.63 | |  |
| Lexical features | 14 | | 4651 | 0.66-0.95 | 0.81 (0.75-0.88) | | |  | 161.38 (<.001) | | 94.77 | |  |
| TEO | 8 | | 717 | 0.71-0.99 | 0.87 (0.78-0.95) | | |  | 70.43 (<.001) | | 90.86 | |  |
| Algorithms | | | | | | | | | | | | | |
| SVM | 38 | | 14738 | 0.41-0.99 | 0.79 (0.76-0.83) | | | 0.003 | 753.73 (<.001) | | 95.65 | | 2.02 (.04) |
| CNN | 12 | | 2873 | 0.65-0.99 | 0.84 (0.78-0.89) | | |  | 234.66 (<.001) | | 93.02 | |  |
| Logistic Regression | 12 | | 10863 | 0.65-0.95 | 0.79 (0.72-0.87) | | |  | 906.75 (<.001) | | 98.41 | |  |
| Random Forest | 11 | | 2491 | 0.62-0.88 | 0.72 (0.68-0.77) | | |  | 35.19 (<.001) | | 74.89 | |  |
| DNN | 7 | | 3042 | 0.68-0.98 | 0.84 (0.74-0.94) | | |  | 218.68 (<.001) | | 97.12 | |  |
| Ensemble Model | 7 | | 1554 | 0.72-0.87 | 0.79 (0.74-0.84) | | |  | 20.87 (.002) | | 69.54 | |  |
| GMM | 7 | | 759 | 0.67-0.94 | 0.81 (0.71-0.91) | | |  | 58.45 (<.001) | | 88.95 | |  |
| kNN | 7 | | 1321 | 0.63-0.95 | 0.71 (0.61-0.82) | | |  | 83.20 (<.001) | | 92.40 | |  |
| Naïve Bayes | 6 | | 685 | 0.61-0.76 | 0.68 (0.62-0.74) | | |  | 7.83 (.17) | | 38.34 | |  |
| Speech-eliciting tasks | | | | | | | | | | | | | |
| Free speech | 117 | | 55243 | 0.29-0.99 | 0.80 (0.78-0.83) | | | 0.003 | 3116.16 (<.001) | | 97.48 | | 0.32 (.87) |
| Reading | 58 | | 10988 | 0.62-0.95 | 0.81 (0.78-0.85) | | |  | 700.77 (<.001) | | 91.78 | |  |
| Counting | 6 | | 2032 | 0.66-0.91 | 0.81 (0.56-1.06) | | |  | 129.22 (<.001) | | 97.48 | |  |
| Sustained vowel | 6 | | 2343 | 0.66-0.83 | 0.77 (0.62-0.91) | | |  | 51.62 (<.001) | | 93.21 | |  |
| Ground truth assessment | | | | | | | | | | | | | |
| PHQ-8, and PHQ-9 | 75 | | 19781 | 0.51-0.98 | 0.81 (0.78-0.83) | | | 0.003 | 1223.99 (<.001) | | 93.51 | | 0.26 (.96) |
| BDI, and BDI-II | 24 | | 3509 | 0.29-0.92 | 0.80 (0.73-0.87) | | |  | 299.29 (<.001) | | 95.38 | |  |
| HAM-D | 19 | | 4678 | 0.61-0.90 | 0.80 (0.74-0.86) | | |  | 134.03 (<.001) | | 90.48 | |  |
| DSM, DSM-IV | 21 | | 2780 | 0.62-0.99 | 0.82 (0.75-0.89) | | |  | 398.02 (<.001) | | 92.73 | |  |
| MINI | 6 | | 1043 | 0.63-0.82 | 0.75 (0.63-0.86) | | |  | 22.44 (<.001) | | 82.85 | |  |
| CIDI | 5 | | 32304 | 0.75-0.95 | 0.86 (0.61-1.11) | | |  | 1052.22 (<.001) | | 99.85 | |  |
| Validation approach | | | | | | | | | | | | | |
| Hold-out | 86 | | 50302 | 0.51-0.99 | 0.82 (0.80-0.85) | | | 0.003 | 1426.27 (<.001) | | 97.11 | | 0.65 (.58) |
| K-fold | 63 | | 14052 | 0.29-0.98 | 0.80 (0.76-0.85) | | |  | 971.51 (<.001) | | 95.11 | |  |
| Leave-one-out | 22 | | 2772 | 0.62-0.94 | 0.80 (0.75-0.86) | | |  | 216.13 (<.001) | | 89.03 | |  |
| Dataset | | | | | | | | | | | | | |
| Hand-crafted | 94 | | 16050 | 0.29-0.99 | 0.79 (0.76-0.82) | | | 0.003 | 1123.11 (<.001) | | 92.73 | | 0.92 (.47) |
| DAIC | 42 | | 13070 | 0.51-0.98 | 0.81 (0.77-0.84) | | |  | 949.30 (<.001) | | 95.75 | |  |
| AVEC | 6 | | 1502 | 0.67-0.92 | 0.85 (0.76-0.94) | | |  | 46.76 (<.001) | | 93.51 | |  |
| MODMA | 6 | | 978 | 0.68-0.95 | 0.86 (0.74-0.98) | | |  | 58.84 (<.001) | | 93.73 | |  |
| CONVERGE | 5 | | 32304 | 0.75-0.95 | 0.86 (0.61-1.11) | | |  | 1052.22 (<.001) | | 99.85 | |  |
| Dataset language | | | | | | | | | | | | | |
| English | 59 | | 15565 | 0.51-0.99 | 0.80 (0.77-0.83) | | | 0.003 | 1337.73 (<.001) | | 95.53 | | 1.18 (.32) |
| Chinese | 51 | | 43583 | 0.62-0.95 | 0.81 (0.78-0.85) | | |  | 1484.35 (<.001) | | 97.27 | |  |
| Hungarian | 9 | | 1633 | 0.76-0.92 | 0.85 (0.81-0.90) | | |  | 39.08 (<.001) | | 78.67 | |  |
| German | 7 | | 1150 | 0.76-0.94 | 0.91 (0.89-0.93) | | |  | 12.71 (.05) | | 0.00 | |  |
| Korean | 7 | | 3945 | 0.66-0.82 | 0.76 (0.68-0.84) | | |  | 64.78 (<.001) | | 88.93 | |  |
| Portuguese | 6 | | 198 | 0.61-0.88 | 0.78 (0.67-0.88) | | |  | 10.90 (.05) | | 54.78 | |  |
| Mean age | | | | | | | | | | | | | |
| < 35 | 14 | | 1383 | 0.67-0.95 | 0.84 (0.78-0.90) | | | 0.003 | 88.61 (<.001) | | 86.41 | | 1.70 (.19) |
| > 35 | 38 | | 38733 | 0.61-0.92 | 0.78 (0.74-0.81) | | |  | 471.52 (<.001) | | 96.65 | |  |
| Sample size | | | | | | | | | | | | | |
| < 100 | 43 | | 2713 | 0.29-0.99 | 0.82 (0.76-0.87) | | | 0.003 | 475.79 (<.001) | | 93.36 | | 0.69 (.50) |
| > 100 | 104 | | 58110 | 0.51-0.98 | 0.81 (0.78-0.83) | | |  | 1741.66 (<.001) | | 96.52 | |  |
| AVEC Audio/Visual Emotion Challenge, BDI Beck Depression Inventory, BDI-II Beck Depression Inventory-II, CIDI Composite International Diagnostic Interview, CNN Convolutional Neural Network, CONVERGE China, Oxford and Virginia Commonwealth University Experimental Research on Genetic Epidemiology, DAIC-WOZ Distress Analysis Interview Corpus Wizard-of-Oz, DNN Deep Neural Network, DSM Diagnostic and Statistical Manual of Mental Disorders, DSM-IV Diagnostic and Statistical Manual of Mental Disorders, Fourth Edition, GMM Gaussian Mixture Models, HAM-D Hamilton Depression Rating Scale, kNN K-Nearest Neighbours, MINI Mini-International Neuropsychiatric Interview, MODMA Multi-modal Open Dataset for Mental-disorder Analysis, PHQ-8 Patient Health Questionnaire-8, PHQ-9 Patient Health Questionnaire-9, SVM Support Vector Machine, TEO Teager Energy Operator | | | | | | | | | | | | | |

**Table S5.** Estimated pooled mean of lowest accuracy by several factors.

| Groups | Number of studies | Sample size | Accuracy (%) | Pooled mean accuracy | Heterogeneity measures | | | Test for subgroups differences |
| --- | --- | --- | --- | --- | --- | --- | --- | --- |
|  | Total N | Total N | Range | Mean (%) (95% CI) | Tau^2^ | Q (p-value) | I^2^ (%) | F (p-value) |
| Type of publication | | | | | | | | |
| Journal article | 59 | 16373 | 0.44-0.91 | 0.68 (0.63-0.72) | 0.005 | 843.38 (<.001) | 94.99 | 0.42 (0.66) |
| Conference paper | 53 | 9141 | 0.23-0.94 | 0.64 (0.60-0.69) |  | 735.26 (<.001) | 93.43 |  |
| Speech features | | | | | | | | |
| Spectral features | 98 | 22544 | 0.23-0.94 | 0.66 (0.62-0.70) | 0.005 | 1888.97 (<.001) | 94.99 | 0.50 (.80) |
| Prosodic features | 70 | 17000 | 0.23-0.91 | 0.65 (0.61-0.70) |  | 1349.65 (<.001) | 95.30 |  |
| Source features | 61 | 14238 | 0.35-0.91 | 0.67 (0.62-0.71) |  | 1244.65 (<.001) | 94.66 |  |
| Format features | 39 | 9371 | 0.35-0.88 | 0.65 (0.60-0.71) |  | 773.77 (<.001) | 95.28 |  |
| Lexical features | 10 | 4142 | 0.56-0.88 | 0.67 (0.58-0.76) |  | 61.15 (<.001) | 93.23 |  |
| TEO | 8 | 717 | 0.35-0.87 | 0.62 (0.47-0.78) |  | 141.46 (<.001) | 93.99 |  |
| Algorithms | | | | | | | | |
| SVM | 30 | 6024 | 0.35-0.88 | 0.61 (0.57-0.65) | 0.005 | 272.91 (<.001) | 91.18 | 1.59 (.15) |
| Random Forest | 11 | 2491 | 0.44-0.84 | 0.62 (0.55-0.68) |  | 85.86 (<.001) | 88.17 |  |
| CNN | 10 | 2486 | 0.49-0.92 | 0.72 (0.63-0.81) |  | 218.84 (<.001) | 94.58 |  |
| Logistic Regression | 7 | 2705 | 0.49-0.91 | 0.63 (0.50-0.76) |  | 249.35 (<.001) | 96.21 |  |
| GMM | 6 | 677 | 0.42-0.84 | 0.63 (0.46-0.80) |  | 70.68 (<.001) | 92.97 |  |
| kNN | 6 | 1102 | 0.45-0.78 | 0.57 (0.44-0.71) |  | 49.69 (<.001) | 91.34 |  |
| DNN | 5 | 2823 | 0.64-0.94 | 0.74 (0.58-0.91) |  | 212.33 (<.001) | 97.35 |  |
| Speech-eliciting tasks | | | | | | | | |
| Free speech | 92 | 21675 | 0.23-0.94 | 0.66 (0.63-0.69) | 0.005 | 1532.67 (<.001) | 94.37 | 0.66 (.62) |
| Reading | 42 | 8190 | 0.45-0.91 | 0.68 (0.63-0.73) |  | 639.50 (<.001) | 92.12 |  |
| Counting | 6 | 2032 | 0.45-0.82 | 0.64 (0.32-0.96) |  | 143.81 (<.001) | 97.99 |  |
| Sustained vowel | 6 | 2343 | 0.45-0.65 | 0.53 (0.45-0.62) |  | 45.95 (<.001) | 89.11 |  |
| Ground truth assessment | | | | | | | | |
| PHQ-8, and PHQ-9 | 67 | 18501 | 0.35-0.94 | 0.66 (0.63-0.70) | 0.005 | 1252.48 (<.001) | 94.31 | 1.01 (.41) |
| BDI, and BDI-II | 19 | 2715 | 0.23-0.82 | 0.66 (0.57-0.74) |  | 208.07 (<.001) | 93.17 |  |
| DSM, DSM-IV | 19 | 2502 | 0.42-0.82 | 0.59 (0.51-0.68) |  | 126.25 (<.001) | 87.74 |  |
| HAM-D | 7 | 3103 | 0.50-0.84 | 0.72 (0.61-0.83) |  | 38.41 (<.001) | 94.66 |  |
| Validation approach | | | | | | | | |
| Hold-out | 65 | 16718 | 0.45-0.94 | 0.68 (0.65-0.72) | 0.005 | 1116.82 (<.001) | 93.90 | 1.25 (.29) |
| K-fold | 49 | 12428 | 0.23-0.88 | 0.64 (0.59-0.70) |  | 780.86 (<.001) | 95.33 |  |
| Leave-one-out | 14 | 2181 | 0.37-0.77 | 0.63 (0.56-0.71) |  | 89.93 (<.001) | 85.51 |  |
| Dataset | | | | | | | | |
| Hand-crafted | 72 | 12405 | 0.23-0.92 | 0.64 (0.59-0.69) | 0.005 | 1086.03(<.001) | 94.00 | 1.06 (.38) |
| DAIC-WOZ | 36 | 12056 | 0.35-0.94 | 0.69 (0.65-0.74) |  | 830.00 (<.001) | 95.30 |  |
| AVEC | 5 | 1418 | 0.54-0.82 | 0.68 (0.58-0.79) |  | 45.65 (<.001) | 88.15 |  |
| MODMA | 5 | 978 | 0.65-0.78 | 0.71 (0.66-0.75) |  | 5.58 (.35) | 20.81 |  |
| Dataset language | | | | | | | | |
| English | 45 | 13204 | 0.35-0.94 | 0.66 (0.62-0.70) | 0.005 | 891.74 (<.001) | 94.50 | 1.22 (.31) |
| Chinese | 42 | 12304 | 0.42-0.83 | 0.66 (0.62-0.71) |  | 587.82 (<.001) | 93.90 |  |
| Hungarian | 6 | 997 | 0.64-0.77 | 0.71 (0.65-0.77) |  | 9.75 (.08) | 54.63 |  |
| Korean | 6 | 3741 | 0.45-0.66 | 0.58 (0.40-0.75) |  | 106.61 (<.001) | 95.87 |  |
| German | 5 | 950 | 0.69-0.88 | 0.77 (0.67-0.86) |  | 20.06 (<.001) | 80.46 |  |
| Mean age | | | | | | | | |
| < 35 | 10 | 879 | 0.62-0.88 | 0.76 (0.68-0.83) | 0.005 | 39.87 (.002) | 78.95 | 2.38 (.10) |
| > 35 | 23 | 7007 | 0.44-0.91 | 0.63 (0.55-0.71) |  | 564.51 (<.001) | 95.75 |  |
| Sample size | | | | | | | | |
| < 100 | 22 | 2197 | 0.23-0.92 | 0.63 (0.57-0.70) | 0.005 | 344.73 (<.001) | 88.88 | 0.82 (.37) |
| > 100 | 80 | 23822 | 0.44-0.94 | 0.67 (0.64-0.71) |  | 1658.10 (<.001) | 94.77 |  |
| AVEC Audio/Visual Emotion Challenge, BDI Beck Depression Inventory, BDI-II Beck Depression Inventory-II, CNN Convolutional Neural Network, DAIC-WOZ Distress Analysis Interview Corpus Wizard-of-Oz, DNN Deep Neural Network, DSM Diagnostic and Statistical Manual of Mental Disorders, DSM-IV Diagnostic and Statistical Manual of Mental Disorders, Fourth Edition, GMM Gaussian Mixture Models, HAM-D Hamilton Depression Rating Scale, kNN K-Nearest Neighbours, MODMA Multi-modal Open Dataset for Mental-disorder Analysis, PHQ-8 Patient Health Questionnaire-8, PHQ-9 Patient Health Questionnaire-9, SVM Support Vector Machine, TEO Teager Energy Operator | | | | | | | | |

**Table S6.** Estimated pooled mean of highest sensitivity by several factors.

| Groups | Number of studies | Sample size | Sensitivity (%) | Pooled mean sensitivity | Heterogeneity measures | | | Test for subgroups differences |  |
| --- | --- | --- | --- | --- | --- | --- | --- | --- | --- |
|  | Total N | Total N | Range | Mean (%) (95% CI) | Tau^2^ | Q (p-value) | I^2^ (%) | F (p-value) |  |
| Type of publication | | | | | | | | |  |
| Journal Article | 74 | 55493 | 0.55-1.00 | 0.85 (0.82-0.89) | 0.007 | 9649.75 (<.001) | 99.92 | 1.32 (.27) |  |
| Conference paper | 59 | 10439 | 0.25-1.00 | 0.81 (0.76-0.86) |  | 2911.56 (<.001) | 99.82 |  |  |
| Speech features | | | | | | | | | |
| Spectral features | 120 | 54119 | 0.25-1.00 | 0.83 (0.80-0.86) | 0.008 | 10697.55 (<.001) | 99.92 | 2.14 (.05) |  |
| Prosodic features | 85 | 47458 | 0.25-1.00 | 0.82 (0.77-0.86) |  | 9513.156 (<.001) | 99.94 |  |  |
| Source features | 79 | 44789 | 0.45-1.00 | 0.83 (0.80-0.87) |  | 8801.78 (<.001) | 99.93 |  |  |
| Format features | 55 | 41180 | 0.25-1.00 | 0.82 (0.76-0.87) |  | 7703.99 (<.001) | 99.94 |  |  |
| Lexical features | 16 | 4787 | 0.71-1.00 | 0.87 (0.82-0.93) |  | 86.44 (<.001) | 98.22 |  |  |
| TEO | 9 | 777 | 0.57-1.00 | 0.87 (0.77-0.97) |  | 141.30 (<.001) | 99.95 |  |  |
| Algorithms | | | | | | | | | |
| SVM | 34 | 13447 | 0.39-1.00 | 0.82 (0.77-0.87) | 0.007 | 2442.63 (<.001) | 99.95 | 0.68 (.75) |  |
| Logistic Regression | 14 | 12749 | 0.63-0.98 | 0.84 (0.77-0.91) |  | 1088.42 (<.001) | 99.16 |  |  |
| CNN | 10 | 1958 | 0.52-1.00 | 0.86 (0.72-0.96) |  | 467.85 (<.001) | 99.95 |  |  |
| Random Forest | 7 | 1101 | 0.61-0.91 | 0.74 (0.66-0.81) |  | 22.67 (<.001) | 80.87 |  |  |
| DNN | 6 | 12049 | 0.71-1.00 | 0.87 (0.78-0.96) |  | 89.47 (<.001) | 98.35 |  |  |
| GMM | 6 | 680 | 0.55-0.93 | 0.73 (0.59-0.86) |  | 54.78 (<.001) | 90.33 |  |  |
| kNN | 6 | 1137 | 0.63-0.95 | 0.75 (0.58-0.91) |  | 182.55 (<.001) | 96.79 |  |  |
| Ensemble Model | 5 | 834 | 0.77-1.00 | 0.83 (0.69-0.96) |  | 184.77 (<.001) | 95.07 |  |  |
| MLP | 5 | 8075 | 0.45-1.00 | 0.81 (0.55-1.07) |  | 1303.55 (<.001) | 99.92 |  |  |
| Naïve Bayes | 5 | 450 | 0.63-0.89 | 0.79 (0.64-0.94) |  | 37.53 (<.001) | 86.63 |  |  |
| Speech-eliciting tasks | | | | | | | | | |
| Free speech | 106 | 60804 | 0.25-1.00 | 0.83 (0.80-0.87) | 0.008 | 11039.67 (<.001) | 99.92 | 0.25 (.91) |  |
| Reading | 52 | 10366 | 0.61-1.00 | 0.84 (0.80-0.88) |  | 2862.53 (<.001) | 99.59 |  |  |
| Counting | 6 | 2032 | 0.65-0.91 | 0.81 (0.55-1.06) |  | 140.44 (<.001) | 97.67 |  |  |
| Sustained vowel | 6 | 2343 | 0.65-0.82 | 0.76 (0.62-0.90) |  | 51.78 (<.001) | 93.06 |  |  |
| Ground truth assessment | | | | | | | | | |
| PHQ-8, and PHQ-9 | 67 | 24549 | 0.39-1.00 | 0.82 (0.78-0.87) | 0.007 | 5349.36 (<.001) | 99.93 | 0.49 (.79) |  |
| BDI, and BDI-II | 19 | 3044 | 0.25-1.00 | 0.86 (0.76-0.95) |  | 793.38 (<.001) | 99.21 |  |  |
| HAM-D | 23 | 4990 | 0.55-1.00 | 0.84 (0.76-0.91) |  | 903.89 (<.001) | 98.30 |  |  |
| DSM, DSM-IV | 20 | 2720 | 0.57-1.00 | 0.80 (0.71-0.89) |  | 1012.09 (<.001) | 99.89 |  |  |
| CIDI | 7 | 43449 | 0.81-0.98 | 0.90 (0.83-0.98) |  | 1189.82 (<.001) | 99.57 |  |  |
| Validation approach | | | | | | | | |  |
| Hold-out | 87 | 60950 | 0.39-1.00 | 0.84 (0.81-0.87) | 0.007 | 10840.03 (<.001) | 99.94 | 0.51 (.68) |  |
| K-fold | 60 | 11971 | 0.25-1.00 | 0.83 (0.78-0.88) |  | 3414.55 (<.001) | 99.86 |  |  |
| Leave-one-out | 18 | 2077 | 0.52-0.96 | 0.80 (0.73-0.88) |  | 246.79 (<.001) | 94.37 |  |  |
| Dataset |  |  |  |  |  |  |  |  |  |
| Hand-crafted | 85 | 13795 | 0.25-1.00 | 0.82 (0.78-0.86) | 0.007 | 3870.80 (<.001) | 99.59 | 1.07 (.38) |  |
| DAIC-WOZ | 40 | 20962 | 0.39-1.00 | 0.82 (0.76-0.87) |  | 4225.97 (<.001) | 99.95 |  |  |
| AVEC | 6 | 1761 | 0.75-1.00 | 0.90 (0.79-1.00) |  | 223.45 (<.001) | 99.95 |  |  |
| CONVERGE | 6 | 41641 | 0.81-0.98 | 0.90 (0.79-1.00) |  | 1128.55 (<.001) | 99.70 |  |  |
| MODMA | 5 | 733 | 0.87-0.95 | 0.90 (0.87-0.93) |  | 4.90 (.30) | 0.00 |  |  |
| Dataset language | | | | | | | | |  |
| English | 57 | 23285 | 0.39-1.00 | 0.81 (0.77-0.86) | 0.007 | 4972.05 (<.001) | 99.96 | 0.53 (.81) |  |
| Chinese | 46 | 51512 | 0.63-1.00 | 0.85 (0.81-0.89) |  | 6448.45 (<.001) | 99.51 |  |  |
| German | 8 | 1493 | 0.55-1.00 | 0.88 (0.76-1.00) |  | 264.16 (<.001) | 99.06 |  |  |
| Hungarian | 7 | 1437 | 0.78-0.93 | 0.90 (0.88-0.92) |  | 5.88 (.44) | 14.10 |  |  |
| Korean | 7 | 3945 | 0.65-0.91 | 0.78 (0.62-0.94) |  | 268.06 (.01) | 96.91 |  |  |
| Portuguese | 6 | 198 | 0.78-1.00 | 0.89 (0.80-0.99) |  | 30.21 (<.001) | 75.44 |  |  |
| Malay | 5 | 336 | 0.77-0.95 | 0.85 (0.64-1.06) |  | 15.20 (.004) | 83.90 |  |  |
| Mean age | | | | | | | | |  |
| < 35 | 19 | 1677 | 0.55-0.98 | 0.87 (0.80-0.94) | 0.007 | 160.92 (<.001) | 93.69 | 0.53 (.59) |  |
| > 35 | 31 | 34441 | 0.25-1.00 | 0.82 (0.76-0.87) |  | 5121.43 (<.001) | 99.27 |  |  |
| Sample size | | | | | | | | |  |
| < 100 | 47 | 2933 | 0.25-1.00 | 0.84 (0.78-0.91) | 0.007 | 997.66 (<.001) | 99.96 | 0.09 (.77) |  |
| > 100 | 88 | 63504 | 0.39-1.00 | 0.83 (0.80-0.86) |  | 11274.34 (<.001) | 99.88 |  |  |
| AVEC Audio/Visual Emotion Challenge, BDI Beck Depression Inventory, BDI-II Beck Depression Inventory-II, CNN Convolutional Neural Network, DAIC-WOZ Distress Analysis Interview Corpus Wizard-of-Oz, CONVERGE China, Oxford and Virginia Commonwealth University Experimental Research on Genetic Epidemiology, DNN Deep Neural Network, DSM Diagnostic and Statistical Manual of Mental Disorders, DSM-IV Diagnostic and Statistical Manual of Mental Disorders, Fourth Edition, GMM Gaussian Mixture Models, GMM Gaussian Mixture Models, HAM-D Hamilton Depression Rating Scale, PHQ-8 Patient Health Questionnaire-8, PHQ-9 Patient Health Questionnaire-9, SVM Support Vector Machine, TEO Teager Energy Operator | | | | | | | | | |

**Table S7.** Estimated pooled mean of lowest sensitivity by several factors.

| Groups | Number of studies | Sample size | Sensitivity (%) | Pooled mean sensitivity | Heterogeneity measures | | | Test for subgroups  differences |  |
| --- | --- | --- | --- | --- | --- | --- | --- | --- | --- |
|  | Total N | Total N | Range | Mean (%) (95% CI) | Tau^2^ | Q (p-value) | I^2^ (%) | F (p-value) |  |
| Type of publication | | | | | | | | | |
| Journal Article | 59 | 23774 | 0.09-0.94 | 0.62 (0.55-0.68) | 0.010 | 7317.85 (<.001) | 98.64 | 0.13 (.88) |  |
| Conference paper | 44 | 7716 | 0.00-0.98 | 0.65 (0.57-0.72) |  | 24286.06 (<.001) | 99.90 |  |  |
| Speech features | | | | | | | | | |
| Spectral features | 91 | 19793 | 0.00-0.98 | 0.64 (0.58-0.69) | 0.010 | 48326.42 (<.001) | 99.82 | 0.40 (.88) |  |
| Prosodic features | 68 | 14616 | 0.00-0.94 | 0.63 (0.56-0.69) |  | 37580.63 (<.001) | 99.87 |  |  |
| Source features | 63 | 12156 | 0.00-0.94 | 0.64 (0.57-0.71) |  | 34681.20 (<.001) | 99.88 |  |  |
| Format features | 42 | 8787 | 0.00-0.92 | 0.65 (0.55-0.74) |  | 26799.34 (<.001) | 99.93 |  |  |
| Lexical features | 12 | 4278 | 0.24-0.94 | 0.66 (0.51-0.82) |  | 487.19 (<.001) | 98.589 |  |  |
| TEO | 9 | 777 | 0.50-0.86 | 0.63 (0.48-0.77) |  | 81.163 (<.001) | 89.68 |  |  |
| Algorithms | | | | | | | | | |
| SVM | 27 | 4807 | 0.00-0.94 | 0.62 (0.53-0.70) | 0.011 | 11141.26 (<.001) | 98.71 | 0.63 (.73) |  |
| Logistic Regression | 9 | 4591 | 0.09-0.94 | 0.56 (0.34-0.77) |  | 5581.07 (<.001) | 99.61 |  |  |
| Random Forest | 7 | 1101 | 0.19-0.69 | 0.53 (0.40-0.67) |  | 42.63 (<.001) | 92.55 |  |  |
| CNN | 8 | 1571 | 0.47-0.98 | 0.69 (0.55-0.84) |  | 282.46 (<.001) | 97.08 |  |  |
| GMM | 5 | 598 | 0.24-0.67 | 0.50 (0.30-0.70) |  | 55.42 (<.001) | 92.53 |  |  |
| kNN | 5 | 918 | 0.43-0.82 | 0.60 (0.36-0.83) |  | 126.32 (<.001) | 96.50 |  |  |
| Speech-eliciting tasks | | | | | | | | | |
| Free speech | 84 | 27658 | 0.00-0.98 | 0.63 (0.57-0.69) | 0.009 | 53601.90 (<.001) | 98.84 | 0.62 (.66) |  |
| Reading | 39 | 7930 | 0.36-0.94 | 0.67 (0.60-0.73) |  | 1085.63 (<.001) | 95.20 |  |  |
| Counting | 6 | 2032 | 0.45-0.80 | 0.63 (0.26-1.00) |  | 152.30 (<.001) | 98.36 |  |  |
| Sustained vowel | 6 | 2343 | 0.45-0.54 | 0.51 (0.44-0.58) |  | 10.35 (.07) | 68.59 |  |  |
| Ground truth assessment | | | | | | | | | |
| PHQ-8, and PHQ-9 | 60 | 23369 | 0.00-0.92 | 0.62 (0.55-0.69) | 0.010 | 39825.20 (<.001) | 99.86 | 0.65 (.63) |  |
| BDI, and BDI-II | 16 | 2408 | 0.25-0.84 | 0.68 (0.59-0.76) |  | 184.02 (<.001) | 92.95 |  |  |
| DSM, DSM-IV | 18 | 2442 | 0.45-0.77 | 0.58 (0.51-0.65) |  | 71.30 (<.001) | 77.05 |  |  |
| HAM-D | 11 | 3415 | 0.19-0.89 | 0.72 (0.58-0.86) |  | 276.66 (<.001) | 97.92 |  |  |
| Validation approach | | | | | | | | | |
| Hold-out | 68 | 27654 | 0.00-0.98 | 0.62 (0.56-0.68) | 0.010 | 52771.79 (<.001) | 99.87 | 0.27 (.85) |  |
| K-fold | 49 | 10725 | 0.09-0.94 | 0.64 (0.56-0.72) |  | 5672.40 (<.001) | 97.98 |  |  |
| Leave-one-out | 14 | 1486 | 0.24-0.88 | 0.61 (0.46-0.75) |  | 185.22 (<.001) | 94.98 |  |  |
| Dataset | | | | | | | | | |
| Hand-crafted | 66 | 11181 | 0.09-0.98 | 0.64 (0.58-0.71) | 0.010 | 7288.51 (<.001) | 97.63 | 0.56 (.69) |  |
| DAIC-WOZ | 36 | 20048 | 0.00-0.92 | 0.63 (0.54-0.72) |  | 34650.23 (<.001) | 99.93 |  |  |
| AVEC | 6 | 1761 | 0.42-0.83 | 0.60 (0.47-0.74) |  | 149.95 (<.001) | 95.42 |  |  |
| MODMA | 5 | 733 | 0.67-0.79 | 0.74 (0.65-0.82) |  | 9.85 (.04) | 63.70 |  |  |
| Dataset language | | | | | | | | | |
| English | 45 | 21108 | 0.00-0.92 | 0.60 (0.53-0.67) | 0.010 | 35514.62 (<.001) | 99.90 | 0.66 (.66) |  |
| Chinese | 38 | 20307 | 0.09-0.89 | 0.64 (0.56-0.72) |  | 6587.13 (<.001) | 98.78 |  |  |
| Korean | 7 | 3945 | 0.45-0.86 | 0.65 (0.38-0.92) |  | 204.58 (<.001) | 98.61 |  |  |
| German | 6 | 1293 | 0.56-0.94 | 0.73 (0.59-0.87) |  | 97.34 (<.001) | 94.92 |  |  |
| Malay | 5 | 336 | 0.62-0.70 | 0.68 (0.61-0.75) |  | 0.73 (.95) | 0.00 |  |  |
| Mean age | | | | | | | | | |
| < 35 | 16 | 1257 | 0.62-0.89 | 0.75 (0.68-0.82) | 0.010 | 94.85 (<.001) | 83.91 | 2.01 (.24) |  |
| > 35 | 18 | 2993 | 0.19-0.94 | 0.60 (0.47-0.73) |  | 844.89 (<.001) | 97.53 |  |  |
| Sample size | | | | | | | | | |
| < 100 | 40 | 2575 | 0.19-0.98 | 0.65 (0.59-0.72) | 0.010 | 567.32 (<.001) | 91.601 | 0.36 (.55) |  |
| > 100 | 65 | 29420 | 0.00-0.94 | 0.62 (0.55-0.69) |  | 54293.27 (<.001) | 99.89 |  |  |
| AVEC Audio/Visual Emotion Challenge, BDI Beck Depression Inventory, BDI-II Beck Depression Inventory-II, CNN Convolutional Neural Network, DAIC-WOZ Distress Analysis Interview Corpus Wizard-of-Oz, DSM Diagnostic and Statistical Manual of Mental Disorders, DSM-IV Diagnostic and Statistical Manual of Mental Disorders, Fourth Edition, GMM Gaussian Mixture Models, HAM-D Hamilton Depression Rating Scale, PHQ-8 Patient Health Questionnaire-8, PHQ-9 Patient Health Questionnaire-9, SVM Support Vector Machine, TEO Teager Energy Operator, | | | | | | | | | |

**Table S8.** Estimated pooled mean of highest specificity by several factors.

| Groups | Number of studies | Sample size | Specificity (%) | Pooled mean specificity | Heterogeneity measures | | | Test for subgroups  differences |  |
| --- | --- | --- | --- | --- | --- | --- | --- | --- | --- |
|  | Total N | Total N | Range | Mean (%) (95% CI) | Tau^2^ | Q (p-value) | I^2^ (%) | F (p-value) |  |
| Type of publication | | | | | | | | | |
| Journal Article | 49 | 41975 | 0.33-1.00 | 0.83 (0.78-0.88) | 0.006 | 14398.39 (<.001) | 99.38 | 1.97 (.15) |  |
| Conference paper | 26 | 4110 | 0.50-0.98 | 0.82 (0.76-0.87) |  | 443.76 (<.001) | 95.17 |  |  |
| Speech features | | | | | | | | | |
| Spectral features | 68 | 44368 | 0.33-1.00 | 0.82 (0.78-0.86) | 0.006 | 15013.86 (<.001) | 99.18 | 3.61 (.004) |  |
| Prosodic features | 45 | 39177 | 0.38-1.00 | 0.83 (0.78-0.89) |  | 14220.70 (<.001) | 99.42 |  |  |
| Source features | 42 | 36784 | 0.66-1.00 | 0.86 (0.82-0.90) |  | 11106.30 (<.001) | 98.87 |  |  |
| Format features | 29 | 34751 | 0.66-1.00 | 0.86 (0.81-0.91) |  | 10809.60 (<.001) | 99.24 |  |  |
| Lexical features | 8 | 3560 | 0.38-0.96 | 0.75 (0.59-0.90) |  | 1062.90 (<.001) | 98.48 |  |  |
| TEO | 7 | 633 | 0.33-0.99 | 0.88 (0.79-0.97) |  | 65.35 (<.001) | 93.94 |  |  |
| Algorithms | | | | | | | | | |
| SVM | 20 | 11394 | 0.50-1.00 | 0.81 (0.75-0.87) | 0.006 | 4372.76 (<.001) | 99.06 | 0.45 (.81) |  |
| Logistic Regression | 9 | 10553 | 0.62-0.96 | 0.78 (0.70-0.86) |  | 1392.24 (<.001) | 98.42 |  |  |
| DNN | 5 | 2712 | 0.38-0.96 | 0.80 (0.50-1.10) |  | 1322.60 (<.001) | 99.41 |  |  |
| GMM | 5 | 598 | 0.71-0.98 | 0.84 (0.69-0.99) |  | 88.60 (<.001) | 94.60 |  |  |
| Random Forest | 5 | 544 | 0.63-0.84 | 0.76 (0.69-0.84) |  | 8.17 (.09) | 46.77 |  |  |
| Speech-eliciting tasks | | | | | | | | | |
| Free speech | 60 | 43797 | 0.33-1.00 | 0.82 (0.78-0.87) | 0.007 | 16017.53 (<.001) | 99.77 | 0.56 (.57) |  |
| Reading | 31 | 6214 | 0.63-1.00 | 0.86 (0.81-0.90) |  | 1112.12 (<.001) | 99.67 |  |  |
| Ground truth assessment | | | | | | | | | |
| PHQ-8, and PHQ-9 | 37 | 10282 | 0.38-1.00 | 0.81 (0.75-0.87) | 0.007 | 5243.82 (<.001) | 99.83 | 2.20 (.06) |  |
| BDI, and BDI-II | 12 | 2201 | 0.70-1.00 | 0.88 (0.82-0.93) |  | 306.63 (<.001) | 95.14 |  |  |
| DSM, DSM-IV | 10 | 840 | 0.63-0.99 | 0.86 (0.73-0.99) |  | 161.57 (<.001) | 95.94 |  |  |
| HAM-D | 10 | 3104 | 0.33-0.89 | 0.68 (0.54-0.82) |  | 762.91 (<.001) | 97.32 |  |  |
| CIDI | 5 | 32424 | 0.67-0.96 | 0.83 (0.50-1.17) |  | 2409.26 (<.001) | 99.92 |  |  |
| Validation approach | | | | | | | | | |
| Hold-out | 39 | 41945 | 0.38-1.00 | 0.84 (0.79-0.89) | 0.007 | 15480.39 (<.001) | 99.85 | 0.20 (.90) |  |
| K-fold | 37 | 8336 | 0.33-1.00 | 0.83 (0.78-0.88) |  | 1693.36 (<.001) | 98.12 |  |  |
| Leave-one-out | 16 | 1720 | 0.63-0.96 | 0.84 (0.77-0.90) |  | 113.37 (<.001) | 89.17 |  |  |
| Dataset | | | | | | | | | |
| Hand-crafted | 48 | 8319 | 0.33-0.98 | 0.83 (0.80-0.87) | 0.006 | 669.53 (<.001) | 93.35 | 0.02 (.98) |  |
| DAIC-WOZ | 22 | 7742 | 0.38-1.00 | 0.82 (0.74-0.91) |  | 4616.60 (<.001) | 99.93 |  |  |
| Dataset language | | | | | | | | | |
| English | 28 | 8451 | 0.38-1.00 | 0.83 (0.76-0.90) | 0.007 | 4727.46 (<.001) | 99.90 | 1.34 (.26) |  |
| Chinese | 29 | 38809 | 0.38-0.97 | 0.80 (0.72-0.89) |  | 4649.19 (<.001) | 99.47 |  |  |
| Hungarian | 7 | 1437 | 0.74-0.98 | 0.88 (0.81-0.96) |  | 42.20 (<.001) | 88.25 |  |  |
| Portuguese | 6 | 198 | 0.33-0.84 | 0.66 (0.47-0.86) |  | 29.76 (<.001) | 83.52 |  |  |
| Mean age | | | | | | | | | |
| < 35 | 8 | 800 | 0.70-0.96 | 0.85 (0.76-0.95) | 0.007 | 67.50 (<.001) | 89.65 | 2.86 (.06) |  |
| > 35 | 23 | 32545 | 0.33-0.90 | 0.75 (0.70-0.80) |  | 719.56 (<.001) | 98.27 |  |  |
| Sample size | | | | | | | | | |
| < 100 | 25 | 1547 | 0.33-0.99 | 0.82 (0.75-0.90) | 0.007 | 306.35 (<.001) | 94.12 | 0.01 (.94) |  |
| > 100 | 52 | 45043 | 0.38-1.00 | 0.83 (0.79-0.87) |  | 16049.34 (<.001) | 99.76 |  |  |
| BDI Beck Depression Inventory, BDI-II Beck Depression Inventory-II, CIDI Composite International Diagnostic Interview, DAIC-WOZ Distress Analysis Interview Corpus Wizard-of-Oz, DNN Deep Neural Network, GMM Gaussian Mixture Models, HAM-D Hamilton Depression Rating Scale, PHQ-8 Patient Health Questionnaire-8, PHQ-9 Patient Health Questionnaire-9, SVM Support Vector Machine, TEO Teager Energy Operator | | | | | | | | | |

**Table S9.** Estimated pooled mean of lowest specificity by several factors.

| Groups | Number of studies | Sample size | Specificity (%) | Pooled mean specificity | Heterogeneity measures | | | Test for subgroups differences |
| --- | --- | --- | --- | --- | --- | --- | --- | --- |
|  | Total N | Total N | Range | Mean (%) (95% CI) | Tau^2^ | Q (p-value) | I^2^ (%) | F (p-value) |
| Type of publication | | | | | | | | |
| Journal Article | 35 | 10310 | 0.13-0.94 | 0.64 (0.55-0.73) | 0.036 | 5086.36 (<.001) | 98.57 | 1.11 (.34) |
| Conference paper | 18 | 2632 | 0.03-0.93 | 0.58 (0.48-0.68) |  | 1060.71 (<.001) | 96.62 |  |
| Speech features | | | | | | | | |
| Spectral features | 47 | 11341 | 0.03-0.94 | 0.61 (0.55-0.67) | 0.033 | 6229.32 (<.001) | 98.23 | 0.90 (.50) |
| Prosodic features | 34 | 7410 | 0.03-0.94 | 0.61 (0.54-0.69) |  | 5747.26 (<.001) | 98.43 |  |
| Source features | 32 | 5226 | 0.03-0.94 | 0.62 (0.55-0.70) |  | 2298.64 (<.001) | 97.79 |  |
| Format features | 20 | 3249 | 0.03-0.94 | 0.61 (0.49-0.72) |  | 1964.06 (<.001) | 98.36 |  |
| TEO | 7 | 633 | 0.03-0.93 | 0.51 (0.19-0.82) |  | 1179.44 (<.001) | 99.01 |  |
| Lexical features | 5 | 3240 | 0.13-0.81 | 0.52 (0.20-0.83) |  | 926.12 (<.001) | 99.24 |  |
| Algorithms | | | | | | | | |
| SVM | 15 | 2977 | 0.03-0.81 | 0.50 (0.38-0.62) | 0.035 | 841.16 (<.001) | 97.61 | 1.43 (.24) |
| GMM | 5 | 598 | 0.43-0.93 | 0.60 (0.36-0.85) |  | 143.67 (<.001) | 95.90 |  |
| Logistic Regression | 5 | 2528 | 0.46-0.90 | 0.62 (0.41-0.84) |  | 290.01 (<.001) | 97.95 |  |
| Random Forest | 5 | 544 | 0.54-0.78 | 0.67 (0.55-0.79) |  | 18.54 (<.001) | 76.90 |  |
| Speech-eliciting tasks | | | | | | | | |
| Free speech | 44 | 11684 | 0.03-0.93 | 0.59 (0.53-0.65) | 0.036 | 5307.24 (<.001) | 98.18 | 2.88 (.06) |
| Reading | 22 | 4606 | 0.46-0.94 | 0.70 (0.62-0.77) |  | 480.98 (<.001) | 94.37 |  |
| Ground truth assessment | | | | | | | | |
| PHQ-8, and PHQ-9 | 32 | 9370 | 0.03-0.93 | 0.60 (0.53-0.67) | 0.038 | 5185.15 (<.001) | 98.53 | 0.47 (.76) |
| DSM, DSM-IV | 9 | 620 | 0.28-0.94 | 0.55 (0.27-0.84) |  | 231.84 (<.001) | 96.65 |  |
| BDI, and BDI-II | 8 | 1475 | 0.39-0.75 | 0.62 (0.52-0.73) |  | 53.41 (<.001) | 89.99 |  |
| HAM-D | 6 | 2972 | 0.13-0.78 | 0.52 (0.22-0.81) |  | 1094.77 (<.001) | 99.10 |  |
| Validation approach | | | | | | | | |
| K-fold | 27 | 7120 | 0.21-0.94 | 0.60 (0.54-0.66) |  | 608.23 (<.001) | 96.33 |  |
| Hold-out | 27 | 9890 | 0.13-0.91 | 0.62 (0.52-0.71) | 0.038 | 4626.38 (<.001) | 98.77 | 0.07 (.98) |
| Leave-one-out | 12 | 1129 | 0.43-0.71 | 0.61 (0.55-0.68) |  | 21.80 (<.001) | 65.55 |  |
| Dataset | | | | | | | | |
| Hand-crafted | 35 | 6673 | 0.21-0.94 | 0.63 (0.58-0.68) | 0.036 | 715.20 (<.001) | 93.99 | 1.52 (.23) |
| DAIC-WOZ | 18 | 7161 | 0.03-0.93 | 0.59 (0.46-0.72) |  | 4812.94 (<.001) | 99.36 |  |
| Dataset language | | | | | | | | |
| Chinese | 23 | 7870 | 0.13-0.94 | 0.67 (0.53-0.81) | 0.036 | 4344.33 (<.001) | 98.92 | 1.45 (.24) |
| English | 23 | 7543 | 0.03-0.93 | 0.56 (0.46-0.67) |  | 4853.54 (<.001) | 99.11 |  |
| Mean age | | | | | | | | |
| < 35 | 5 | 372 | 0.67-0.94 | 0.81 (0.65-0.96) | 0.034 | 29.66 (<.001) | 84.24 | 2.72 (.08) |
| > 35 | 11 | 1159 | 0.21-0.90 | 0.62 (0.50-0.74) |  | 200.16 (<.001) | 93.58 |  |
| Sample size | | | | | | | | |
| < 100 | 19 | 1239 | 0.03-0.93 | 0.56 (0.44-0.67) | 0.036 | 1089.80 (<.001) | 96.14 | 1.59 (.21) |
| > 100 | 36 | 12208 | 0.13-0.94 | 0.64 (0.56-0.72) |  | 5201.73 (<.001) | 98.42 |  |
| BDI Beck Depression Inventory, BDI-II Beck Depression Inventory-II, DAIC-WOZ Distress Analysis Interview Corpus Wizard-of-Oz, GMM Gaussian Mixture Models, HAM-D Hamilton Depression Rating Scale, PHQ-8 Patient Health Questionnaire-8, PHQ-9 Patient Health Questionnaire-9, SVM Support Vector Machine, TEO Teager Energy Operator | | | | | | | | |

**Table S10.** Estimated pooled mean of highest precision by several factors.

| Groups | Number of studies | Sample size | Precision (%) | Pooled mean precision | Heterogeneity measures | | | Test for subgroups differences |
| --- | --- | --- | --- | --- | --- | --- | --- | --- |
|  | Total N | Total N | Range | Mean (%) (95% CI) | Tau^2^ | Q (p-value) | I^2^ (%) | F (p-value) |
| Type of publication | | | | | | | | |
| Journal Article | 51 | 21066 | 0.42-1.00 | 0.82 (0.77-0.86) | 0.005 | 7419.04 (<.001) | 99.87 | 0.49 (.62) |
| Conference paper | 43 | 8987 | 0.25-0.99 | 0.79 (0.73-0.85) |  | 1180.68 (<.001) | 98.19 |  |
| Speech features | | | | | | | | |
| Spectral features | 87 | 19136 | 0.25-1.00 | 0.80 (0.76-0.84) | 0.005 | 6151.30 (<.001) | 99.70 | 1.40 (.23) |
| Prosodic features | 59 | 14079 | 0.25-1.00 | 0.77 (0.72-0.83) |  | 4886.21 (<.001) | 99.83 |  |
| Source features | 55 | 11592 | 0.42-1.00 | 0.79 (0.74-0.84) |  | 4134.07 (<.001) | 99.78 |  |
| Format features | 40 | 9283 | 0.42-1.00 | 0.78 (0.70-0.85) |  | 3620.87 (<.001) | 99.87 |  |
| Lexical features | 17 | 5207 | 0.45-1.00 | 0.80 (0.72-0.89) |  | 1302.02 (<.001) | 99.045 |  |
| Algorithms | | | | | | | | |
| SVM | 22 | 4772 | 0.28-1.00 | 0.79 (0.71-0.87) | 0.005 | 1296.10 (<.001) | 99.09 | 1.06 (.39) |
| CNN | 10 | 1958 | 0.46-0.99 | 0.79 (0.69-0.89) |  | 260.54 (<.001) | 96.88 |  |
| Logistic Regression | 9 | 2954 | 0.46-0.93 | 0.81 (0.70-0.92) |  | 224.13 (<.001) | 97.41 |  |
| DNN | 5 | 11827 | 0.35-0.97 | 0.87 (0.74-0.99) |  | 599.66 (<.001) | 98.94 |  |
| kNN | 5 | 967 | 0.47-0.95 | 0.70 (0.47-0.92) |  | 124.95 (<.001) | 97.10 |  |
| Random Forest | 5 | 1025 | 0.50-0.80 | 0.62 (0.46-0.77) |  | 46.49 (<.001) | 92.52 |  |
| Speech-eliciting tasks | | | | | | | | |
| Free speech | 75 | 26261 | 0.25-1.00 | 0.81 (0.76-0.85) | 0.005 | 8350.86 (<.001) | 99.86 | 1.16 (.34) |
| Reading | 37 | 8128 | 0.53-1.00 | 0.84 (0.80-0.89) |  | 2135.89 (<.001) | 99.75 |  |
| Counting | 6 | 2032 | 0.53-0.92 | 0.79 (0.46-1.11) |  | 236.74 (<.001) | 98.52 |  |
| Sustained vowel | 6 | 2343 | 0.53-1.00 | 0.83 (0.40-1.25) |  | 818.77 (<.001) | 99.41 |  |
| Ground truth assessment | | | | | | | | |
| PHQ-8, and PHQ-9 | 55 | 22835 | 0.35-1.00 | 0.80 (0.75-0.85) | 0.005 | 7412.70 (<.001) | 99.78 | 0.66 (.63) |
| BDI, and BDI-II | 15 | 2538 | 0.25-1.00 | 0.80 (0.68-0.92) |  | 941.66 (<.001) | 99.16 |  |
| HAM-D | 15 | 4444 | 0.58-1.00 | 0.87 (0.80-0.96) |  | 814.33 (<.001) | 98.13 |  |
| DSM, DSM-IV | 12 | 2220 | 0.53-0.93 | 0.77 (0.67-0.87) |  | 144.65 (<.001) | 91.58 |  |
| Validation approach | | | | | | | | |
| Hold-out | 77 | 28415 | 0.35-1.00 | 0.80 (0.76-0.84) | 0.005 | 9042.91 (<.001) | 99.82 | 0.39 (.76) |
| K-fold | 38 | 7543 | 0.25-1.00 | 0.83 (0.75-0.91) |  | 2877.97 (<.001) | 99.95 |  |
| Leave-one-out | 8 | 1309 | 0.46-0.93 | 0.76 (0.63-0.90) |  | 183.03 (<.001) | 96.39 |  |
| Dataset | | | | | | | | |
| Hand-crafted | 53 | 8631 | 0.25-1 .00 | 0.81 (0.76-0.86) | 0.005 | 2732.47 (<.001) | 98.25 | 0.99 (.42) |
| DAIC-WOZ | 41 | 21274 | 0.35-1.00 | 0.78 (0.72-0.84) |  | 6864.25 (<.001) | 99.33 |  |
| AVEC | 6 | 1761 | 0.73-1.00 | 0.89 (0.80-0.98) |  | 279.76 (<.001) | 97.85 |  |
| MODMA | 5 | 733 | 0.77-0.95 | 0.86 (0.76-0.97) |  | 25.57 (<.001) | 86.32 |  |
| Dataset language | | | | | | | | |
| English | 51 | 23155 | 0.35-1.00 | 0.78 (0.73-0.83) | 0.005 | 7358.81 (<.001) | 99.15 | 1.85 (.11) |
| Chinese | 26 | 16970 | 0.60-1.00 | 0.85 (0.81-0.90) |  | 4376.71 (<.001) | 98.96 |  |
| German | 7 | 1357 | 0.76-1.00 | 0.89 (0.81-0.97) |  | 210.03 (<.001) | 96.77 |  |
| Hungarian | 5 | 997 | 0.69-0.97 | 0.85 (0.73-0.98) |  | 61.85 (<.001) | 94.70 |  |
| Korean | 6 | 3741 | 0.53-0.88 | 0.76 (0.49-1.03) |  | 265.80 (<.001) | 98.39 |  |
| Mean age | | | | | | | | |
| < 35 | 16 | 1515 | 0.70-1.00 | 0.86 (0.80-0.91) | 0.005 | 300.66 (<.001) | 92.39 | 0.62 (.54) |
| > 35 | 12 | 2561 | 0.53-0.93 | 0.80 (0.70-0.89) |  | 221.92 (<.001) | 94.53 |  |
| Sample size | | | | | | | | |
| < 100 | 26 | 1758 | 0.25-1.00 | 0.81 (0.72-0.90) | 0.005 | 775.37 (<.001) | 99.92 | 0.03 (.86) |
| > 100 | 69 | 28737 | 0.42-1.00 | 0.80 (0.76-0.84) |  | 8371.21 (<.001) | 98.05 |  |
| AVEC Audio/Visual Emotion Challenge, BDI Beck Depression Inventory, BDI-II Beck Depression Inventory-II, CNN Convolutional Neural Network, DAIC-WOZ Distress Analysis Interview Corpus Wizard-of-Oz, DNN Deep Neural Network, DSM Diagnostic and Statistical Manual of Mental Disorders, DSM-IV Diagnostic and Statistical Manual of Mental Disorders, Fourth Edition, HAM-D Hamilton Depression Rating Scale, kNN K-Nearest Neighbours, PHQ-8 Patient Health Questionnaire-8, PHQ-9 Patient Health Questionnaire-9, SVM Support Vector Machine | | | | | | | | |

**Table S11.** Estimated pooled mean of lowest precision by several factors.

| Groups | Number of studies | Sample size | Precision (%) | Pooled mean precision | Heterogeneity measures | | | Test for subgroups differences |
| --- | --- | --- | --- | --- | --- | --- | --- | --- |
|  | Total N | Total N | Range | Mean (%) (95% CI) | Tau^2^ | Q (p-value) | I^2^ (%) | F (p-value) |
| Type of publication | | | | | | | | |
| Journal Article | 44 | 20110 | 0.27-0.97 | 0.66 (0.59-0.73) | 0.003 | 2738.07 (<.001) | 98.46 | 0.58 (0.57) |
| Conference paper | 28 | 6264 | 0.00-0.98 | 0.60 (0.48-0.72) |  | 16789.84 (<.001) | 99.96 |  |
| Speech features | | | | | | | | |
| Spectral features | 66 | 15573 | 0.00-0.98 | 0.63 (0.56-0.70) | 0.003 | 40839.62 (<.001) | 99.88 | 1.25 (.29) |
| Prosodic features | 46 | 11868 | 0.00-0.97 | 0.59 (0.50-0.69) |  | 24801.85 (<.001) | 99.92 |  |
| Source features | 43 | 9590 | 0.00-0.97 | 0.61 (0.52-0.71) |  | 21664.40 (<.001) | 99.93 |  |
| Format features | 31 | 7521 | 0.17-0.97 | 0.59 (0.45-0.73) |  | 17281.59 (<.001) | 99.96 |  |
| Lexical features | 13 | 4698 | 0.42-0.97 | 0.69 (0.55-0.83) |  | 724.45 (<.001) | 98.23 |  |
| Algorithms | | | | | | | | |
| SVM | 16 | 3786 | 0.00-0.89 | 0.61 (0.49-0.74) | 0.002 | 14511.62 (<.001) | 99.39 | 2.19 (.08) |
| CNN | 7 | 1382 | 0.08-0.98 | 0.66 (0.45-0.86) |  | 358.34 (<.001) | 98.20 |  |
| Logistic Regression | 6 | 2483 | 0.27-0.86 | 0.55 (0.20-0.90) |  | 510.23 (<.001) | 98.90 |  |
| Random Forest | 5 | 1025 | 0.40-0.71 | 0.50 (0.35-0.65) |  | 32.84 (<.001) | 91.35 |  |
| Speech-eliciting tasks | | | | | | | | |
| Free speech | 61 | 23878 | 0.00-0.98 | 0.64 (0.57-0.71) | 0.003 | 52968.02 (<.001) | 99.89 | 1.05 (.39) |
| Reading | 25 | 5896 | 0.41-0.95 | 0.72 (0.64-0.80) |  | 1062.77 (<.001) | 96.97 |  |
| Counting | 6 | 2032 | 0.45-0.78 | 0.56 (0.29-0.82) |  | 512.72 (<.001) | 98.13 |  |
| Sustained vowel | 6 | 2343 | 0.45-0.87 | 0.69 (0.21-1.16) |  | 452.31 (<.001) | 99.19 |  |
| Ground truth assessment | | | | | | | | |
| PHQ-8, and PHQ-9 | 47 | 21466 | 0.25-0.97 | 0.63 (0.55-0.72) | 0.003 | 45565.67 (<.001) | 99.91 | 0.84 (.50) |
| BDI, and BDI-II | 12 | 1902 | 0.17-0.78 | 0.60 (0.48-0.73) |  | 259.96 (<.001) | 95.54 |  |
| DSM, DSM-IV | 10 | 1942 | 0.45-0.57 | 0.67 (0.41-0.94) |  | 188.20 (.03) | 97.18 |  |
| HAM-D | 7 | 3001 | 0.78-0.97 | 0.82 (0.67-0.97) |  | 396.91 (<.001) | 97.32 |  |
| Validation approach | | | | | | | | |
| Hold-out | 62 | 25750 | 0.00-0.98 | 0.63 (0.56-0.70) | 0.003 | 54008.15 (<.001) | 99.88 | 2.97 (.06) |
| K-fold | 32 | 6633 | 0.17-0.97 | 0.74 (0.63-0.84) |  | 2190.21 (<.001) | 98.49 |  |
| Dataset | | | | | | | | |
| Hand-crafted | 39 | 6353 | 0.17-0.98 | 0.68 (0.59-0.76) | 0.003 | 1676.76 (<.001) | 97.65 | 1.20 (.32) |
| DAIC-WOZ | 35 | 20171 | 0.00-0.89 | 0.63 (0.53-0.73) |  | 40572.67 (<.001) | 99.94 |  |
| AVEC | 6 | 1761 | 0.44-0.78 | 0.62 (0.48-0.76) |  | 136.29 (<.001) | 95.49 |  |
| MODMA | 5 | 733 | 0.67-0.82 | 0.76 (0.66-0.85) |  | 14.05 (.01) | 71.11 |  |
| Dataset language | | | | | | | | |
| English | 38 | 20789 | 0.00-0.89 | 0.60 (0.51-0.69) | 0.003 | 41007.20 (<.001) | 99.93 | 0.86 (.50) |
| Chinese | 22 | 16381 | 0.36-0.97 | 0.68 (0.59-0.77) |  | 1773.85 (<.001) | 98.78 |  |
| Korean | 7 | 3945 | 0.45-0.95 | 0.70 (0.36-1.02) |  | 526.23 (<.001) | 99.26 |  |
| German | 5 | 1197 | 0.67-0.78 | 0.71 (0.64-0.78) |  | 12.51 (.01) | 63.53 |  |
| Mean age | | | | | | | | |
| < 35 | 13 | 1095 | 0.50-0.97 | 0.75 (0.63-0.87) | 0.003 | 149.81 (<.001) | 93.95 | 2.28 (.11) |
| > 35 | 8 | 2065 | 0.45-0.95 | 0.76 (0.52-1.00) |  | 576.31 (<.001) | 98.34 |  |
| Sample size | | | | | | | | |
| < 100 | 23 | 1532 | 0.17-0.98 | 0.64 (0.52-0.76) | 0.003 | 647.50 (<.001) | 95.71 | 0.002 (.96) |
| > 100 | 50 | 25284 | 0.00-0.95 | 0.64 (0.56-0.71) |  | 50840.26 (<.001) | 99.91 |  |
| AVEC Audio/Visual Emotion Challenge, BDI Beck Depression Inventory, BDI-II Beck Depression Inventory-II, CNN Convolutional Neural Network, DAIC-WOZ Distress Analysis Interview Corpus Wizard-of-Oz, DSM Diagnostic and Statistical Manual of Mental Disorders, DSM-IV Diagnostic and Statistical Manual of Mental Disorders, Fourth Edition, HAM-D Hamilton Depression Rating Scale, PHQ-8 Patient Health Questionnaire-8, PHQ-9 Patient Health Questionnaire-9, SVM Support Vector Machine | | | | | | | | |

**Table S12.** Search string.

Database(s): Ovid MEDLINE(R) ALL 1946 to April 02, 2025

| # | Searches | Results |
| --- | --- | --- |
| 1 | "speech analysis".tw. | 554 |
| 2 | "speech processing".tw. | 2289 |
| 3 | "speech feature".tw. | 89 |
| 4 | "speech features".tw. | 468 |
| 5 | "speech signal".tw. | 1430 |
| 6 | "speech signal processing".tw. | 60 |
| 7 | "speech classification".tw. | 61 |
| 8 | "speech recognition".tw. | 5550 |
| 9 | "speech model".tw. | 42 |
| 10 | "acoustic analysis".tw. | 2119 |
| 11 | "acoustic processing".tw. | 155 |
| 12 | "acoustic feature".tw. | 202 |
| 13 | "acoustic features".tw. | 1712 |
| 14 | "acoustic signal".tw. | 1527 |
| 15 | "acoustic signal processing".tw. | 55 |
| 16 | "acoustic classification".tw. | 36 |
| 17 | "acoustic recognition".tw. | 47 |
| 18 | "acoustic model".tw. | 238 |
| 19 | "vocal analysis".tw. | 74 |
| 20 | "vocal processing".tw. | 25 |
| 21 | "vocal feature".tw. | 8 |
| 22 | "vocal features".tw. | 107 |
| 23 | "vocal signal".tw. | 75 |
| 24 | "vocal signal processing".tw. | 1 |
| 25 | "voice recognition".tw. | 674 |
| 26 | "vocal recognition".tw. | 77 |
| 27 | "voice model".tw. | 14 |
| 28 | "vocal model".tw. | 6 |
| 29 | exp Depression/ | 168186 |
| 30 | depress*.tw. | 617446 |
| 31 | exp Depressive Disorder/ | 128008 |
| 32 | depressive disorder*.tw. | 51975 |
| 33 | 1 or 2 or 3 or 4 or 5 or 6 or 7 or 8 or 9 or 10 or 11 or 12 or 13 or 14 or 15 or 16 or 17 or 18 or 19 or 20 or 21 or 22 or 23 or 24 or 25 or 26 or 27 or 28 | 16106 |
| 34 | 29 or 30 or 31 or 32 | 662674 |
| 35 | 33 and 34 | 210 |
| 36 | limit 35 to (English language and yr="2013 -Current") | 167 |

Database(s): APA PsycInfo 1984 to April 2, 2025

| # | Searches | Results |
| --- | --- | --- |
| 1 | noft("speech analysis") | 494 |
| 2 | noft("speech processing") | 2821 |
| 3 | noft("speech feature") | 51 |
| 4 | noft("speech features") | 301 |
| 5 | noft("speech signal") | 1196 |
| 6 | noft("speech signal processing") | 36 |
| 7 | noft("speech classification") | 40 |
| 8 | noft("speech recognition") | 3752 |
| 9 | noft("speech model") | 47 |
| 10 | noft("acoustic analysis") | 982 |
| 11 | noft("acoustic processing") | 110 |
| 12 | noft("acoustic feature") | 163 |
| 13 | noft("acoustic features") | 1184 |
| 14 | noft("acoustic signal") | 649 |
| 15 | noft("acoustic signal processing") | 14 |
| 16 | noft("acoustic classification") | 14 |
| 17 | noft("acoustic recognition") | 18 |
| 18 | noft("acoustic model") | 95 |
| 19 | noft("vocal analysis") | 41 |
| 20 | noft("vocal processing") | 18 |
| 21 | noft("vocal feature") | 2 |
| 22 | noft("vocal features") | 65 |
| 23 | noft("vocal signal") | 66 |
| 24 | noft("vocal signal processing") | 0 |
| 25 | noft("voice recognition") | 384 |
| 26 | noft("vocal recognition") | 108 |
| 27 | noft("voice model") | 23 |
| 28 | noft("vocal model") | 16 |
| 29 | subject(Depression ) | 242555 |
| 30 | noft(depress*) | 470724 |
| 31 | subject(major depression) | 170855 |
| 32 | noft(depressive disorder*) | 151615 |
| 33 | S1 OR S2 OR S3 OR S4 OR S5 OR S6 OR S7 OR S8 OR S9 OR S10 OR S11 OR S12 OR S13 OR S14 OR S15 OR S16 OR S17 OR S18 OR S19 OR S20 OR S21 OR S22 OR S23 OR S24 OR S25 OR S26 OR S27 OR S28 | 11090 |
| 34 | S29 or S30 or S31 or S32 | 362514 |
| 35 | S33 and S34 | 211 |
| 36 | limit S35 to (English language and yr="2013 -Current") | 154 |

Database(s): Embase 1974 to July 07, 2023

| # | Searches | Results |
| --- | --- | --- |
| 1 | "speech analysis":ti,ab,kw | 877 |
| 2 | "speech processing":ti,ab,kw | 2792 |
| 3 | "speech feature":ti,ab,kw | 108 |
| 4 | "speech features":ti,ab,kw | 564 |
| 5 | "speech signal":ti,ab,kw | 1605 |
| 6 | "speech signal processing":ti,ab,kw | 92 |
| 7 | "speech classification":ti,ab,kw | 68 |
| 8 | "speech recognition":ti,ab,kw | 6227 |
| 9 | "speech model":ti,ab,kw | 56 |
| 10 | "acoustic analysis":ti,ab,kw | 2874 |
| 11 | "acoustic processing":ti,ab,kw | 178 |
| 12 | "acoustic feature":ti,ab,kw | 229 |
| 13 | "acoustic features":ti,ab,kw | 1872 |
| 14 | "acoustic signal":ti,ab,kw | 1703 |
| 15 | "acoustic signal processing":ti,ab,kw | 86 |
| 16 | "acoustic classification":ti,ab,kw | 36 |
| 17 | "acoustic recognition":ti,ab,kw | 51 |
| 18 | "acoustic model":ti,ab,kw | 257 |
| 19 | "vocal analysis":ti,ab,kw | 103 |
| 20 | "vocal processing":ti,ab,kw | 31 |
| 21 | "vocal feature":ti,ab,kw | 8 |
| 22 | "vocal features":ti,ab,kw | 112 |
| 23 | "vocal signal":ti,ab,kw | 85 |
| 24 | "vocal signal processing":ti,ab,kw | 0 |
| 25 | "voice recognition":ti,ab,kw | 915 |
| 26 | "vocal recognition":ti,ab,kw | 90 |
| 27 | "voice model":ti,ab,kw | 1 |
| 28 | "vocal model":ti,ab,kw | 5 |
| 29 | “depression”/exp | 729710 |
| 30 | "depress*":ti,ab,kw | 849698 |
| 31 | “depressive disorder”/exp | 729710 |
| 32 | "depressive disorder*":ti,ab,kw | 76398 |
| 33 | #1 OR #2 OR #3 OR #4 OR #5 OR #6 OR #7 OR #8 OR #9 OR #10 OR #11 OR #12 OR #13 OR #14 OR #15 OR #16 OR #17 OR #18 OR #19 OR #20 OR #21 OR #22 OR #23 OR #24 OR #25 OR #26 OR #27 OR #28 | 18925 |
| 34 | #29 OR #30 OR #31 OR #32 | 1085777 |
| 35 | #33 and #34 | 348 |
| 36 | #34 AND #35 AND [english]/lim AND [2023-2025]/py | 282 |

Database(s): CINAHL April 02, 2025

| # | Searches | Results |
| --- | --- | --- |
| S1 | MW Speech Acoustics | 4205 |
| S2 | AB "speech analysis" | 145 |
| S3 | AB "speech processing" | 462 |
| S4 | AB "speech feature" | 20 |
| S5 | AB "speech features" | 91 |
| S6 | AB "speech signal" | 299 |
| S7 | AB "speech signal processing" | 11 |
| S8 | AB "speech classification" | 5 |
| S9 | AB "speech recognition" | 1910 |
| S10 | AB "speech model" | 14 |
| S11 | AB "acoustic analysis" | 685 |
| S12 | AB "acoustic processing" | 15 |
| S13 | AB "acoustic feature" | 22 |
| S14 | AB "acoustic features" | 242 |
| S15 | AB "acoustic signal" | 182 |
| S16 | AB "acoustic signal processing" | 7 |
| S17 | AB "acoustic classification" | 3 |
| S18 | AB "acoustic recognition" | 1 |
| S19 | AB "acoustic model" | 23 |
| S20 | AB "vocal analysis" | 25 |
| S21 | AB "vocal processing" | 0 |
| S22 | AB "vocal feature" | 1 |
| S23 | AB "vocal features" | 15 |
| S24 | AB "vocal signal" | 8 |
| S25 | AB "vocal signal processing" | 0 |
| S26 | AB "voice recognition" | 154 |
| S27 | AB "vocal recognition" | 1 |
| S28 | AB "voice model" | 7 |
| S29 | AB "vocal model" | 3 |
| S30 | MW Depression | 158446 |
| S31 | AB depress* | 165797 |
| S32 | AB Depressive Disorder | 13960 |
| S33 | AB depressive disorder* | 14124 |
| S34 | S1 OR S2 OR S3 OR S4 OR S5 OR S6 OR S7 OR S8 OR S9 OR S10 OR S11 OR S12 OR S13 OR S14 OR S15 OR S16 OR S17 OR S18 OR S19 OR S20 OR S21 OR S22 OR S23 OR S24 OR S25 OR S26 OR S27 OR S28 OR S29 | 7682 |
| S35 | S30 OR S31 OR S32 OR S33 | 228319 |
| S36 | S34 AND S35 | 61 |
| S36 | Limiters - Published Date: 20130101-20251231 and english | 50 |

| IEEE Xplore | (("Abstract":"speech analysis") OR ("Abstract":"speech processing") OR ("Abstract":"speech feature") OR ("Abstract":"speech features") OR ("Abstract":"speech signal") OR ("Abstract":"speech signal processing") OR ("Abstract":"speech classification") OR ("Abstract":"speech recognition") OR ("Abstract":"speech model") OR ("Abstract":"acoustic analysis") OR ("Abstract":"acoustic processing") OR ("Abstract":"speech analysis") OR ("Abstract":"acoustic feature") OR ("Abstract":"acoustic features") OR ("Abstract":"acoustic signal") OR ("Abstract":"acoustic signal processing") OR ("Abstract":"acoustic classification") OR ("Abstract":"acoustic recognition") OR ("Abstract":"acoustic model") OR ("Abstract":"vocal analysis") OR ("Abstract":"vocal processing") OR ("Abstract":"vocal feature") OR ("Abstract":"vocal features") OR ("Abstract":"vocal signal") OR ("Abstract":"acoustic features") OR ("Abstract":"vocal signal processing") OR ("Abstract":"voice recognition") OR ("Abstract":"vocal recognition") OR ("Abstract":"voice model") OR ("Abstract":"vocal model")) AND (("Abstract":depress*) OR ("Abstract":depressive disorder*)) filters applied: 2013-2025 | 97 |
| --- | --- | --- |
| ACM Digital Library | [[Abstract: "speech analysis"] OR [Abstract: "speech processing"] OR [Abstract: "speech feature"] OR [Abstract: "speech features"] OR [Abstract: "speech signal"] OR [Abstract: "speech signal processing"] OR [Abstract: "speech classification"] OR [Abstract: "speech recognition"] OR [Abstract: "speech model"] OR [Abstract: "acoustic analysis"] OR [Abstract: "acoustic processing"] OR [Abstract: "acoustic feature"] OR [Abstract: "acoustic features"] OR [Abstract: "acoustic signal"] OR [Abstract: "acoustic signal processing"] OR [Abstract: "acoustic classification"] OR [Abstract: "acoustic recognition"] OR [Abstract: "acoustic model"] OR [Abstract: "vocal analysis"] OR [Abstract: "vocal processing"] OR [Abstract: "vocal feature"] OR [Abstract: "vocal features"] OR [Abstract: "vocal signal"] OR [Abstract: "vocal signal processing"] OR [Abstract: "voice recognition"] OR [Abstract: "vocal recognition"] OR [Abstract: "voice model"] OR [Abstract: "vocal model"]] AND [[Abstract: depress*] OR [Abstract: "depressive disorder*"]] AND [E-Publication Date: (01/01/2013 TO 31/12/2025)] | 20 |
| Scopus | ( TITLE-ABS-KEY ( "speech analysis" OR "speech processing" OR "speech feature" OR "speech features" OR "speech signal" OR "speech signal processing" OR "speech classification" OR "speech recognition" OR "speech model" OR"acoustic analysis" OR "acoustic processing" OR "speech analysis" OR "acoustic feature" OR "acoustic features" OR"acoustic signal" OR "acoustic signal processing" OR "acoustic classification" OR "acoustic recognition" OR "acoustic model" OR "vocal analysis" OR "vocal processing" OR "vocal feature" OR "vocal features" OR "vocal signal" OR"acoustic features" OR "vocal signal processing" OR "voice recognition" OR "vocal recognition" OR "voice model" OR"vocal model" ) ) AND ( TITLE-ABS-KEY ( depress* OR depressive AND disorder* ) ) AND ( LIMIT-TO ( PUBYEAR , 2013 )OR LIMIT-TO ( PUBYEAR , 2014 ) OR LIMIT-TO ( PUBYEAR , 2015 ) OR LIMIT-TO ( PUBYEAR , 2016 ) OR LIMIT-TO (PUBYEAR , 2017 ) OR LIMIT-TO ( PUBYEAR , 2018 ) OR LIMIT-TO ( PUBYEAR , 2019 ) OR LIMIT-TO ( PUBYEAR , 2020 ) ORLIMIT-TO ( PUBYEAR , 2021 ) OR LIMIT-TO ( PUBYEAR , 2022 ) OR LIMIT-TO ( PUBYEAR , 2023 ) OR LIMIT-TO ( PUBYEAR ,2024 ) OR LIMIT-TO ( PUBYEAR , 2025 ) ) AND ( LIMIT-TO ( LANGUAGE , "english" ) ) | 475 |
| Google Scholar | ("speech analysis" OR "speech processing" OR "speech signal processing" OR "speech recognition" OR "acoustic analysis" OR "acoustic features" OR "acoustic signal") AND (depress*  OR  depressive  AND disorder*) | 100 |
